# Solution structure of a peptide-conjugated aggregation-induced-emission (AIE) fluorophore bound to a protein

**DOI:** 10.1039/d6cb00109b

**Published:** 2026-10-15

**Authors:** Daniel Seifert, Matthias Hayduk, Torsten Herrmann, David Podlesainski, Jill Rohde, Jürgen Linders, Markus Kaiser, Peter Bayer, Jens Voskuhl, Christine Beuck

**Affiliations:** a Structural and Medicinal Biochemistry, University of Duisburg-Essen Essen Germany christine.beuck@uni-due.de; b Faculty of Chemistry (Organic Chemistry), University of Duisburg-Essen Essen Germany jens.voskuhl@uni-due.de; c Institute of Structural Biology, Grenoble Alpes University Grenoble France; d Chemical Biology, University of Duisburg-Essen Essen Germany; e Physical Chemistry, University of Duisburg-Essen Essen Germany

## Abstract

Here, we present a class of peptide-conjugated fluorophores based on aromatic thioethers with aggregation-induced emission (AIE) properties for specific recognition of small proteins and also the first solution NMR structure of an AIE ligand specifically bound to a protein. Binding of the hPin1-WW domain as a model protein resulted in a significant fluorescence increase, making these AIE-peptide fluorophores suitable as direct readout probes for specific protein interactions. Autofluorescence and protein-binding properties were elucidated as a function of peptide length and fluorophore concentration. While an AIE fluorophore with two conjugated peptides shows an enhanced fluorescence increase upon protein binding, a one-armed derivative enabled us to solve the structures of the free AIE-peptide and its complex. In the absence of a protein, the AIE-peptide conjugate shows a curled-up conformation that still allows rotation of the aromatic rings of the AIE-core, thus resulting in low auto-fluorescence. In contrast, the AIE-peptide unfurls upon binding, allowing the hPin1-WW domain to interact with its peptide recognition sequence. The aromatic rings of the AIE-core form additional contacts with the protein surface, which restricts their motions and thus results in the observed fluorescence increase.

## Abstract 2

NMR structures of free and protein-bound aggregation-induced-emission (AIE) fluorophores conjugated to a peptide explain the fluorescence increase upon binding.

## Graphical abstract

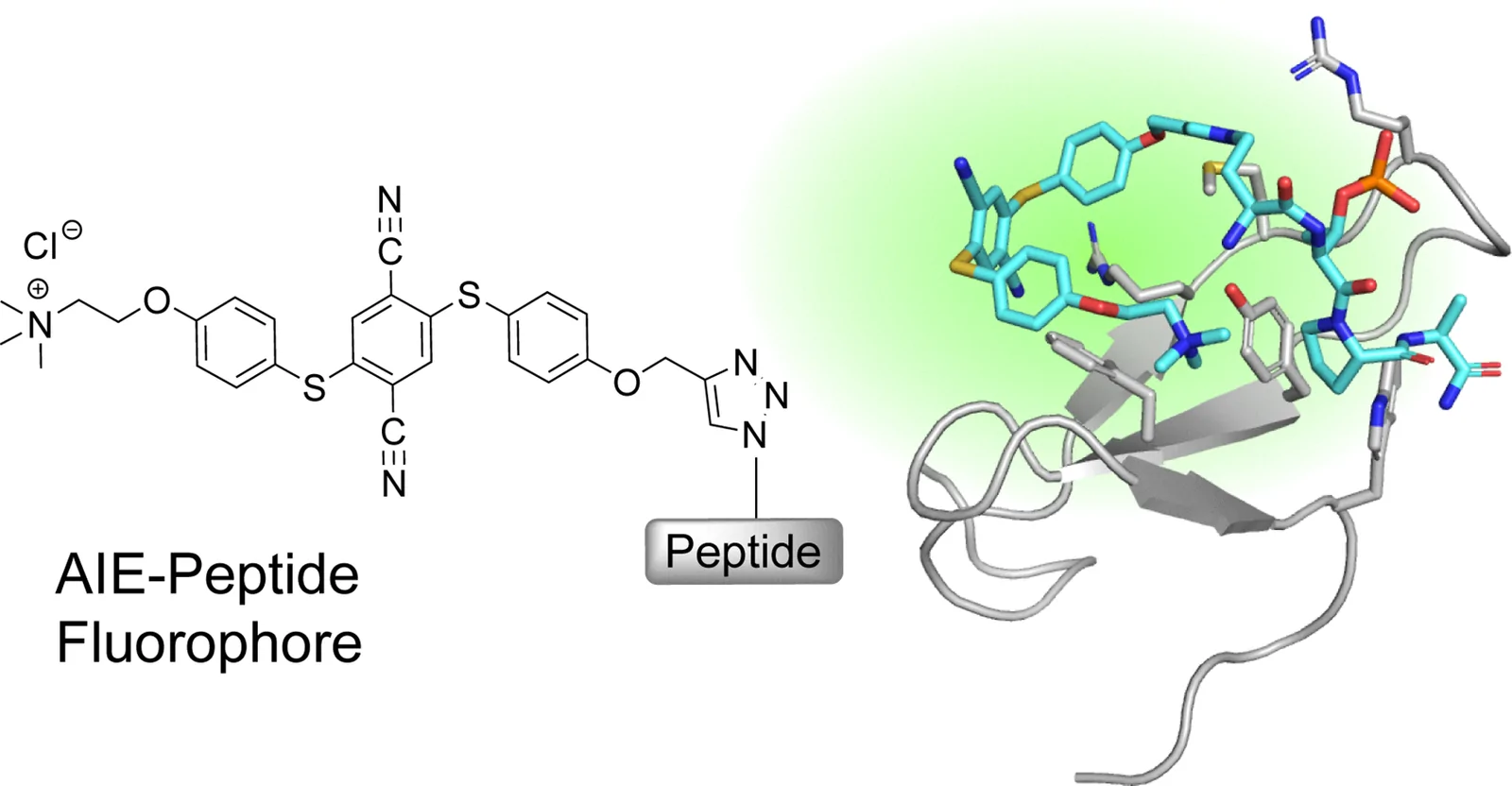

## Introduction

Ever since the discovery that emission can not only occur from molecularly dissolved luminophores around 100 years ago by Schmidt *et al.*,^1^ the fluorescence emitted from an aggregated state was named aggregation-induced emission (AIE) by Tang and co-workers^2^ in 2001 and has experienced a renaissance in the past 20 years.^3,4^ Here, emission occurs from motion-restricted luminophores enabling radiative decay by suppression of molecular motions such as rotations and vibrations. Other than the name AIE would suggest, emission also occurs from monomers and not only from aggregates if the corresponding luminophore is, for example, trapped in a binding pocket of an enzyme or protein.^5,6^ This behaviour makes these compounds, typically equipped with phenyl-rotors, ideal candidates for determining binding and inhibition events.^7^ Other than conventional luminophores, such as rhodamine, fluorescein and Alexa™ derivatives, which feature an always “on” behaviour independent of their binding state, AIE luminophores can be used to track binding events upon switching from the luminescence “off” state to the “on” state, giving a direct readout possibility. In this regard, numerous studies have already shown the potential of AIE-active compounds, for example, in protein detection arrays^8^ or as markers for protein sulfonation.^9^ Aromatic thioethers^10,11^ were found to be a versatile AIE-active platform for numerous applications ranging from materials science^12,13^ to biomedical applications.^14,15^ Very recently, we reported that symmetric AIE-active ligands bearing molecular tweezers are found to target the cancer relevant protein Taspase1^16^ or are able to block the central pore of the protease p97^17^ and the binding can be tracked by a luminescence response. In addition to the specific binding of the tweezers to basic amino acids on the protein surface, molecular modelling suggests additional interactions of the aromatic AIE-core with the protein surface, which in turn limit AIE motion and would explain the observed fluorescence increases. However, so far, the structure of an AIE-ligand:protein complex has not been published.

Versatile AIE-active scaffolds can be equipped with different protein recognition moieties. In this work, we coupled a two-armed AIE-core and a one-armed AIE-core with natural peptide sequences of different lengths containing a phosphothreonine–proline (pThr-Pro) binding motif for the WW domain^18,19^ of the human peptidyl prolyl *cis*–*trans* isomerase (PPIase) Pin1^20^ (hPin1-WW). hPin1-WW is a structurally and biochemically well-characterized small protein domain. Structures were solved using both X-ray crystallography and NMR spectroscopy and include the apo protein^21–24^ as well as hPin1-WW bound to small peptide sequences derived from the known biological target proteins CDC25,^24^ Tau,^24^ Smad^25^ and CPEB,^26^ which allow a comparison of the ligand binding mode.

In this work, we characterize the binding and induced fluorescence changes of the peptide-conjugated AIEs to hPin1-WW and present the first solution NMR structure of an AIE-active ligand bound to a protein and free in solution, proving that the aromatic rings of the AIE-core interact with the protein surface which restricts their motion and thus results in a fluorescence “on” signal.

## Results and discussion

In this work, we designed AIE fluorophores conjugated to peptide sequences carrying the recognition motif for the hPin1-WW domain as probes for quantitative binding experiments and structural studies.

We chose a *para*-di-substituted aromatic phthalonitrile thioether AIE-core, since initial studies revealed a good balance between the size and the photophysical performance^10^ with one^27^ or two^16^ alkyne groups that can be functionalised *via* click chemistry. This AIE-core was coupled with one (F_1_1) or two (F_2_1–F_2_6) peptides to compare whether binding of one or two proteins is required to restrict molecular motions of the AIE sufficiently to yield a detectable fluorescence increase upon binding. Peptides contained the hPin1-WW recognition motif pThr-Pro and a clickable N-terminal azido-homoalanine (Aha). These two features were separated by (Ala–Gly)_*n*_ repeats of increasing length (*n* = 0–3) to find the optimum linker length. If the AIE-core is placed too close to the protein recognition site, it might interfere with binding. If the distance is too large, the AIE motion might not be restricted upon protein binding, resulting in insufficient fluorescence increase. In addition, two AIE-peptides with a mutated hPin1-WW recognition site (pThr to Thr, F_2_5 and pThr-Pro to Thr-Gly, F_2_6) were designed as non-binding controls.

### Synthesis

The used alkyne functionalized AIE luminophores (Fig. S0–S1) have been described beforehand by us^16,27^ and can be synthesized in short linear synthetic routes. Peptides P1–P6 with an N-terminal azido-homoalanine (Aha) residue were synthesized *via* standard solid phase peptide synthesis using FMOC chemistry, deprotected and purified by HPLC. The azido-functionalized peptides were coupled with the alkyne-bearing AIE-cores, yielding peptide-conjugated AIE fluorophores F_1_1 and F_2_1–F_2_6 (Fig. 1) in a Cu(i) catalysed click reaction. The TFA ions were exchanged with chloride to avoid possible interference of protein binding by trifluoroacetate (TFA) ions present after HPLC purification. The absence of TFA in the final peptide-conjugated AIE fluorophores was confirmed by ^19^F-NMR spectroscopy.

**Fig. 1:**
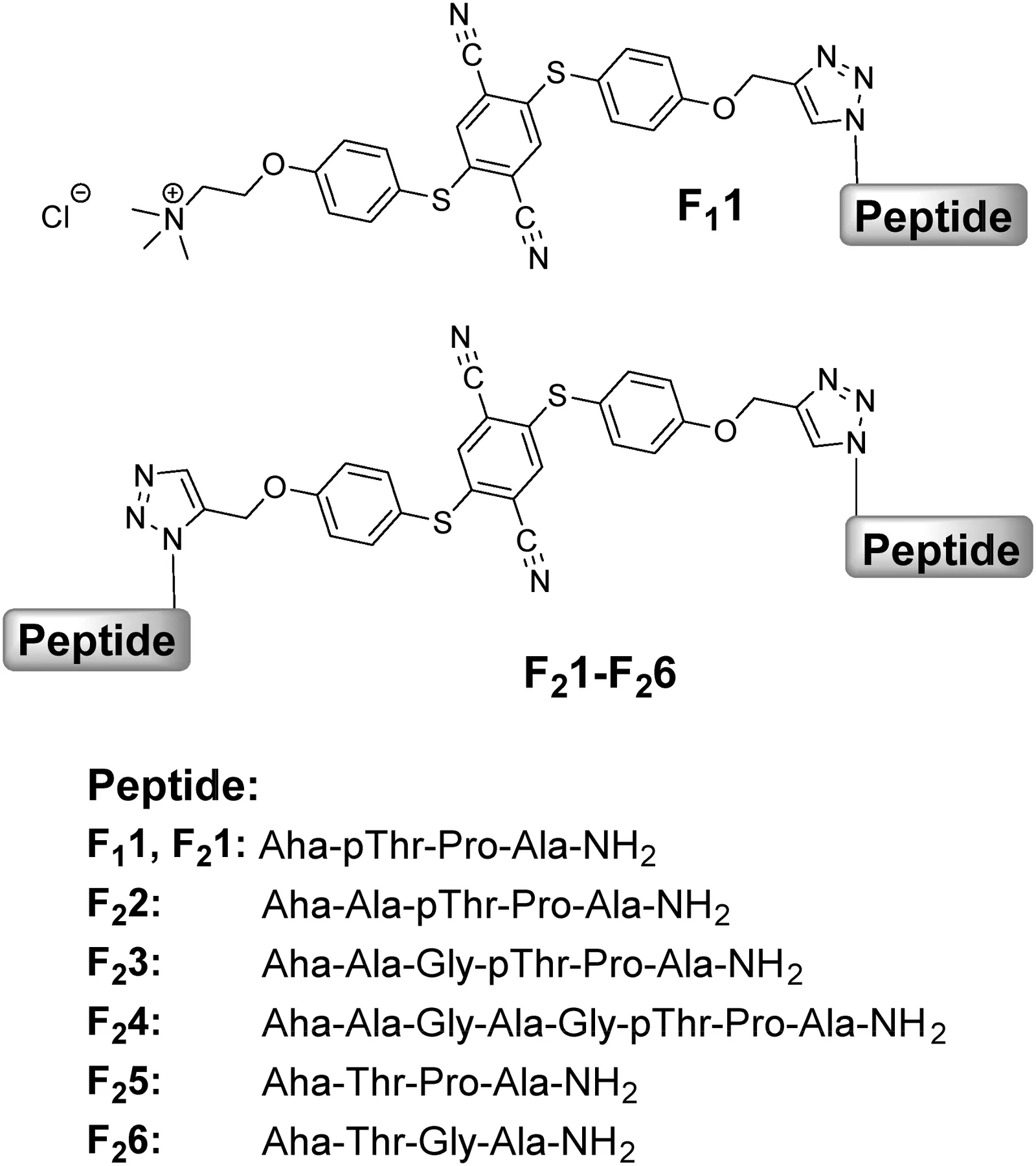
Peptide-conjugated AIE fluorophores (Aha = homoalanine; pThr = phosphothreonine, –NH_2_ = amidated C-terminus). F_1_1 carries one peptide, while F_2_1–F_2_6 carry two identical peptide arms.

### Solubility and auto-fluorescence properties

AIE fluorophores suitable for aggregation-induced emission enhancement (AIEE) upon protein binding should be soluble in aqueous buffer without aggregating and exhibit a low auto-fluorescence in the absence of proteins (Fig. S1). Since the aromatic AIE-core is hydrophobic in nature, aggregation, and thus, a fluorescence increase, has been observed for many AIEs in aqueous solutions.^3,10,28,29^F_1_1 and F_2_1–F_2_6 yielded clear solutions in H_2_O, HEPES or PBS buffer up to concentrations of 5 mM to >20 mM, which was confirmed by measuring their turbidity using UV/Vis. However, even the formation of soluble aggregates causes an increased auto-fluorescence and can potentially interfere with protein binding. While no precipitate was observed, concentrations of >200 µM showed a visible fluorescence gradient along the height of the cuvette with stronger fluorescence at the bottom, consistent with a settling of aggregates (Fig. S1a). Furthermore, we measured the auto-fluorescence of the soluble fluorophores at different concentrations in H_2_O, PBS, and HEPES buffer to determine their solubility threshold concentration (Fig. S1c–e). Low auto-fluorescence indicates solubilized AIE-peptides, while soluble aggregates that form at higher concentrations cause an increase in fluorescence. Up to 200 µM, F_1_1 and F_2_1–F_2_4 showed only a small increase in fluorescence intensity, while higher concentrations caused a significant increase. HEPES buffer showed a slight advantage over PBS. Therefore, fluorescence interaction studies with proteins were carried out with AIE-peptide concentrations between 10 and 200 µM in HEPES. The control fluorophores F_2_5 and F_2_6, where the hPin1-WW binding motif pThr-Pro was mutated, showed a steep increase in auto-fluorescence already at the lowest concentrations and were therefore not used in protein binding experiments.

### Solution structure of AIE-peptide F_1_1 with one peptide arm

Short peptides are usually flexible and devoid of secondary structure. Interestingly, during the NMR characterisation of the AIE-peptides, we observed surprisingly many signals in the NOESY spectrum between the AIE-core and all amino acid residues, as well as between non-adjacent amino acids. NOESY cross-peaks indicate a proton–proton distance of less than 5–6 Å, suggesting a compact, rather than elongated, structure for the AIE-peptides. Therefore, we solved the solution NMR structure of the main conformer of the one-armed AIE-peptide F_1_1 (Fig. 2 and Fig. S2; for statistics see Table S1).

**Fig. 2:**
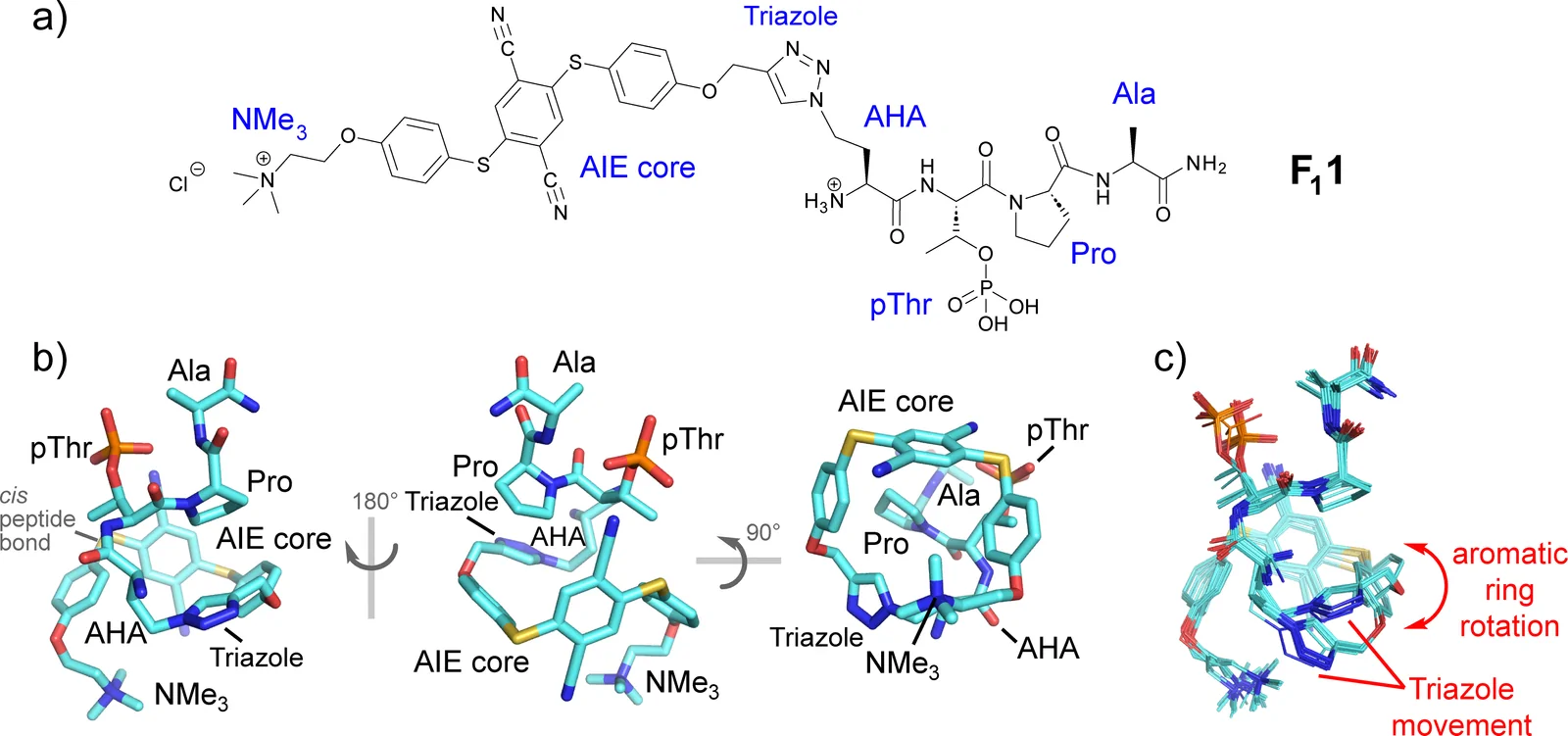
NMR solution structure of the one-armed peptide-conjugated AIE F_1_1 (pdb 29KP/pdb_000029KP). (a) Chemical structure of F_1_1. (b) The AIE-core, consisting of three aromatic rings, adopts a C-shape. The terminal trimethyl-ammonium group is tucked underneath. The peptide is curled up on top of the AIE-core such that the proline residue can form ring stacking interactions with the aromatic AIE rings, while the charged phosphothreonine is exposed to the solvent. (c) The ensemble of the 30 lowest energy structures (shown as a wireframe) shows the ring rotation of the proximal aromatic AIE ring and the adjusting motion of the neighbouring triazole group.

The aromatic rings of the AIE-core adopt a C-shaped conformation. The peptide arm is curled up in a helical fashion above the aromatic core, such that the hydrophobic proline ring interacts with the proximal (closest to the peptide) and central aromatic rings. Such CH–π interactions of proline with aromatic rings are common in peptides and intrinsically disordered proteins (IDPs) with an Xaa–Pro–Aro motif (Xaa = any amino acid, Aro = Trp, Tyr, Phe, His); this weak interaction can stabilize a *cis* conformation of the Xaa–Pro peptide bond.^30–32^ Furthermore, proline-binding protein domains,^33^ such as WW, SH3, EVH1, GYF and UEV domains, as well as profilin, feature aromatic residues lining their proline binding sites.

The peptide bond between the fluorophore-conjugated homoalanine (DS) and pThr adopts a *cis* conformation which enables the tight turn of the peptide chain. The trimethylammonium group at the distal end of F_1_1 is tucked underneath the C-shaped AIE-core. This compact structure presents the charged phosphate and trimethylammonium groups to the solvent, shielding the hydrophobic proline and aromatic residues.

The structural ensemble (Fig. 2c) shows the proximal ring in different conformations, rotating around its *p*-substituted ether and thioether bonds. The triazole ring adjusts the position accordingly to accommodate the proximal ring position. The central ring is observed in two conformations that represent a 180° flip, distinguishable by the position of the two nitrile substituents. The free rotation of the proximal ring explains why the auto-fluorescence of these AIE-peptide conjugates in the absence of protein is still low.

The NOESY peak pattern of the two-armed fluorophore F_2_1 is very similar to the one observed for F_1_1 (Fig. S2c). This suggests a similar curled-up structure, likely with one peptide above and one below the AIE-core. Unfortunately, a structure calculation is impossible due to the symmetry of the molecule, which causes chemical shifts for both identical peptide arms to be degenerate. Thus, intramolecular inter-peptide and intra-peptide NOEs cannot be distinguished.

A minor set of NMR signals (∼18% for F_1_1, see NMR in the SI), which are common for Pro-containing peptides, are attributed to *cis*–*trans* isomers of the peptidyl–prolyl bond that isomerizes very slowly (10^−3^–10^−2^ s^−1^)^34^ in the absence of a PPIase, and/or the homoalanine–pThr peptide bond. The NOESY spectrum of F_1_1 shows exchange crosspeaks between the two conformers, further supporting the slow exchange time scale.

There are no additional sets of signals for the aromatic protons, indicating that the ring rotation occurs in the intermediate-to-fast conformational exchange regime. Namely, the aromatic H atoms of the proximal and distal rings, which are symmetry-related relative to the ring rotation axis, all show one doublet. Thus, we can estimate that the ring flips occur on a µs to lower ms time scale, which corresponds to flipping rates of buried aromatic rings in proteins.^35^

### Aggregation-induced emission enhancement (AIEE) for the quantitative characterisation of protein binding *via* direct read-out

Recognition of the pThr-Pro motif in F_1_1 and F_2_1–F_2_4 by the hPin1-WW domain was confirmed by NMR, titrating peptide-conjugated AIE fluorophores to ^15^N-labelled hPin1-WW (Fig. 3b–d and Fig. S3; spectra for F_2_1–F_2_4 not shown). Specific binding results in signal shifts for protein residues involved in the interaction. Pronounced signal shifts were observed for residues K13–S16 and Q33–E35. The same pattern was also observed upon binding of the control peptide ApTPA (P0) without an AIE-core to ^15^N-hPin1-WW. The peptide arms of the AIE fluorophores and the unmodified peptide both interact with hPin1-WW in the same manner. The binding curves obtained by plotting the chemical shift perturbations for the interacting residues Q33, W34 and E35 as a function of AIE-peptide concentration yield average *K*_D_ values between 20 and 42 µM for F_1_1 and F_2_1–F_2_4 (Table 1 and Table S2), which lie in the same range as those of the P0 control without an AIE-core (*K*_D_ = 18.2 ± 0.2 µM), confirming specific binding of hPin1-WW to the peptide moieties.

**Fig. 3:**
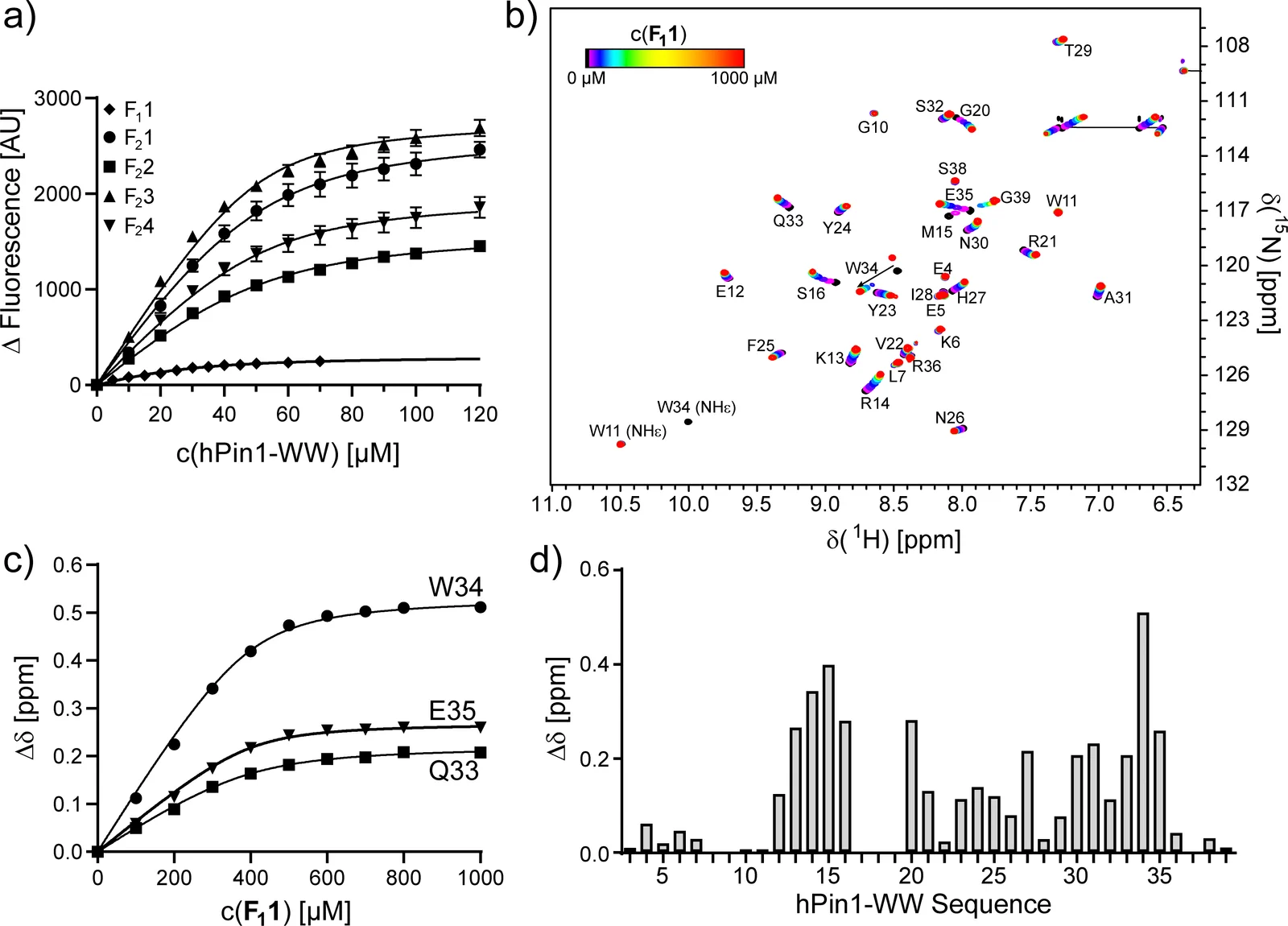
Specific binding of hPin1-WW to peptide-conjugated AIEs. (a) Fluorescence binding curves of two-armed AIE-peptides F_2_1–F_2_4 and one-armed AIE-peptide F_1_1 (25 µM). The AIE fluorescence turn-on signal upon protein binding is well-suited to determine dissociation constants. (b) NMR titration of ^15^N-labelled hPin1-WW (400 µM) with F_1_1. Overlay of ^15^N-HSQC spectra, colour-coded by F_1_1 concentration. Shifting of signals indicates that the corresponding residues are involved in ligand binding. (c) Binding curves for selected residues from the NMR titration. The equivalence point lies at 400 µM because one protein can bind to the one-armed F_1_1 AIE-peptide. (d) Chemical shift perturbation Δ*δ* by residue of hPin1-WW with 1 mM F_1_1 compared to hPin1-WW alone.

**Table 1:** *K*_D_ values of F_1_1 and F_2_1–F_2_4 and control peptide (P0 or FAM-P0) binding to hPin1-WW determined by NMR and fluorescence. Errors of NMR *K*_D_s represent the standard error of the average from binding curves of selected residues (see Table S2). Errors of fluorescence *K*_D,app_ represent the standard error of the fit of the averaged datasets (also see Table S3)

AIE-peptide or peptide	NMR^a^		Fluorescence	
	*K*_D_ [µM]	*n*	*K*_D,app_ [µM]	*n*
F_1_1	27 ± 8	1	12 ± 4^b^	1
F_2_1	27 ± 8	2	4.8 ± 0.9^b^	2
F_2_2	42 ± 10	2	8.0 ± 0.8^b^	2
F_2_3	30 ± 3	2	1.6 ± 0.4^b^	2
F_2_4	20 ± 2	2	2.7 ± 0.7^b^	2
P0	18.2 ± 0.2^c^	1	5 ± 2^d^	1
FAM-P0	n.d.^e^		0.93 ± 0.06^f^	1

a Average *K*_D_ from residues Q33, W34, and E35.

b *c*(AIE fluorophore) = 25 µM.

c NMR titration with P0 without fluorophore.

d hPin1-WW Trp fluorescence (1 µM).

e Not determined.

f Fluorescence anisotropy titration with FAM-P0 (100 nM).

We then tested whether F_1_1 and F_2_1–F_2_4 are suitable to quantify binding of hPin1-WW in fluorescence titrations (Fig. 3a, Fig. S4–S7 and Table S3). If the internal mobility of the AIE-core is restricted upon protein binding, a fluorescence increase is expected. This allows a direct fit of the binding curve, yielding *K*_D_ values. However, potential aggregation could also hinder intramolecular motions and thus cause an additional, unspecific increase in fluorescence intensity. Furthermore, AIE aggregates might sterically hinder protein binding which reduces the observed protein:AIE-peptide ratio and binding affinity.

We titrated hPin1-WW to different concentrations (10 µM–200 µM) of F_1_1 and F_2_1–F_2_4 and measured the AIE fluorescence intensity and the wavelength of the emission maximum as a function of hPin1-WW concentration. All titrations yield hyperbolic binding curves. However, after binding reaches saturation, a steady increase of the fluorescence signal (Fig. S4a) along with a hypsochromic shift of the emission maximum (Fig. S4b) is observed, indicating the beginning of aggregation.

Therefore, binding curves were only evaluated up to the point of saturation (Fig. S5). These curves were fitted with a binding model that yields the *K*_D_ and stoichiometry factor *n* (eqn (S1) in the SI); *n* describes the number of protein molecules bound per AIE-peptide; *n* is expected to be *n* = 1 for F_1_1 with one peptide arm and *n* = 2 for the two-armed AIE-peptides F_2_1–F_2_4.

The two-armed AIE-peptides yielded good binding curves with the expected 2 : 1 stoichiometry and low µM *K*_D_ values for the lower fluorophore concentrations (F_2_1, F_2_2 up to 50 µM; F_2_3, F_2_4 up to 25 µM; Fig. 3a and Fig. S5 and Table S3), with higher concentrations resulting in a better signal : noise ratio as expected. However, at higher fluorophore concentrations, a good fit could only be obtained by decreasing the stoichiometry by a factor of 2–6 (Table S3), likely due to aggregation which renders some binding sites inaccessible. As a result of overestimating the accessible binding sites, the apparent dissociation constants increase as well. The longer the Gly–Ala linker between the AIE-core and the pThr-Pro motif, the smaller the fluorophore concentration where aggregation is observed. Thus, linkers as short as possible are most favourable.

The fluorescence amplitude of F_1_1 (with one peptide) upon protein binding was 5–9-fold lower compared to that of the two-armed fluorophores. The AIE-core with a trimethylammonium group (replacing the second peptide) exhibits a lower emission intensity. F_1_1 titrations with hPin1-WW resulted in good fits with the expected 1 : 1 stoichiometry and low µM *K*_D_s for fluorophore concentrations up to 100 µM (Fig. S6 and Table S3). Higher F_1_1 concentrations did not yield usable binding curves, likely due to fluorophore aggregation. In our experience, *K*_D_s measured using fluorescence generally appear lower than the values obtained using NMR due to intrinsic differences between the methods, like signal-giving component, read-out method, set-up, and concentrations used. The fluorescence assay, in contrast to the NMR titrations, is very sensitive to AIE aggregates because AIE fluorescence is monitored and directly affected by aggregation. With regard to NMR, the signal results from the ^15^N-labelled protein while ligands are invisible. Furthermore, the NMR experiment starts with an excess of protein. Thus, the added AIE-peptides are bound directly, which shifts the aggregation equilibrium towards the free state.

We synthesized AIE-peptides with different lengths, placing the hPin1-WW binding site at varying distances from the AIE-core to elucidate whether (a) a too small distance might result in steric hindrance and impede protein binding, or (b) a too long distance might not restrict fluorophore movement sufficiently to induce AIEE fluorescence. AIE-peptides F_1_1 and F_2_1–F_2_4 at concentrations of 25 µM or 50 µM were titrated with hPin1-WW (Fig. 3a and S7). No increase in *K*_D_ is observed for short linkers, indicating that the AIE-core does not sterically hinder protein binding even for the shortest linker. Furthermore, no clear trend of observed fluorescence amplitudes was observed with respect to the linker length. However, at 50 µM fluorophore concentration, good fits for the longer constructs F_2_3 and F_2_4 could only be achieved allowing a stoichiometry factor *n* < 2, which indicates aggregation of the unbound AIE-peptides. Thus, AIE-peptide fluorophores with shorter peptides are preferred due to better solubility.

Overall, the tested AIE-peptides constitute excellent direct-readout probes for the quantification of protein binding in a concentration range of 10–50 µM. Linker length did not affect the fluorescence amplitude systematically upon protein binding. Shorter linkers were superior because longer ones showed a higher propensity to form soluble aggregates. Fluorophores with two peptides show a larger fluorescence increase upon protein binding compared to F_1_1 with one peptide and are thus preferred probes for binding assays.

### NMR solution structure of F_1_1 bound to hPin1-WW

To elucidate whether the AIE fluorophore is restricted in its internal motions due to specific contacts with the protein or simply steric hindrance, we solved the NMR solution structure of the hPin1-WW domain in complex with AIE-peptide F_1_1, forming a 1 : 1 complex (Fig. 4; for statistics see Table S4; for a detailed per-residue analysis of the interface performed with PISA^36^ see Table S5 and Fig. S9).

**Fig. 4:**
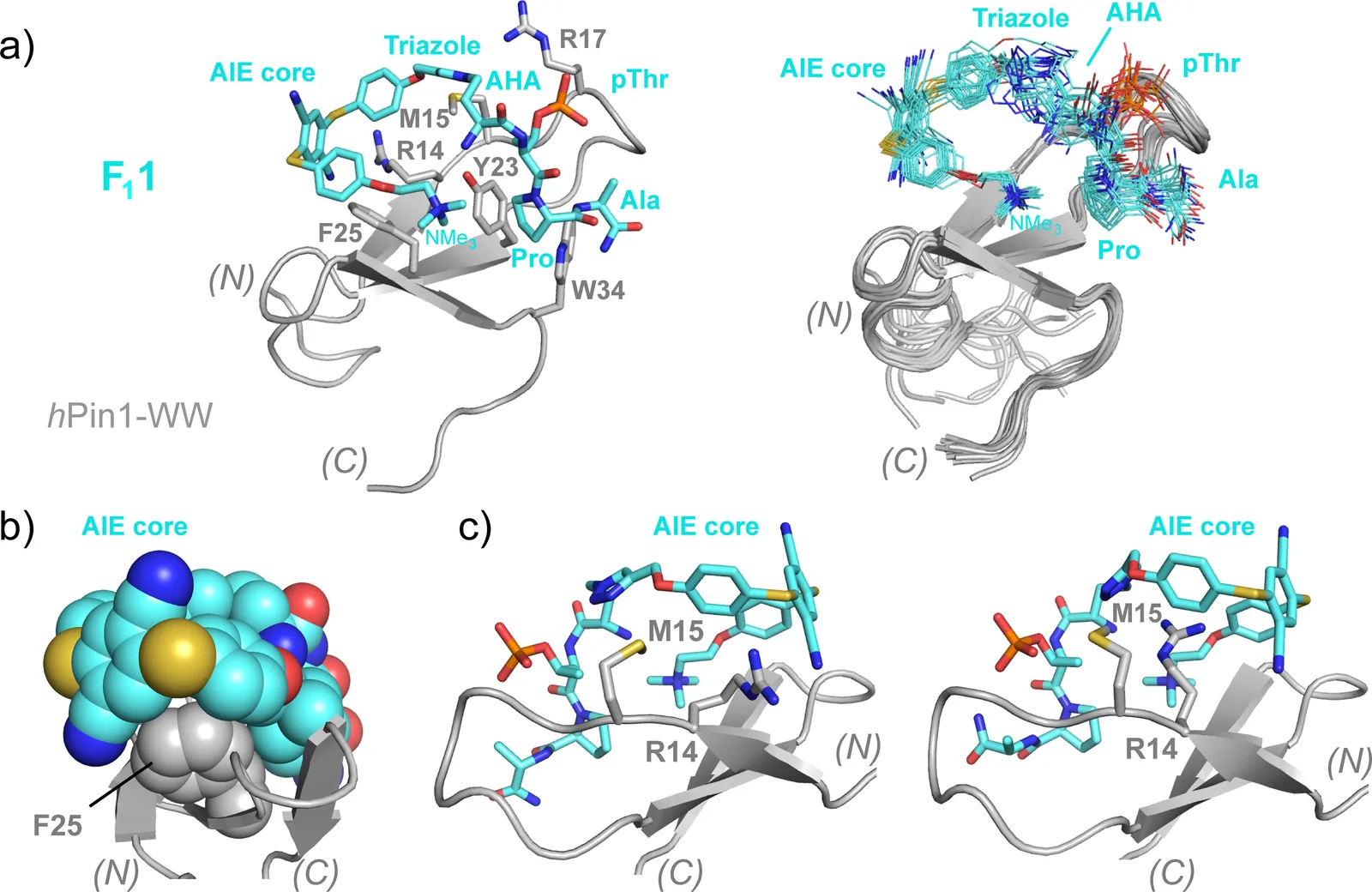
NMR solution structure of AIE-peptide F_1_1 bound to hPin1-WW (pdb 29KQ/pdb_000029KQ). (a) Lowest-energy structure of hPin1-WW (grey, cartoon, N- and C-terminus labelled in parentheses) with F_1_1 (cyan, sticks) with interacting protein residues shown as sticks. The AIE-peptide is draped over the top of the WW domain's β-sheet. The pThr-Pro motif is bound by the hPin1-WW peptide recognition site consisting of R17, Y23 and W34 while the AIE-core and linker interact with R14, M15 and F25. (b) Face-to-edge π–π-stacking of hPin1-WW residue F25 with the central and distal aromatic AIE rings (F25 and AIE-core shown as space-fill). This tight interaction of the aromatic rings restricts the AIE intramolecular rotations and thus promotes the observed increase in fluorescence upon protein binding. (c) Interaction of hPin1-WW residues R14 and M15 with F_1_1. M15 interacts with the triazole unit. R14 is found in two conformations, engaging in cation–π interactions with the outside (left) or inside (right) of the C-shaped AIE-core.

The overall saddle-shaped fold of hPin1-WW with its central three-stranded β-sheet does not differ significantly from the apo structure^22–24^ or the structures with bound native peptides^24–26^ (Fig. 4 and S10). Generally, the conserved Y23 and W34 constitute the proline binding site.^24–26^ The side chain of W34 and the phosphate-binding loop adjust position to accommodate different substrate sequences flanking the pThr-Pro binding motif. The AIE-peptide F_1_1 is draped over the saddle-shaped top of the central hPin1-WW β-sheet and no longer in a curled-up conformation; however, the first peptide bond remains in *cis* conformation. The peptide arm is extended, with the pThr-Pro motif bound on top of the β-sheet and in close contact with the phosphate-binding loop. The Pro residue is recognized in the Xaa-Pro binding groove of the WW domain, where it forms CH–π interactions with residues Y23 and W34.

The side chain of residue R17 is described as the additional recognition residue for phosphorylated substrates^20^ and is in close proximity to the pThr phosphate group, likely stabilized by a salt bridge. However, R17 is not assigned in the NMR spectra because its signal is absent in ^15^N-HSQC. Therefore, no NOE information is available for this residue and its position is not well-defined in the structure.

The AIE-core remains C-shaped but now lies on top of the β-sheet surface, forming contacts with the protein through its aromatic rings, contributing the largest buried surface area (BSA) per residue of 314.9 Å^2^ to the interface (Table S5). Residue F25 of hPin1-WW rotates into the centre of the C-shape, forming face-to-edge π–π interactions with the central and distal aromatic rings of the AIE-core (Fig. 4b). This tight fit limits the rotation of the AIE rings, which explains the increase in AIE fluorescence upon hPin1-WW binding. A similar involvement of residue F25 was only seen for the Smad3 peptide (IPEpTPPPG, Fig. S10c),^25^ where the first proline of the peptide engages in a CH–π interaction with the F25 phenyl ring. Furthermore, the glutamate residue of the Smad3 peptide forms a salt bridge with R14 of hPin1-WW. Residue R14 is also involved in stabilizing the aromatic AIE-core of F_1_1 by cation–π interactions. R14 is found in two conformations: either R14 is placed in the centre of the aromatic C-shape or at the outside of the proximal aromatic ring of the AIE-core. Residue M15 displays a conformation that is unique among all known hPin1-WW structures (apo and peptide-bound). Usually, the M15 side chain points downwards, away from the peptide-binding face. Here, in contrast, the M15 side chain is rotated upwards, pointing up to the triazole ring of the fluorophore (Fig. 4c). Methionine–aromatic interactions were first suggested by bioinformatics studies^37,38^ and were shown to stabilize protein folding.^39–41^ The M15 sulphur is in close proximity to the triazole ring and proximal phenylene ring, potentially engaging in a similar interaction and thus adding an additional contact to the protein–fluorophore interface. This contact is supported by several intermolecular NOEs between the M15 methyl group and the F_1_1 aromatic protons and adjacent methylene group.

Overall, our structure shows how the AIE-core lies flush against the protein surface, held in place by several weak interactions between its aromatic rings and protein surface residues, esp. F25, R14 and M15. This close contact restricts aromatic ring movement, which results in the fluorescence turn-on signal observed in the fluorescence binding assay.

## Conclusions

In this work, we coupled AIE fluorophores with peptides targeting the hPin1-WW domain as a model system for the quantitative characterisation of protein binding *via* direct read-out, named aggregation-induced emission enhancement (AIEE), and present the first experimental structures of an AIE ligand bound to a protein.

Coupling of peptides to the AIE-core *via* click chemistry offers a convenient system to pair the AIE properties with a multitude of peptides to target a variety of peptide-binding proteins.

AIEE has previously been shown for AIEs equipped with molecular tweezers to target the proteins p97^17^ and Taspase1.^16^ Molecular modelling of these systems suggested contacts between the AIE-cores and the protein surfaces. Here, we compare the solution structures of a free AIE-peptide and its complex bound to hPin1-WW. The free AIE-peptide adopts a curled-up structure stabilized by intramolecular CH–π contacts between the proline and the aromatic AIE-core. Despite the compact structure, the free rotation of at least one aromatic ring of the AIE-core is possible and accounts for the low auto-fluorescence.

The ligand binds in a more extended conformation, which allows the recognition of the primary peptide binding motif by the proline-binding pocket and the phosphate-binding loop of hPin1-WW. In addition, the AIE-core nestles up against the protein surface. The specific contacts of the AIE-core with the protein surface limit its intramolecular movement, especially rotation, which explains the observed fluorescence increase upon protein binding.

Interestingly, the binding affinity does not improve but rather the *K*_D_ slightly increases for the AIE-peptides compared to the native control peptide P0, despite the additional contacts between the AIE-core and the protein. Most likely, this can be attributed to basically all intramolecular contacts that hold up the compact structure of the free AIE-peptide needing to be broken, and the AIE-peptide needs to stretch out fully to allow protein binding to its peptide recognition site and allow the AIE-core to drape over the protein surface. The movement restriction in the bound state likely results in a loss of entropy. Furthermore, it is possible that the peptide experiences a slightly different conformation upon binding due to the presence of the AIE fluorophore, especially at its N-terminus where the control peptide carries a native Ala residue. Since the AIE-peptides F_2_5 and F_2_6 with non-binding peptide sequences showed high aggregation and thus autofluorescence in the protein binding buffer and the AIE-cores prior to peptide attachment are insoluble in aqueous buffers, we cannot quantify the contribution of the AIE-core to the hPin1-WW binding in isolation.

Generally, AIEE requires interaction of the AIE fluorophore with a hydrophobic patch on the protein surface. We and others have successfully observed AIEE using AIE fluorophores conjugated to other protein-recognizing moieties, like supramolecular tweezers or GCP ligands, with multiple different proteins.^5,8,16,17,42–46^ Computational modelling and MD simulations of these systems also support interactions of the AIE moieties with the respective protein surface. Thus, AIEE is expected to work for many globular proteins. A uniformly highly charged protein surface with little to no hydrophobic patches is likely not conducive to AIE binding. However, most globular, folded protein domains do not present such a highly charged surface because high internal electrostatic repulsion destabilizes a compact folded structure.^47,48^ Highly charged protein regions therefore rarely adopt a global fold and mostly occur in intrinsically disordered proteins (IDPs) or domains.^47,48^ Hydrophobic surface patches occur frequently in protein interaction interfaces on globular domains^49^ and thus are accessible to AIE binding.

Each protein:AIE-peptide system will require optimization of the linker length between AIE and peptide recognition sequence to allow the AIE to reach its ideal spot on the protein surface. The fusion of an AIE with a specific protein binding motif, such as a peptide recognition sequence, turns binding of the preorganised AIE into an intramolecular event, which is favoured if the linker length is in the optimum range.^50^ If the linker is too short, binding of the AIE might be hindered. If the linker is too long, the advantage of intramolecular binding will likely dissipate, leading to no more AIE binding and hence not enough motion restriction of the AIE fluorophore. Short linkers, the presence of charged residues and lower fluorophore concentrations (25–50 µM) are advantageous to obtain reliable binding data. Varying the spacer length between the AIE-core and the protein recognition site did not significantly alter binding affinity or fluorescence increase upon hPin1-WW binding; however, this distance might need to be optimized when targeting other proteins.

The interaction of the AIE with the protein does influence the binding behaviour, and thus, it can lead to noticeably stronger or weaker binding and a change in binding kinetics, depending on the system studied. Therefore, the AIE-peptides provide a useful tool to determine the order of magnitude for the binding affinity and should be complemented by orthogonal, ideally label-free, binding assays.

Since the AIE-core is hydrophobic and solubility can be a challenge, AIE-peptide sequence needs to be optimized and tested for possible aggregation, which can be gauged by their autofluorescence intensity at different concentrations.

The two-armed AIE-peptides F_2_1–F_2_4 showed a higher emission intensity and amplitude and are thus more suitable for fluorescence binding assays. However, changing the trimethyl ammonium end group of the one-armed AIE-peptide F_1_1 might improve its emission intensity.

The biggest advantage of AIE is the unique binary fluorescence “on” behaviour upon complexation, providing a direct readout of the successful interaction between host and guest, which cannot be achieved with classic luminophores. Just like classical fluorophores, AIE fluorophores are available for many wavelengths.^10,11,28,51,52^

Classical luminophores (*e.g.* fluorescein, Alexa-, Atto- or Cy-dyes) shine through their wide availability, good water solubility, large colour selection in the visible range, as well as excellent quantum yield, which allows usage in nM concentrations. The widely used fluorescence polarization techniques based on classical dyes rely on the increase of the rotational correlation time upon complex formation and generally work best when the smaller component is labelled to maximize the size increase upon complex formation. However, in our experience, this rule of thumb does not always hold true and the change in anisotropy can be smaller or larger than expected, especially when the shape of the complex changes in addition to size. Energy transfer of the fluorophore, *e.g.* quenching upon binding, or homo-fluorescence FRET due to multiple fluorophores being attached to one molecule or assembly^53^ can also interfere with data analysis. AIEE is independent of molecule size and shape and has been shown to work for small systems, such as hPin1-WW presented here, up to large assemblies like the p97 hexamer.^17^

Quenching of the Trp fluorescence of proteins^54^ upon ligand binding is also widely used to determine binding constants.^55,56^ The intrinsic nature of Trp residues avoids the need for a synthetic label, and Trp fluorescence is very responsive to changes in the environment, albeit the fluorescence in the UV range and lower quantum yield compared to classical fluorophores can pose a challenge (see below). The environmental sensitivity can also be a curse, since quenching can be caused by a change in buffer conditions (*e.g.* pH or salt concentration). For example, the Trp fluorescence binding curve of hPin1-WW with the control peptide P0 (Fig. S8b and Table 1) shows significantly larger error bars than the AIE-peptide titrations or the anisotropy titration with FAM-P0 (Fig. S8a and Table 1). Furthermore, this technique is not applicable for proteins without Trp, and introducing one by site-directed mutagenesis can interfere with protein folding. For optimal responsiveness, Trp should be located in the vicinity of the binding interface, like in hPin1-WW. Too many Trp cause a high fluorescence background. In addition, the titrated ligand must not have Trp residues itself. Due to the UV fluorescence of Trp, aromatic ligands can cause an inner-filter effect, even in the absence of auto-fluorescence, which can complicate data analysis; however, shifting the excitation wavelength to 295 nm can attenuate this effect.^56^ The AIEE fluorescence “on” behaviour is less dependent on protein and ligand composition and dominated by the internal molecular motions and thus circumvents many of the challenges of Trp fluorescence quenching.

Overall, the AIE-peptides are a useful addition to the toolbox of fluorescence-based methods, shining through their unique fluorescence “on” behaviour upon protein binding, provided they do not form soluble aggregates.

AIE-peptides can be used for the quantitative characterisation of protein binding using direct fluorescence read-out, whereby the necessary AIE:protein interaction can affect the effective binding constant. Combination of peptides with different AIE cores with up to 4 “arms” will offer the possibility to design multivalent peptidic supramolecular ligands for multimeric protein assemblies, where the AIE core is designed to add to the protein–ligand interaction.

## Author contributions

Conceptualisation and supervision: P. B., J. V., and C. B.; investigation, data curation and analysis: D. S., C. B., M. H., D. P., J. R., and J. L.; software and data analysis: T. H.; visualisation: D. S. and C. B.; funding acquisition: P. B., J. V., and M. K.; writing – original draft: C. B., J. V., and D. S.; writing – review & editing: C. B., J. V., and P. B.

## Conflicts of interest

There are no conflicts to declare.

## Supplementary Material

CB-007-D6CB00109B-s001

## Data Availability

The atomic coordinates for the F_1_1 (DS-TPO-PRO-ALA-NH2) and the F_1_1:hPpin1-WW complex structures have been deposited in the Protein Data Bank (PDB) under accession numbers 29KP (extended pdb ID: pdb_000029KP) and 29KQ (extended pdb ID: pdb_000029KQ), respectively. NMR data have been deposited in the Biological Magnetic Resonance Data Bank (BMRB) under accession numbers 35045 and 35046. The supporting data have been provided as part of the supplementary information (SI). Supplementary information: supporting figures and tables, structure statistics, compound synthesis and characterization, as well as detailed and complete experimental procedures. See DOI: https://doi.org/10.1039/d6cb00109b.
