# Supplementary material for: Solution structure of a peptide-conjugated aggregation-induced-emission (AIE) fluorophore bound to a protein
Source: RSC Chem Biol. 2026 Oct 15;7(11):2299–308. doi: 10.1039/d6cb00109b (PMC13670144; doi:10.1039/d6cb00109b)
Supplement: Supplementary file 1 [file CB-007-D6CB00109B-s001.pdf]

Supplementary Information (SI) for RSC Chemical Biology.
This journal is © The Royal Society of Chemistry

Supplemental Information

Solution Structure of a Peptide-Conjugated Aggregation-Induced-Emission (AIE) Fluorophore Bound to Protein

Daniel Seifert[a], Matthias Hayduk[b], Torsten Herrmann[c], David Podlesainski[d], Jill Rohde, Jürgen Linders[e], Markus Kaiser[d], Peter Bayer[a], Jens Voskuhl[b*] and Christine Beuck[a*]

Contents

Chemical Synthesis 2
AIE-core synthesis 2
Peptide synthesis 2
Copper-catalysed azide-alkyne cycloaddition (Click reaction) 3
L-Homoalanine-O-Phospho-L-Threonine-L-Proline-L-Alanine-Amide conjugated *para*-phenol-thioterephthalonitrile (DS-TPO-PRO-ALA-NH2; F_1**1**) 5
Di-(L-Homoalanine-O-Phospho-L-Threonine-L-Proline-L-Alanine-Amide)- conjugated para-phenolthioterephthalonitrile (F_2**1**) 7
Di-(L-Homoalanine-L-Alanine-O-Phospho-L-Threonine-L-Proline-L-AlanineAmide)-conjugated *para*-phenolthioterephthalonitrile (F_2**2**) 9
Di-(L-Homoalanine-L-Alanine-Glycin-O-Phospho-L-Threonine-L-Proline-L-Alanine-Amide)-conjugated *para*-phenolthioterephthalonitrile (F_2**3**) 11
Di-(L-Homoalanine-L-Alanine-Glycin-L-Alanine-Glycin-O-Phospho-L-ThreonineL-Proline-L-Alanine-Amide)-conjugated *para*-phenolthioterephthalonitrile (F_2**4**) 13
Di-(L-Homoalanine-L-Threonine-L-Proline-L-Alanine-Amide)-conjugated paraphenolthioterephthalonitrile (F_2**5**) 15
Di-(L-Homoalanine-L-Threonine-Glycine-L-Alanine-Amide)-conjugated paraphenolthioterephthalonitrile (F_2**6**) 17
Methods 19
Preparative HPLC 19
Protein Expression and Purification. 19
Mass spectrometry 19
UV-Vis spectroscopy 19
Fluorescence spectroscopy 20
NMR spectroscopy 21
NMR structure calculations 22
Supplemental Tables 23
Supplemental Figures 27
Non-standard Residue Parameter Files (CYANA Library Files) 35
References 38

Chemical Synthesis

AIE-core synthesis

The alkyne functionalized AIE luminophores **A_1** and **A_2** (Fig. S0-1) have been described beforehand by us and can be synthesised in short linear synthetic routes.[1,2]

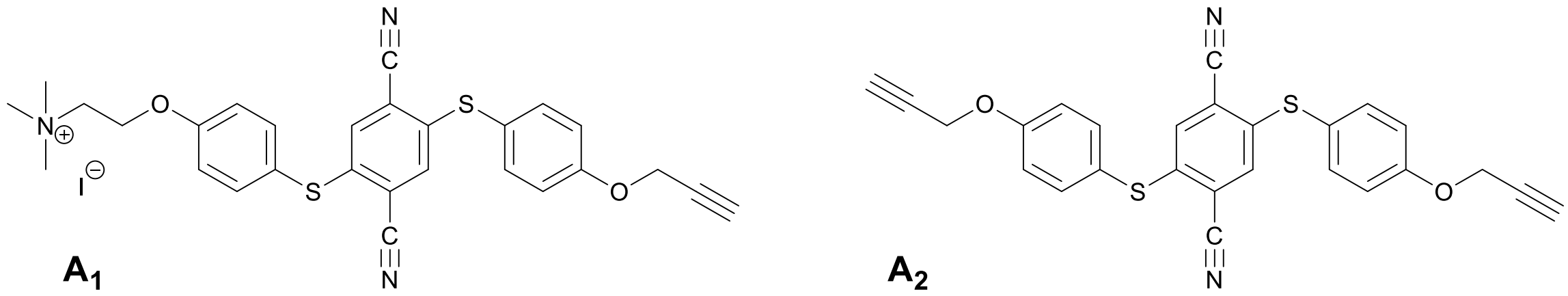

A_1 **A_2**

Figure S0-1: AIE-cores **A_1** and **A_2** containing one or two terminal alkynes for functionalization via Click chemistry.

Peptide synthesis

All peptides with azido-homoalanine were synthesised using standard solid phase peptide synthesis (SPPS) based on Fmoc chemistry using DIC and HBTU as coupling reagents.

200mg of Rink amide resin was swollen in DCM and washed twice with DMF. The FMOC group was cleaved by incubation with 40% piperidine in DMF for 3 min, followed by a second deprotection step with 20% piperidine in DMF for 12 min and washing with DMF (6x).

Amino acids were dissolved with 0.1M HOBt in DMF. For amino acid coupling, the resin was incubated with the amino acid solution (4 eq), HBTU (4 eq) in DMF and DIPEA (4 eq) in NMP (final volume: 2.04 mL) while shaking for 40 min. The resin was washed with DMF (3x) and the coupling step was repeated with DIC (4 eq) in DMF instead of HBTU. The coupling procedure was repeated for all amino acids until the peptide sequence was complete.

The finished peptide was cleaved from the resin and deprotected by incubating the resin with TFA (95%), TIPS (2.5%) and H_2O (2.5%) for 3 h. The cleaved peptides in solution were precipitated in 45 mL ice cold diethyl ether and isolated by centrifugation. The peptides were then dissolved in 20% CH_3CN/80% H_2O and purified by reverse phase (rp) HPLC, yielding the pure peptides as white solids after lyophilization. Peptide sequences and yields are summarized in Table S0-1.

The control peptides **P0** and **FAM-P0** without AIE fluorophore were purchased from CASLO (Denmark).

Table S0-1: Peptide sequences and yields.

Peptide	Sequence*	Yield
**P1**	Aha-pThr-Pro-Ala-NH_2	65.3 mg (132.6 mmol, 94%)
**P2**	Aha-Ala-pThr-Pro-Ala-NH_2	77.0 mg (136.6 mmol, 98%)
**P3**	Aha-Ala-Gly-pThr-Pro-Ala-NH_2	82.5 mg (132.9 mmol, 92.9%)
**P4**	Aha-Ala-Gly-Ala-Gly-pThr-Pro-Ala-NH_2	103.1 mg (137.6 mmol, 88%)
**P5**	Aha-Thr-Pro-Ala-NH_2	69.6 mg (168.7 mmol, 99.2%)
**P6**	Aha-Thr-Gly-Ala-NH_2	49.2 mg (131.6 mmol, 94.0%)
**P0**	Ala-pThr-Pro-Ala-NH_2	n.a.**
**FAM-P0**	5,6-FAM-Ala-pThr-Pro-Ala-NH_2	n.a.**

* Aha = L-azido-homoalanine; pThr = L-phosphothreonine; NH_2 = amidated C-terminus

** n.a. = not applicable - -peptide was purchased from Caslo (Denmark).

LC-MS (ESI): $[2M+H]^+$ and $[3M+H]^+$ are Coulomb dimers and trimers, commonly observed for peptides in the ESI-MS.

P1: calculated $C_{16}H_{29}N_8O_8P$, M = 492.43. t_R 1.69 min; *m/z* 492.95 ($[M+H]^+$), 984.82 ($[2M+H]^+$), 1476.72 ($[3M+H]^+$).

P2: calculated $C_{19}H_{34}N_9O_9P$, M = 563.51. t_R 2.12 min; *m/z* 565.08 ($[M+H]^+$), 1128.91 ($[2M+H]^+$), 1693.08 ($[3M+H]^+$).

P3: calculated $C_{21}H_{37}N_{10}O_{10}P$, M = 620.56. t_R 1.76 min; *m/z* 621.09 ($[M+H]^+$), 1240.77 ($[2M+H]^+$), 1860.48 ($[3M+H]^+$).

P4: calculated $C_{26}H_{45}N_{12}O_{12}P$, M = 748.69. t_R 1.82 min; *m/z* 375.17 ($[M+2H]^{2+}$), 749.22 ($[M+H]^+$), 1497.08 ($[2M+H]^+$).

P5: calculated for $C_{16}H_{28}N_8O_5$, M = 412.45. t_R 2.03 min; *m/z* 413.94 ($[M+H]^+$), 826.68 ($[2M+H]^+$).

P6: calculated for $C_{13}H_{24}N_8O_5$, M = 372.39. t_R 1.91 min; *m/z* 373.97 ($[M+H]^+$), 746.67 ($[2M+H]^+$).

Copper-catalysed azide-alkyne cycloaddition (Click reaction)

Azido-homoalanine-containing peptides **P1-P6** were coupled to the AIE-active core (**A₁** or **A₂**) by a copper(I) catalysed azide-alkyne cycloaddition[3,4] (Click reaction, Fig. S0-2).

Dry AIE-active core (8-10 mg), the peptide (2.4 eq), $CuSO_4$ (0.6 eq) and Na-ascorbate (1.3 eq) were mixed, degassed, dissolved in degassed THF/H_2O (2:1, 9 mL) and mixed at RT for 3-5 days under argon atmosphere. The peptide-coupled AIE fluorophores were purified by preparative rp-HPLC and lyophilized to obtain yellow solids.

To exchange trifluoroacetate counterions for chloride, the lyophilized peptide-conjugated fluorophores were dissolved in 100 mM aqueous HCl, incubated for 1 min at RT and subsequently lyophilized. This process was repeated 3 times. To remove excess HCl the fluorophores were washed with H_2O and lyophilized twice. The absence of TFA was confirmed by ^{19}F-NMR spectroscopy.

Yields, the respective AIE-core and coupled peptide are summarized in Table S0-2.

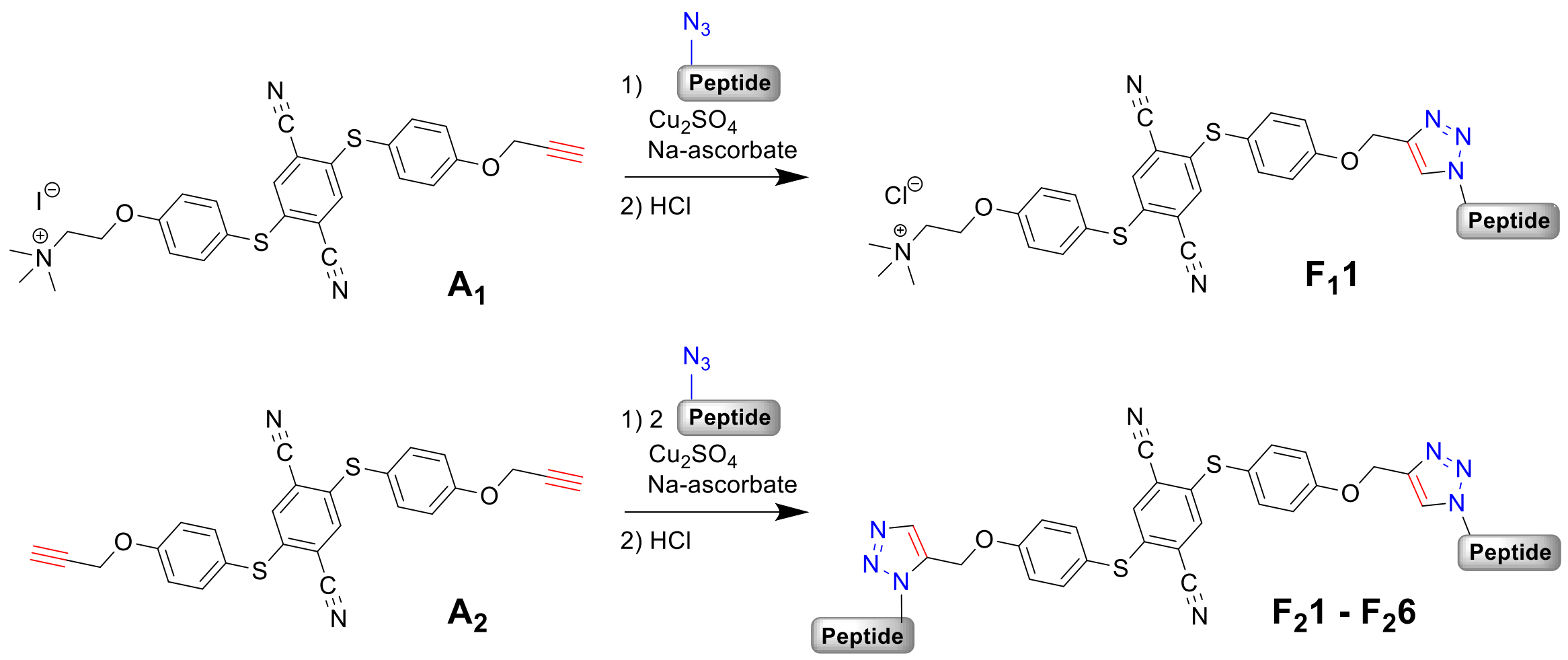

Figure S0-2: Synthesis of AIE-peptide fluorophores via Copper-catalysed azide-alkyne cycloaddition (Click reaction). The azide nitrogen atoms (blue) and alkyne carbon atoms (red) that form the triazole ring after the Click reaction are coloured. Each AIE-core/peptide combination for the synthesised peptide-coupled AIE fluorophores are listed in Table S0-2.

Table S0-2: AIE-peptide fluorophores with their yields as well as corresponding AIE-core and peptide.

AIE-Peptide	AIE-core	Peptide	Yield
**$F_1$1**	**A_1**	**P1**	9.6 mg (9.7 mmol, 49.7%)
**$F_2$1**	**A_2**	**P1**	12.8 mg (8.9 mmol, 49.6%)
**$F_2$2**	**A_2**	**P2**	8.3 mg (5.3 mmol, 32.4%)
**$F_2$3**	**A_2**	**P3**	22.8 mg (13.5 mmol, 55.3%)
**$F_2$4**	**A_2**	**P4**	13.7 mg (7.0 mmol, 54.8%)
**$F_2$5**	**A_2**	**P5**	17.1 mg (13.4 mmol, 50.0%)
**$F_2$6**	**A_2**	**P6**	5.5 mg (4.6 mmol, 18.9%)

L-Homoalanine-O-Phospho-L-Threonine-L-Proline-L-Alanine-Amide conjugated *para*-phenol-thioterephthalonitrile (DS-TPO-PRO-ALA-NH2; $F_1$1)

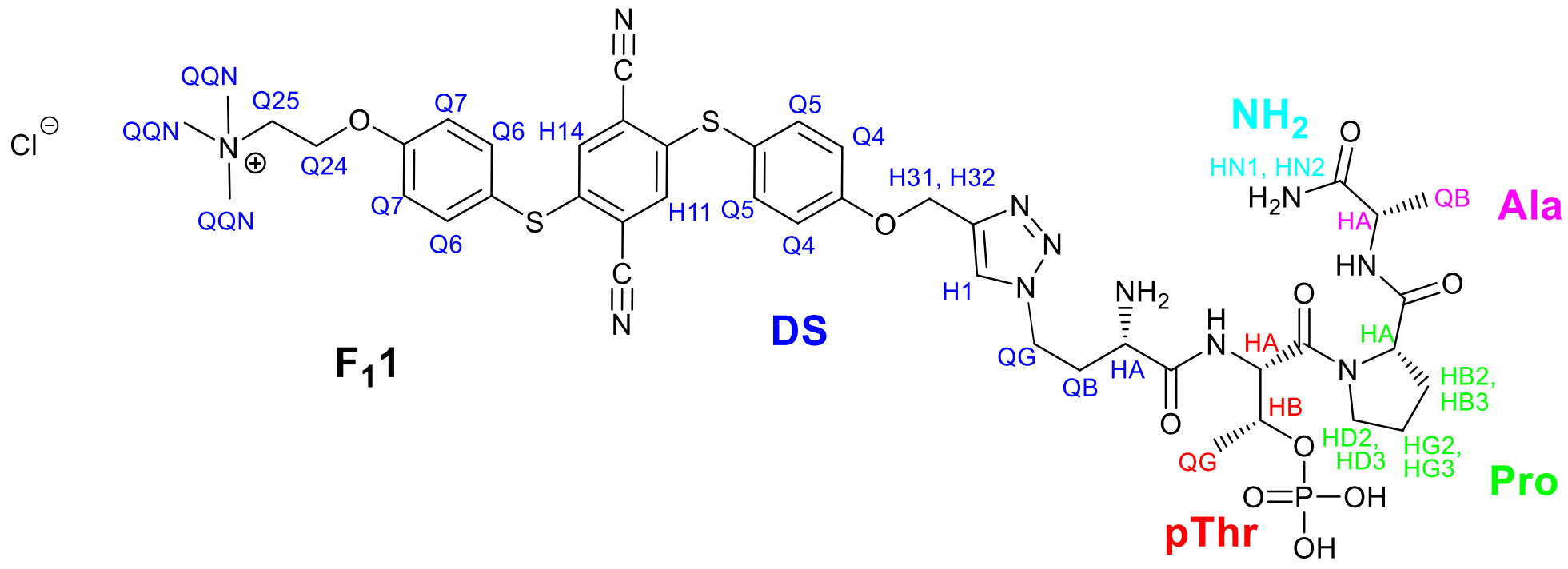

^{1}H NMR (700 MHz, 10% D_2O/ 90% H_2O): ^{1}H atom and residue nomenclature correspond to the nomenclature and parametrization used for the structure calculation in Cyana. The residue DS comprises the fluorophore-conjugated homoalanine including the triazole linker. The peptidyl-prolyl amide bond can adopt a *trans* or *cis* conformation. While the main conformer used for the structure calculation shows the DS-pThr peptide bond in *cis* conformation, it is possible that this bond could adopt the *trans* conformation in the minor conformer. Separate signals of the minor *cis*/*trans* conformer (~20%) are marked with *. Integrals are given within the major or minor conformer, respectively. For the NMR structure calculation, only the chemical shifts for the most abundant conformer (DS-pThr peptide bond *cis*, pThr-Pro bond *trans*) were used.

δ [ppm] 8.97 (d, J=6.4 Hz, 1H, HN, pThr), 8.71 (d, J = 8.9 Hz, 1H, HN, pThr*), 8.59 (d, J 5.4 Hz, 1H, HN, Ala*), 8.39 (d, J = 6.1 Hz, 2H, HN, Ala), 8.13 (s, 1H, H_{aro}, DS*), 8.12 (s, 1H, H1 (H_{aro}), DS*), 7.51 (s, 1H,HN1 NH_2), 7.49 (s, 1H, HN1, HN_2*), 7.45 (d, J = 8.9 Hz, 2H, Q6 (H_{aro}), DS), 7.43 (d, J = 8.9 Hz, 2H, Q5 (H_{aro}), DS), 7.28 (s, 1H, H14 (H_{aro}), DS), 7.26 (s, 1H, H11 (H_{aro}), DS), 7.07 (d, J = 9.4 Hz, 2H, Q7 (H_{aro}), DS), 7.06 (d, J = 10.7 Hz, 2H, Q4 (H_{aro}), DS), 6.96 (s, 1H, HN2, NH_2), 6.90 (s, 1H, HN2, HN_2*), 5.20 (s, 1H, H3B, DS), 5.18 (s, 1H, H3A (CH_2), DS), 4.60 (1H, HA (CH), pThr*), 4.57 (d, J = 8.4 Hz, 1H, HA (CH), DS), 4.53-4.50 (m, 3H, HB (CH), pThr + Q24 (CH_2), DS), 4.40-4.38 (m, 1H, HA (CH), Pro + HB (CH), pThr*), 4.24 (m, 1H, HA (CH), Ala), 4.19-4.16 (m, 2H, QG (CH_2), DS + HA (CH), Ala*), 3.86-3.82 (m, 3H, HD3 (CH_2), Pro + Q25 (CH_2), DS), 3.77-3.74 (m, 1H, HD2 (CH_2), Pro), 3.52-3.47 (m, 2H, QD Pro*), 3.25 (s, 9H, QQN (NMe_3), DS), 2.53 (dd, J = 7.0 Hz, 2H, QB (CH_2), DS), 2.44 (m, 1H, HB3, Pro*), 2.28-2.24 (m, 1H, HB3 (CH_2), Pro), 2.14-2.10 (m, 1H, HB2, Pro*), 2.04-2.00 (m, 1H, HG3 (CH_2), Pro), 1.95-1.86 (m, 2H, HG2 (CH_2), Pro + HB2, Pro), 1.88 (m, 1H, HG3, Pro*), 1.77 (m, 1H, HG2, Pro*),1.44 (d, J = 7.5 Hz, 3H, QB (CH_3), Ala*), 1.37 (d, J = 6.2 Hz, 3H, QG (CH_3), pThr), 1.34 (d, J = 7.3 Hz, 3H, QB (CH_3), Ala), 1.28 (d, J = 6.0 Hz, 3H, QG (CH_3), pThr*).

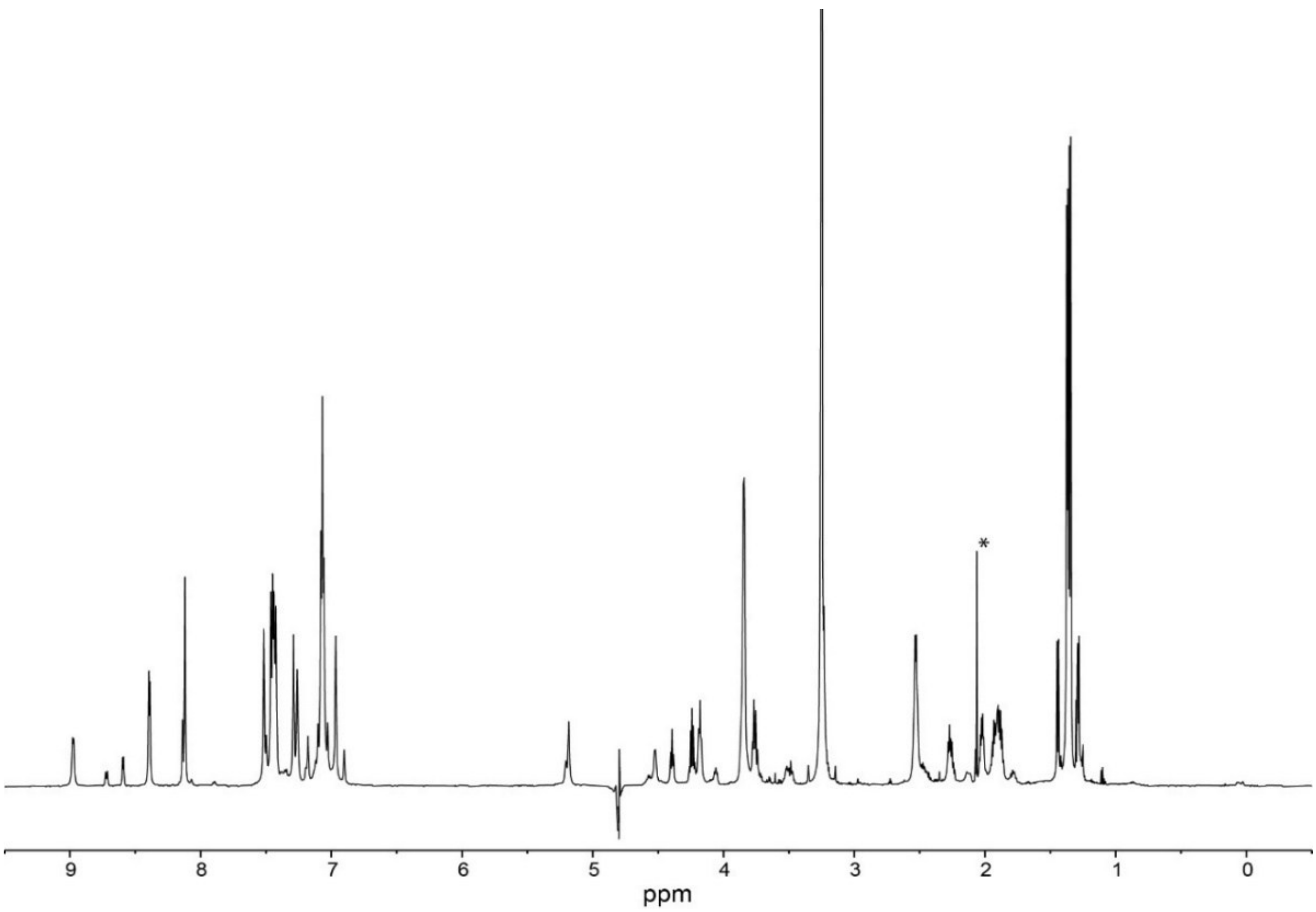

Figure S0-15. ^{1}H NMR spectrum (700 MHz, D_2O) of **$F_1$1**. * marks impurities.

LC-MS (ESI): calculated for $C_{44}H_{55}N_{11}O_{10}PS_2^+$: 993.08. t_R= 6.01 min; *m/z*= 496.76 $[M + H]^{2+}$, 992.11 $[M]^+$.

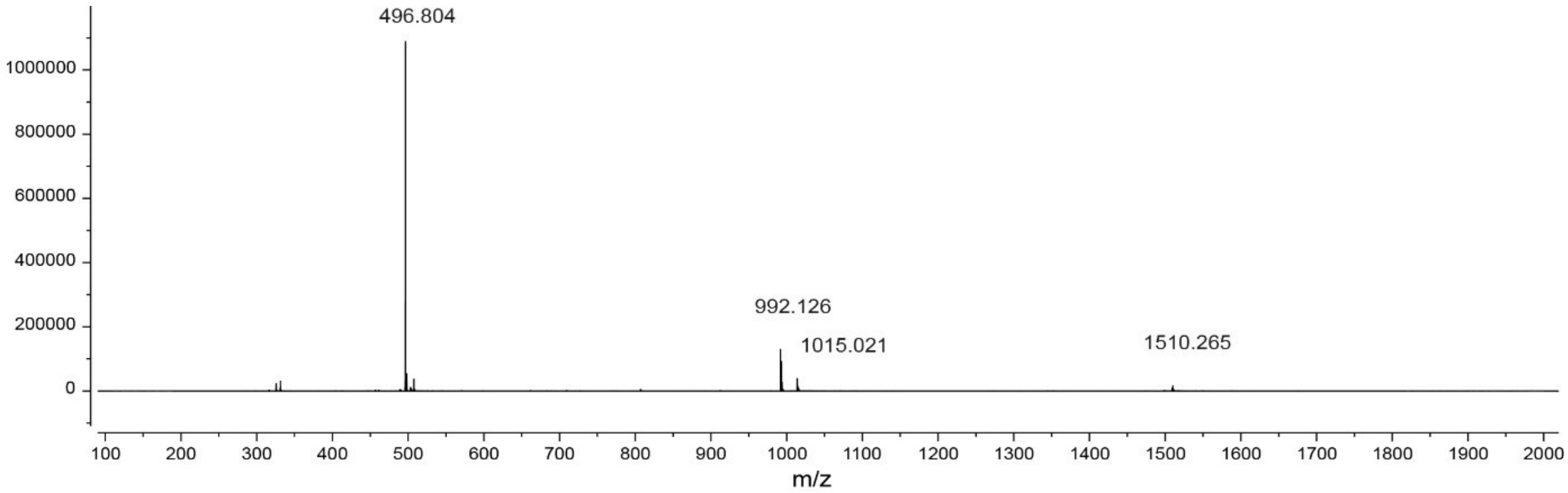

Figure S0-16. LC-MS of **$F_1$1**.

HR-MS:

Ion	*m/z* calculated	*m/z* measured	Δ [ppm]
$[M]^+$	992.3307	992.3278	2.92
$[M]^+$	993.3340	993.3321	1.91
$[M]^+$	994.3374	994.3335	3.92
$[M+H]^{2+}$	496.6690	496.6676	2.82
$[M+H]^{2+}$	497.1707	497.1689	3.62
$[M+H]^{2+}$	497.6723	497.6702	4.22

Di-(L-Homoalanine-O-Phospho-L-Threonine-L-Proline-L-Alanine-Amide)- conjugated para-phenolthioterephthalonitrile (F_21)

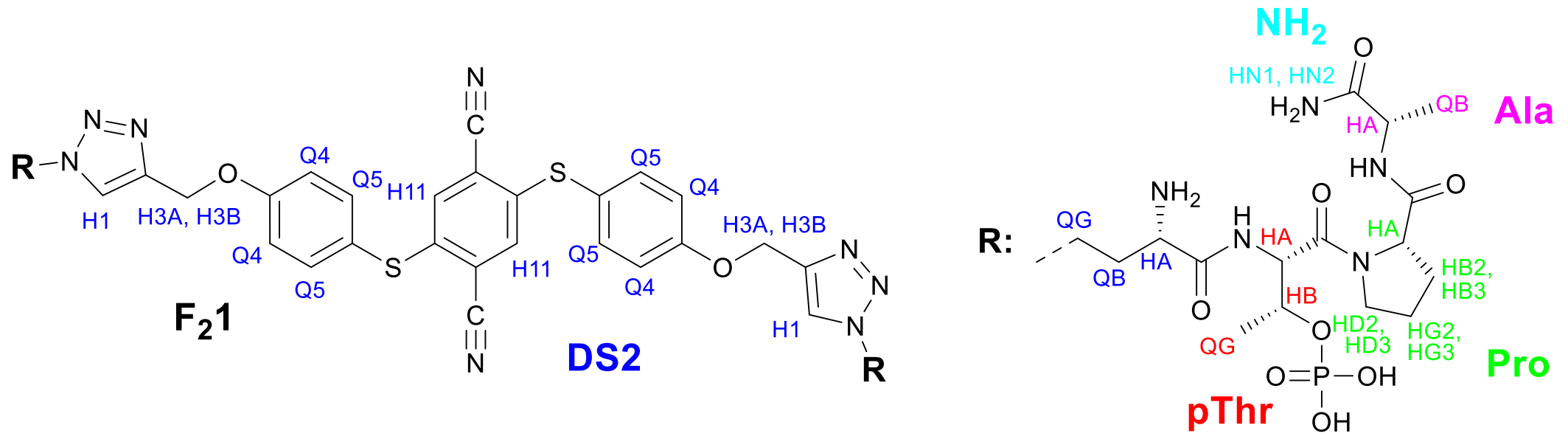

^{1}H NMR (700 MHz, 10% D_2O/ 90% H_2O): The peptidyl-prolyl and/or DS-pThr amide bonds can adopt a *trans* or *cis* conformation, separate signals of the minor conformation are marked with *. Integrals are given within the major or minor conformer, respectively. The DS2 residue comprises the fluorophore (H_{aro}), the triazole linker and the two homoalanines.

δ [ppm] 8.95 (br. s, 2H, HN, pThr), 8.73 (br. s, 2H, HN pThr*), 8.51 (br. s, 2H, HN, Ala*), 8.27 (br. s, 2H, HN, Ala), 8.07 (br. s, 2H, H1 (H_{aro}), DS2*), 8.01 (br. s, 2H, H1 (H_{aro}), DS2), 7.48 (br. s, 2H, HN1, NH_2), 7.26 (br. s, 8H, Q5 (H_{aro}), DS2), 7.01 (br. s, 4H, H11 (H_{aro}), DS2), 6.98 (br. s, 2H, HN2, NH_2), 6.87 (br. s, 8H, Q4 (H_{aro}), DS2), 4.59 (br. s, 2H, HA, pThr*), 4.78 (br. s, 2H, HA, pThr), 4.54-4.52 (m, 4H, HA, DS2 + HB, pThr), 4.34 (br. s, HA, Pro), 4.38 (br. s, 2H, HB, pThr*), 4.23 (br. s, 4H, QG, DS2), 4.19 (m, HA, Ala + HA, Ala*), 4.23 (br. s, 4H, QG, DS2*), 3.78 (br. s, 2H, HD3, Pro), 3.72 (br. s, 2H, HD2, Pro), 3.44 (m, 2H, HD3, Pro*), 3.39 (m, 2H, HD2, Pro*), 2.50 (br. s, QB, DS2 + QB, DS2*), 1.94 (br. s, 2H, HG3, Pro), 1.82 (br. s, 2H, HG2, Pro), 1.40 (d, J = 7.4 Hz, 6H, QB (CH_3), Ala*), 1.34 (d, J = 5.2 Hz, 6H, QG (CH_3), pThr), 1.27 (d, J = 6.4 Hz, 6H, QB (CH_3), Ala), 1.23 (d, J = 5.6 Hz, 6H, QC (CH_3), pThr*).

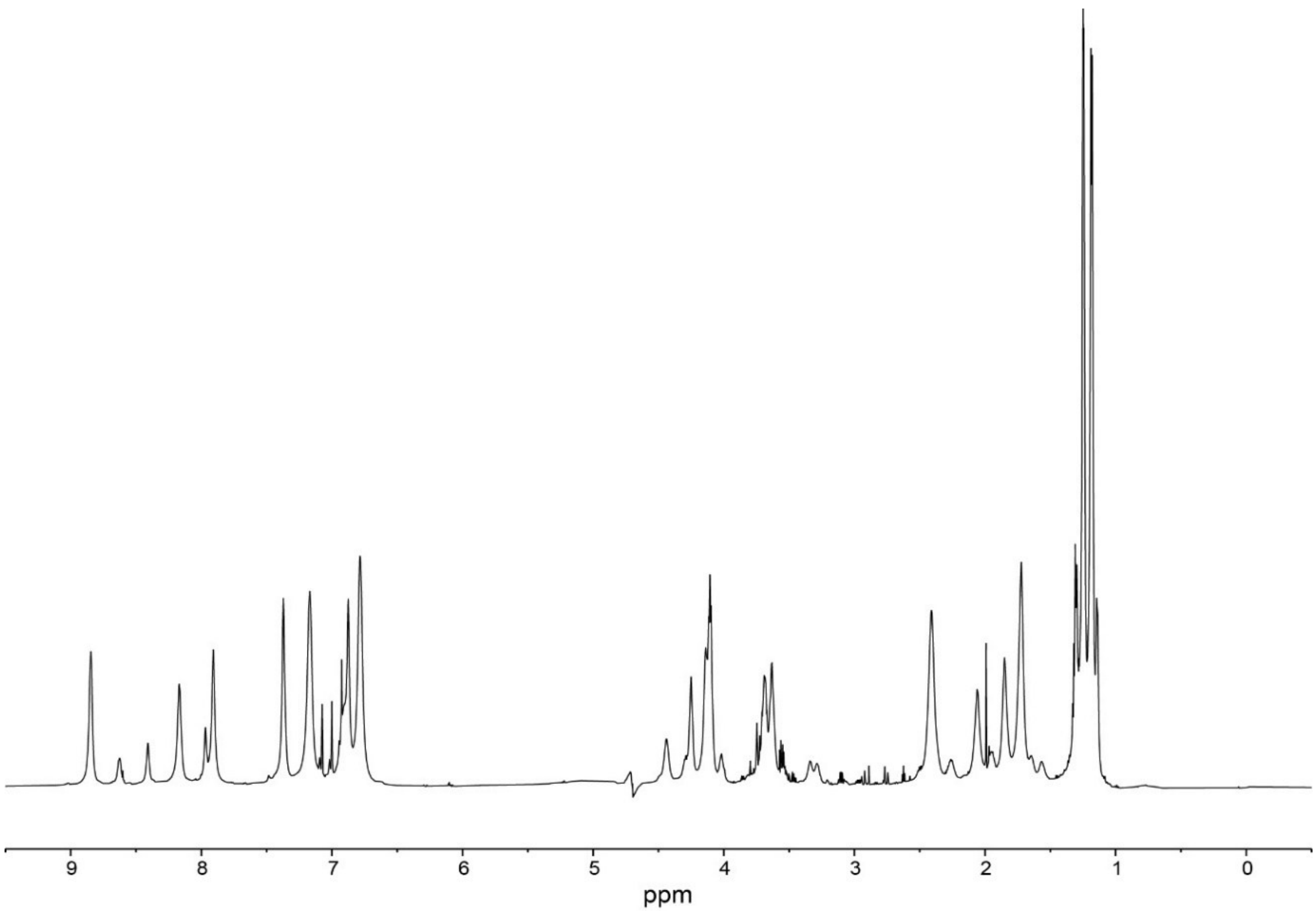

Figure S0-3. ^{1}H NMR spectrum (700 MHz, D_2O) of **F_21**.

LC-MS (ESI): calculated $C_{58}H_{74}N_{18}O_{18}P_2S_2$, M = 1437.41. t_R= 6.00 min; *m/z*= 479.96 $[M + 3H]^{3+}$, 719.52 $[M + 2H]^{2+}$, 1437.14 $[M + H]^{+}$.

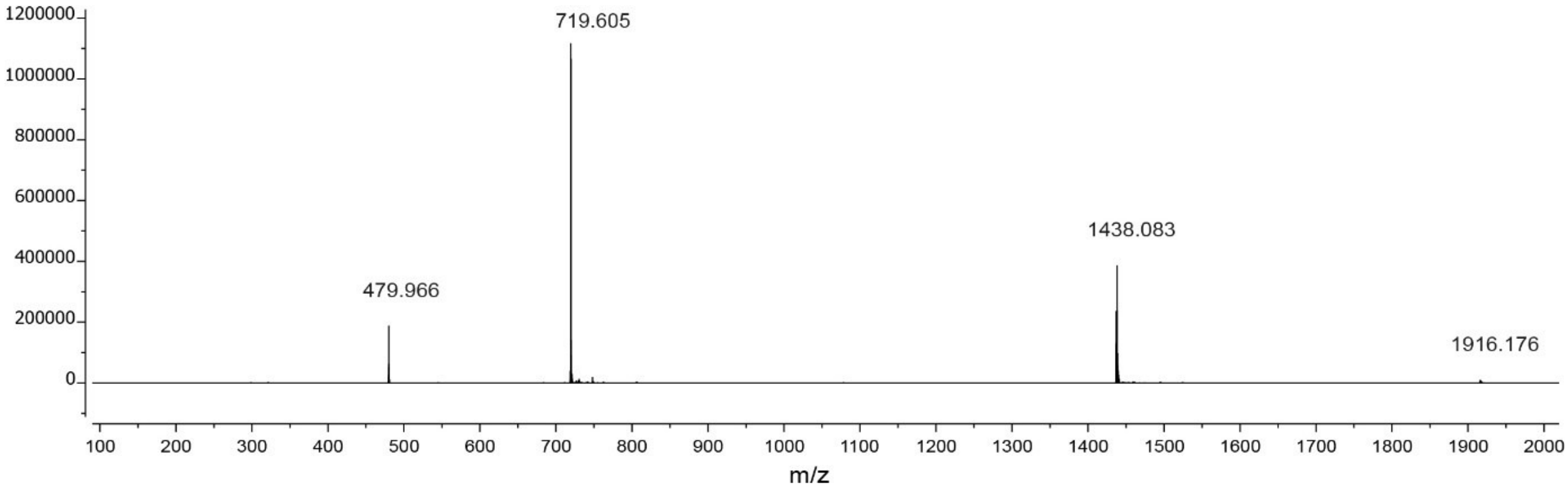

Figure S0-4. LC-MS of $\mathbf{F_2 1}$.

HR-MS:

Ion	*m/z* calculated	*m/z* measured	Δ [ppm]
$[M+2H]^{2+}$	719.2245	719.2232	1.81
$[M+2H]^{2+}$	719.7262	719.7253	1.25
$[M+2H]^{2+}$	720.2279	720.2245	4.72

Di-(L-Homoalanine-L-Alanine-O-Phospho-L-Threonine-L-Proline-L-AlanineAmide)-conjugated *para*-phenolthioterephthalonitrile ($F_2$2)

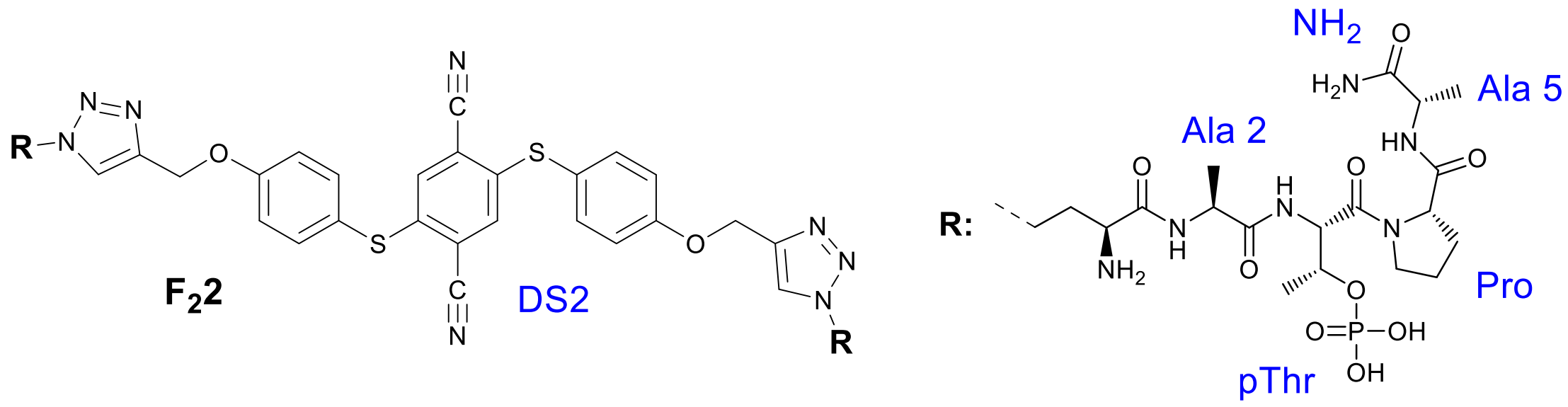

^{1}H NMR (700 MHz, 10% D_2O/ 90% H_2O): The peptidyl-prolyl and/or DS-pThr amide bonds can adopt a *trans* or *cis* conformation, separate signals of the minor conformation are marked with *. Integrals are given within the major or minor conformer, respectively. The DS2 residue comprises the fluorophore (H_{aro}), the triazole linker and the two homoalanines. Formation of soluble aggregates causes line broadening that exceeds the J coupling, thus many signals appear as broad singlets (br. s).

δ [ppm] 8.98 (br. s, 2H, NH, Ala*), 8.89 (br. s, 2H NH, Ala*), 8.51 (br. s, 2H, HN, pThr*), 8.31 (br. s, 2H, HN, Ala 5), 8.08 (s, 2H, H1, DS2), 7.96 (br. s, 2H, HN, pThr), 7.50 (br. s, 2H, HN1, NH_2), 7.44 (br. s, 2H, NH_2*), 7.29 (s, 4H, H_{aro}, DS2), 7.19 (br. s, 2H, HN2, NH_2), 7.02 (s, 2H, H_{aro}, DS2), 6.94 (s, 2H, H_{aro}, DS2*), 6.97 (s, 2H, H_{aro}, DS2), 6.90 (br. s, 2H, HN1, NH_2), 4.59 (br. s, 2H, HA, DS2) 4.44 (br. s, 2H, HA, Ala*), 4.37 (br. s, 4H, HA pThr + HB pThr), 4.29 (br. s, 2H, HA, Pro), 4.23 (t, J = 6.6 Hz, 2H, HA, Ala 5), 4.17 (br. s, 2H, QG, DS2), 3.77 (br. s, 2H, HD3, Pro), 3.54 (br. s, 2H, HD2, Pro), 2.58 (br. s, 2H, HB3, DS2), 2.50 (br. s, 2H, HB2, DS2), 2.12 (br. s, 2H, HB3, Pro), 1.82 (br. s, 2H, HG3, Pro), 1.73 (m, 4H, HG2 Pro + HB2 Pro), 1.34-1.32 (m, 12H, QB (CH_3) Ala 5 + QB (CH_3) Ala 2), 1.29 (br. s, 6H, QG (CH_3), pThr).

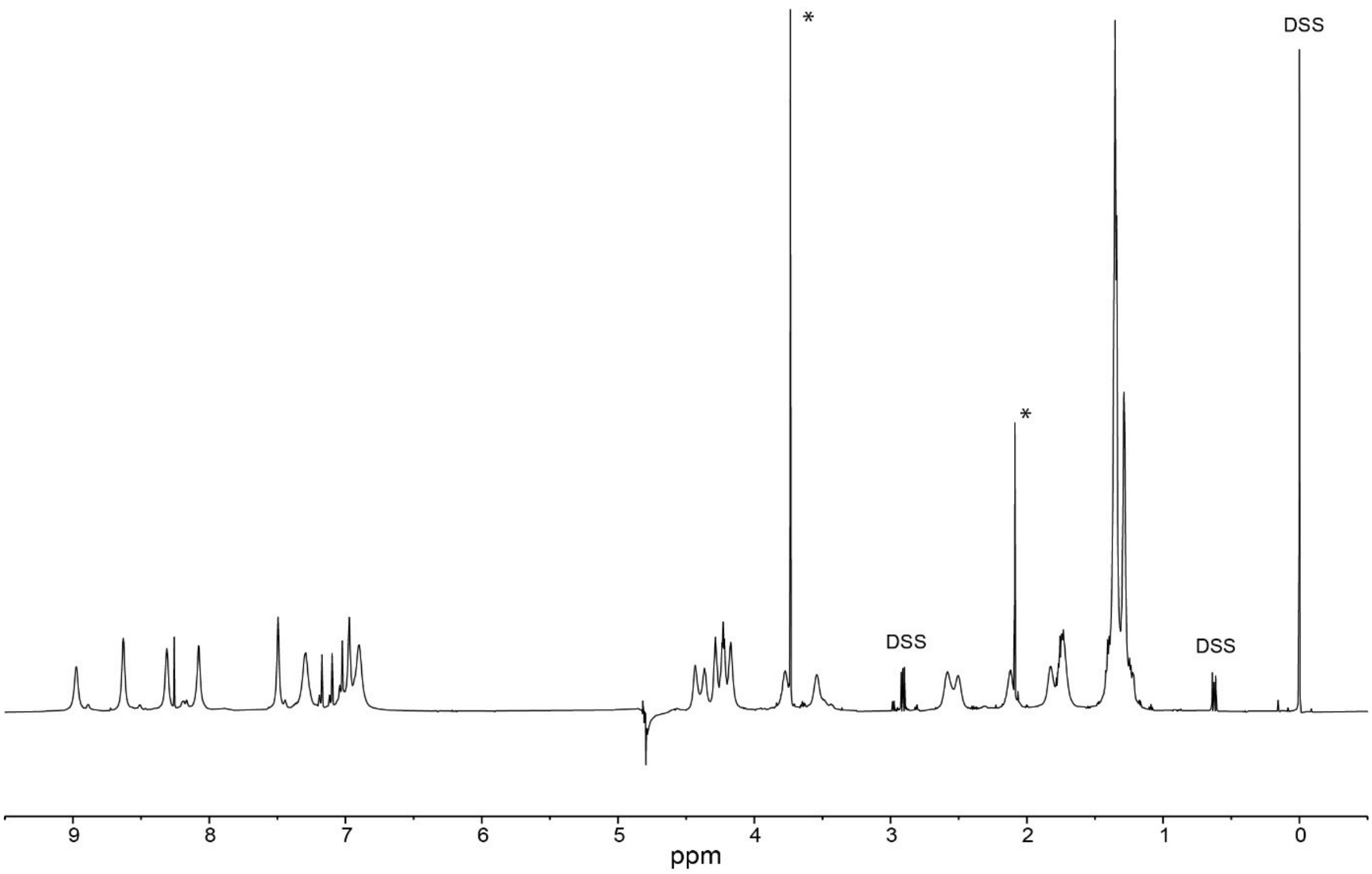

Figure S0-5. ^{1}H NMR spectrum (700 MHz, D_2O) of **$F_2$2**. * marks impurities, DSS marks the signals of the chemical shift standard.

LC-MS (ESI): calculated for $C_{64}H_{84}N_{20}O_{20}P_2S_2$, M = 1579.56. t_R= 6.04 min; *m/z*= 527.37 $[M + 3H]^{3+}$, 790.58 $[M + 2H]^{2+}$, 1579.09 $[M + H]^{+}$,

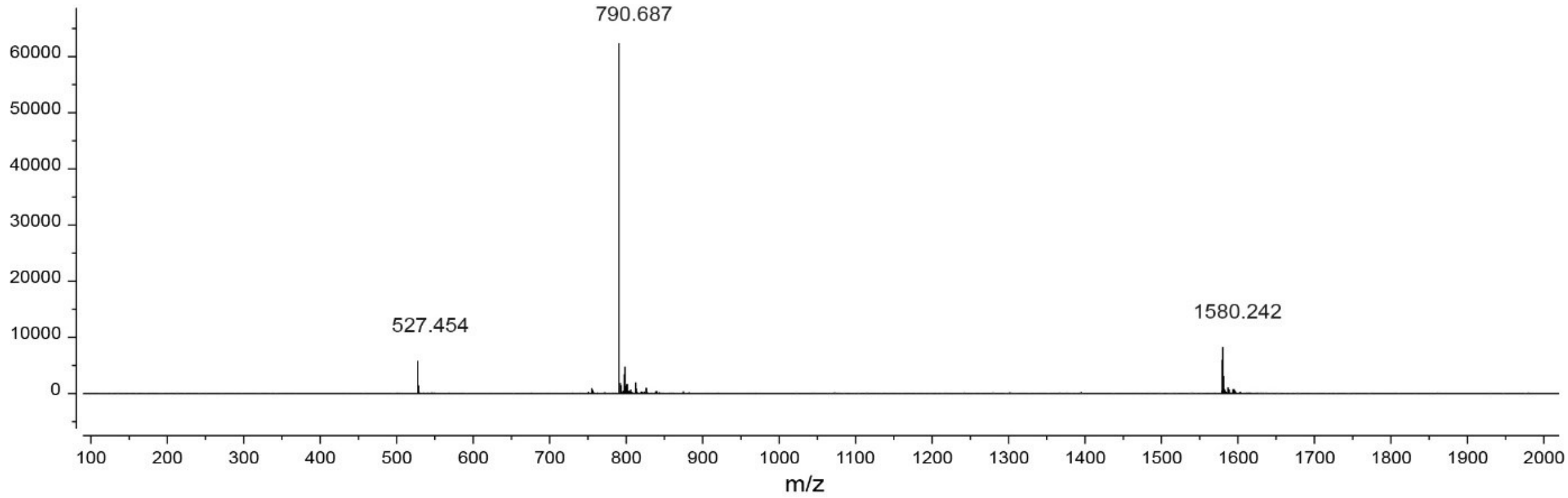

Figure S0-6. LC-MS of **F$_2$2**.

HR-MS:

Ion	*m/z* calculated	*m/z* measured	Δ [ppm]
$[M+2H]^{2+}$	790.2616	790.2594	2.78
$[M+2H]^{2+}$	790.7633	790.7621	1.52
$[M+2H]^{2+}$	791.265	791.2616	4.30

Di-(L-Homoalanine-L-Alanine-Glycin-O-Phospho-L-Threonine-L-Proline-L-Alanine-Amide)-conjugated *para*-phenolthioterephthalonitrile ($F_2$3)

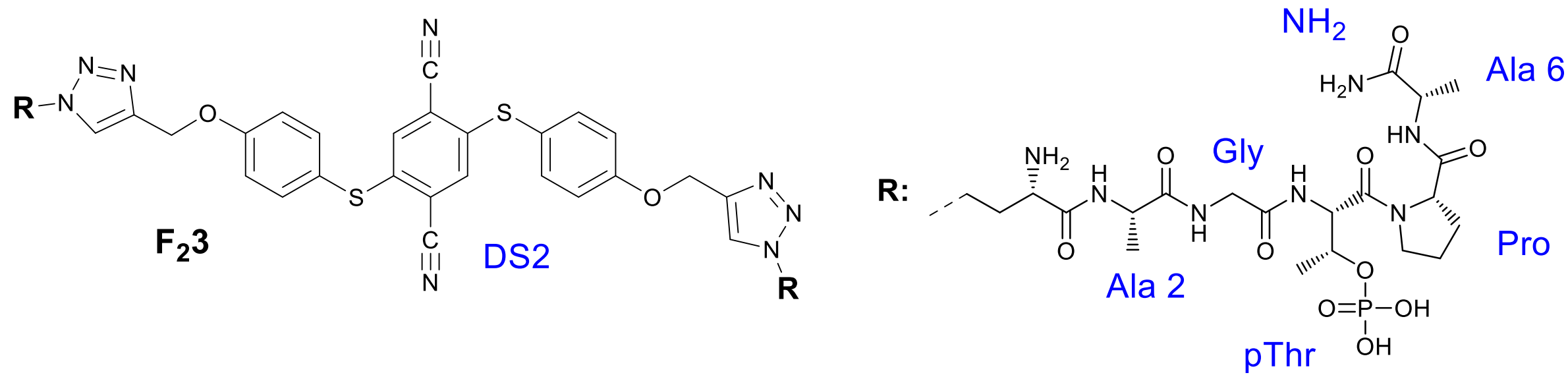

^{1}H NMR (700 MHz, 10% D_2O/ 90% H_2O): The peptidyl-prolyl and/or DS-pThr amide bonds can adopt a *trans* or *cis* conformation, separate signals of the minor conformation are marked with *. Integrals are given within the major or minor conformer, respectively. The DS2 residue comprises the fluorophore (H_{aro}), the triazole linker and the two homoalanines. Formation of soluble aggregates causes line broadening that exceeds the J coupling, thus many signals appear as broad singlets (br. s).

δ [ppm] 8.86 (br. s, 2H, HN, Ala 2), 8.54 (br. S, 2H, HN, Gly*), 8.48 (br. s, 2H, HN, Gly), 8.45 (br. s, 2H, HN, Ala 6), 8.08 (br. s, 2H, HN, pThr), 7.51 (s, 2H, HN1, NH_2), 7.28 (br. s, 4H, H_{aro}, DS2), 7.04 (br. s, 2H, H_{aro}, DS2), 7.00 (s, 2H, HN2, HN_2), 6.89 (br. s, 4H, H_{aro}, DS2), 4.68 (br. s, 2H, HA, pThr) 4.56 (br. s, 4H, QB pThr), 4.41-4.37 (m, 2H, HA, Ala 2), 4.32-4.30 (m, 2H, HA, Pro), 4.25 (m, HA, Ala 6), 4.12 (br. s, 2H, HA, DS2), 4.00 (br. s, 4H, QA, Gly*) 3.94 (br. s, 4H, QA, Gly), 3.79-3.72 (m, 2H, HD3, Pro), 3.67-3.61 (m, 2H, HD2, Pro), 2.54 (br. s, 4H, QB, DS2), 2.21 (br. s, 2H, HB3, Pro), 1.95 (br. s, 2H, HG2, Pro), 1.82 (br. s, 4H, HB2 + HG2 Pro), 1.39-.136 (m, 12H, QB (CH_3) Ala 2 + QB (CH_3) Ala 6), 1.25-1.23 (m, 6H, QG (CH_3), pThr).

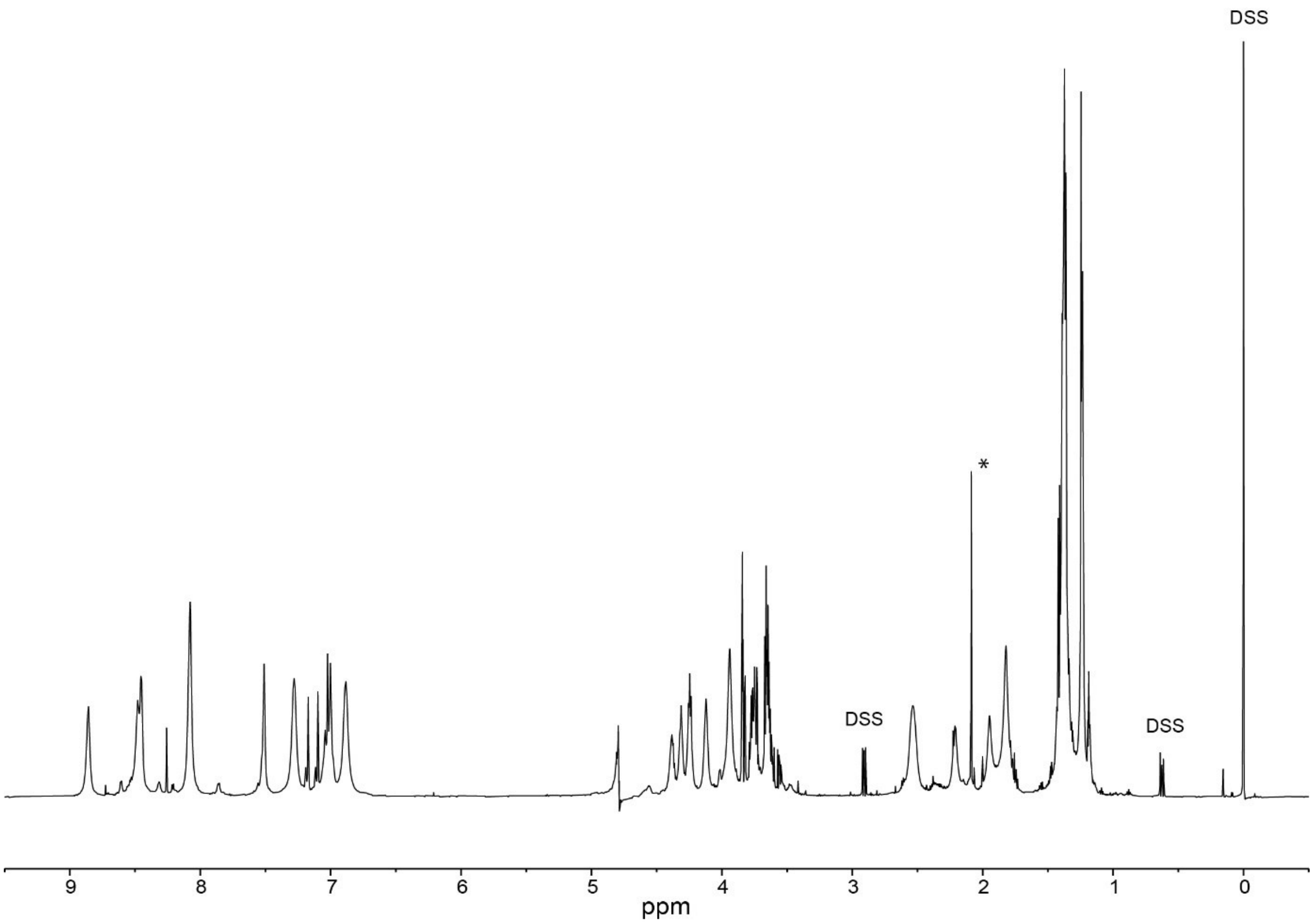

Figure S0-7. ^{1}H NMR spectrum (700 MHz, D_2O) of **$F_2$3**. * marks an impurity, DSS marks the signals of the chemical shift standard.

LC-MS (ESI): calculated for $C_{68}H_{90}N_{22}O_{22}P_2S_2$: M = 1693.67. t_R= 5.98 min; *m/z*= 565.45 $[M + 3H]^{3+}$, 848.03 $[M + 2H]^{2+}$, 1694.14 $[M + H]^{+}$.

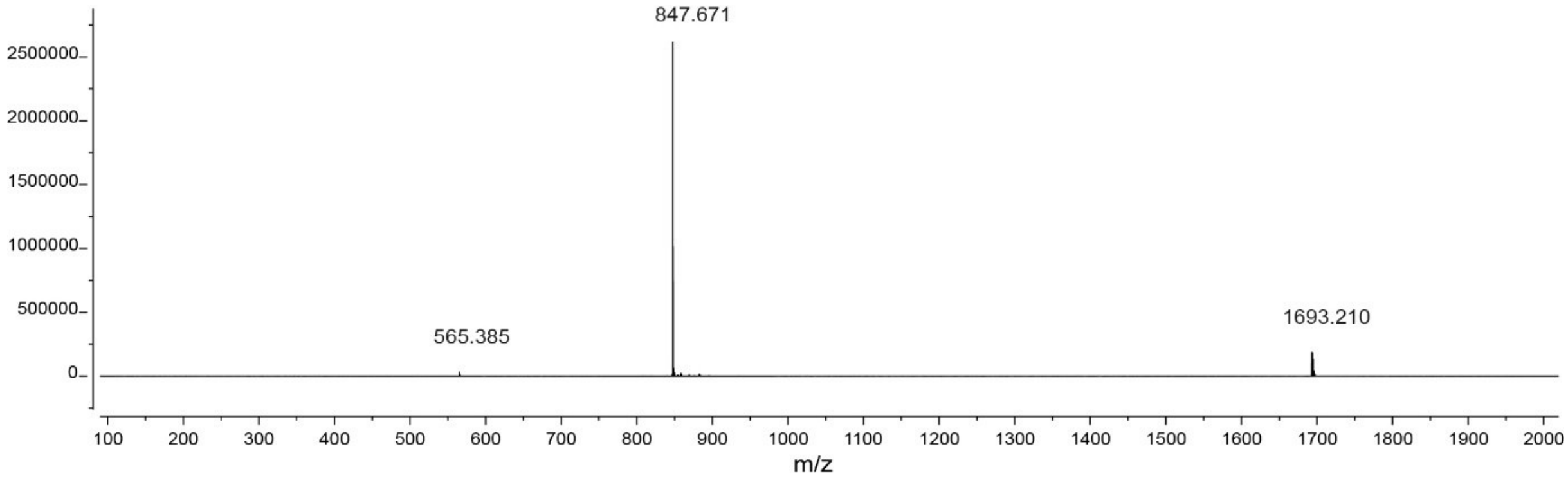

Figure S0-8. LC-MS of $\mathbf{F_2 3}$.

HR-MS:

Ion	*m/z* calculated	*m/z* measured	Δ [ppm]
$[M+2H]^{2+}$	847.2831	847.2815	1.89
$[M+2H]^{2+}$	847.7848	847.7840	0.94
$[M+2H]^{2+}$	848.2865	848.2827	4.48

Di-(L-Homoalanine-L-Alanine-Glycin-L-Alanine-Glycin-O-Phospho-L-ThreonineL-Proline-L-Alanine-Amide)-conjugated *para*-phenolthioterephthalonitrile ($F_2$4)

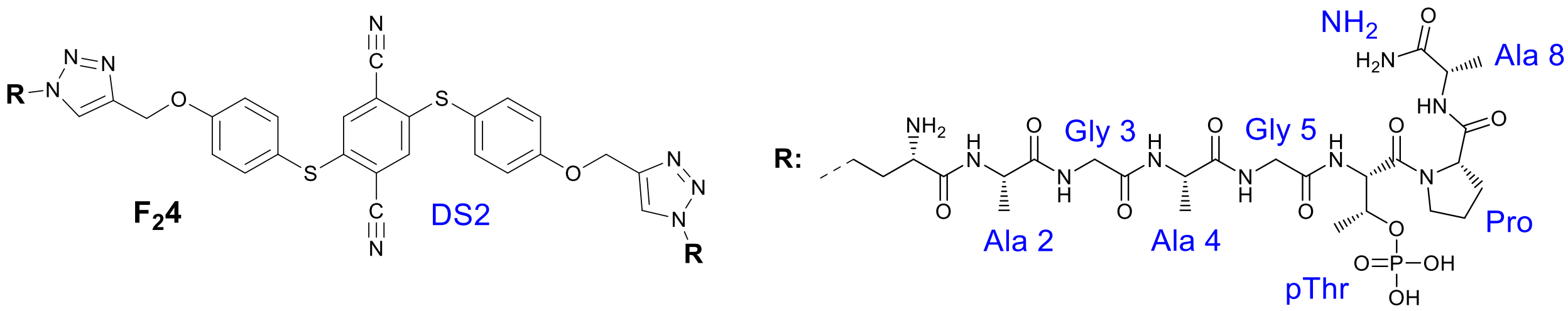

^{1}H NMR (700 MHz, 10% D_2O/ 90% H_2O): The peptidyl-prolyl and/or DS-pThr amide bonds can adopt a *trans* or *cis* conformation, separate signals of the minor conformation are marked with *. Integrals are given within the major or minor conformer, respectively. The DS2 residue comprises the fluorophore (H_{aro}), the triazole linker and the two homoalanines. Formation of soluble aggregates causes line broadening that exceeds the J coupling, thus some signals appear as broad singlets (br. s).

δ [ppm] 8.89 (s, 2H, HN, Ala 2), 8.51 (s, 2H, HN, Gly 3), 8.42 (d, J = 5.9 Hz, 2H, HN, Ala 8), 8.33 (t, J = 6.0 Hz, 2H, HN, Gly 5), 8.26 (d, J = 6.0 Hz, 2H, HN, pThr), 8.15 (d, J = 5.1 Hz, 2H, HN, Ala 4), 8.09 (s, 2H, H1, DS2), 7.56 (s, 2H, H_{aro}, DS2*), 7.52 (s, 2H, H_{aro}, DS2), 7.29 (s, 4H, HN1, NH_2), 7.02 (s, 2H, H_{aro}, DS2), 6.98 (s, 2H, H_{aro}, DS2*), 6.91 (s, 2H, HN2, NH_2), 4.52 (m, 2H, HB, pThr), 4.39-4.31 (m, 6H, HA Ala 2 +HA Pro + HA Ala 4 + HA pThr), 4.27-4.23 (m, 2H, HA, Ala 8), 4.13 (s, 2H, HA Ala*), 3.92 (s, 8H, QA Gly 3 + QA Gly 5), 3.82 (br. s, 2H, HD3, Pro), 3.70 (d, J = 8.5 Hz, 2H, HD2, Pro), 2.55 (br. s, 2H, HB3 DS2), 2.51 (br. s, 2H, HB2, DS2), 2.27-2.23 (m, 2H, HB3, Pro), 2.02-1.97 (m, 2H, HG2, Pro), 1.91-1.85 (m, 4H, HB2 Pro + HG2 Pro), 1.39-1.37 (m, 18H, QB (CH_3) Ala 2 + QB (CH_3) Ala 4 + QB (CH_3) Ala 8), 1.32-1.30 (m, 6H, QG (CH_3), pThr).

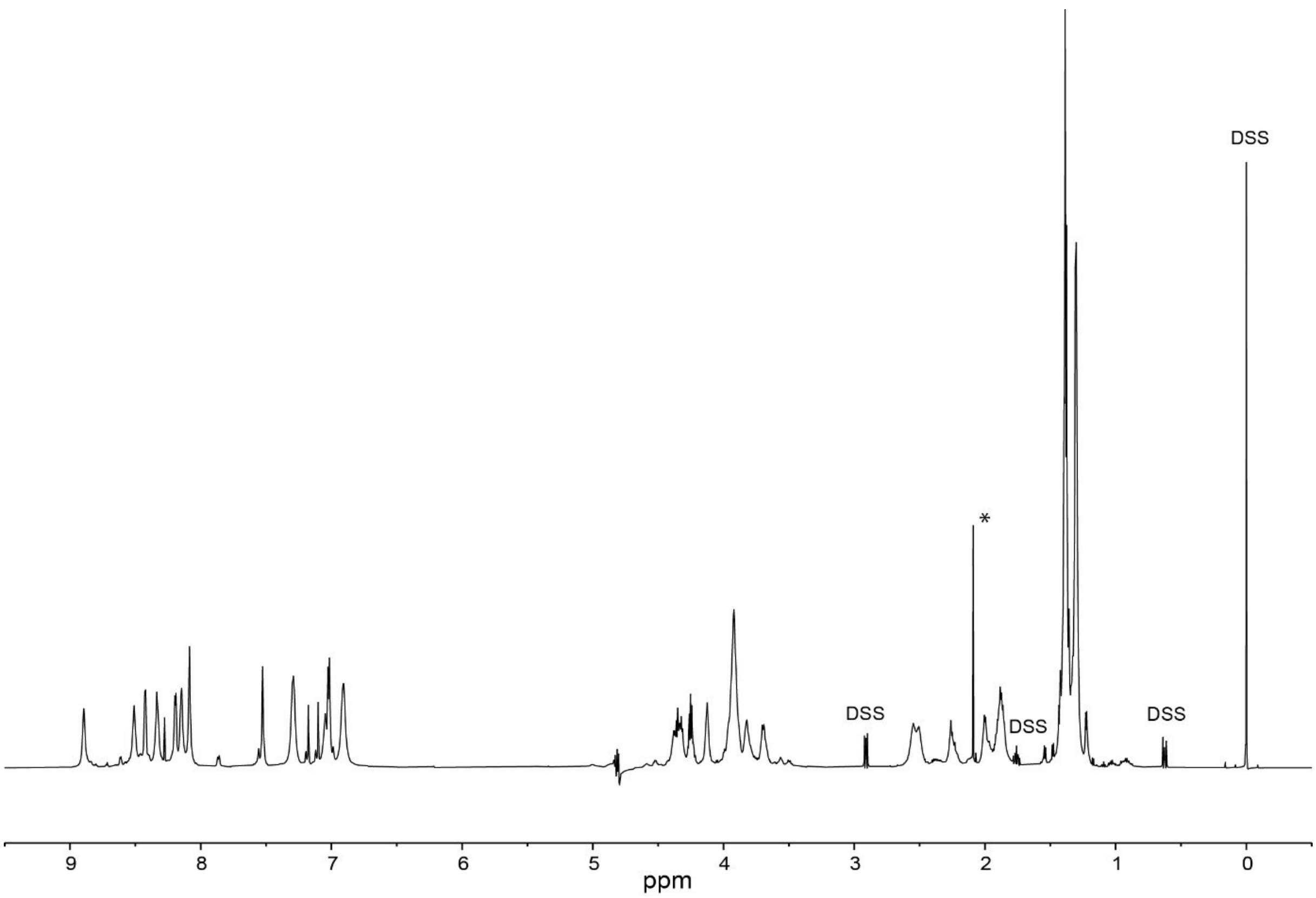

Figure S0-9. ^{1}H NMR spectrum (700 MHz, D_2O) of **$F_2$4**. * marks an impurity, DSS marks the signals of the chemical shift standard.

LC-MS (ESI): calculated for $C_{78}H_{106}N_{26}O_{26}P_2S_2$, M = 1949.93. t_R= 5.86 min; *m/z*= 975.79 [M + 2H]$^{2+}$, 1950.23 [M + H]$^{+}$.

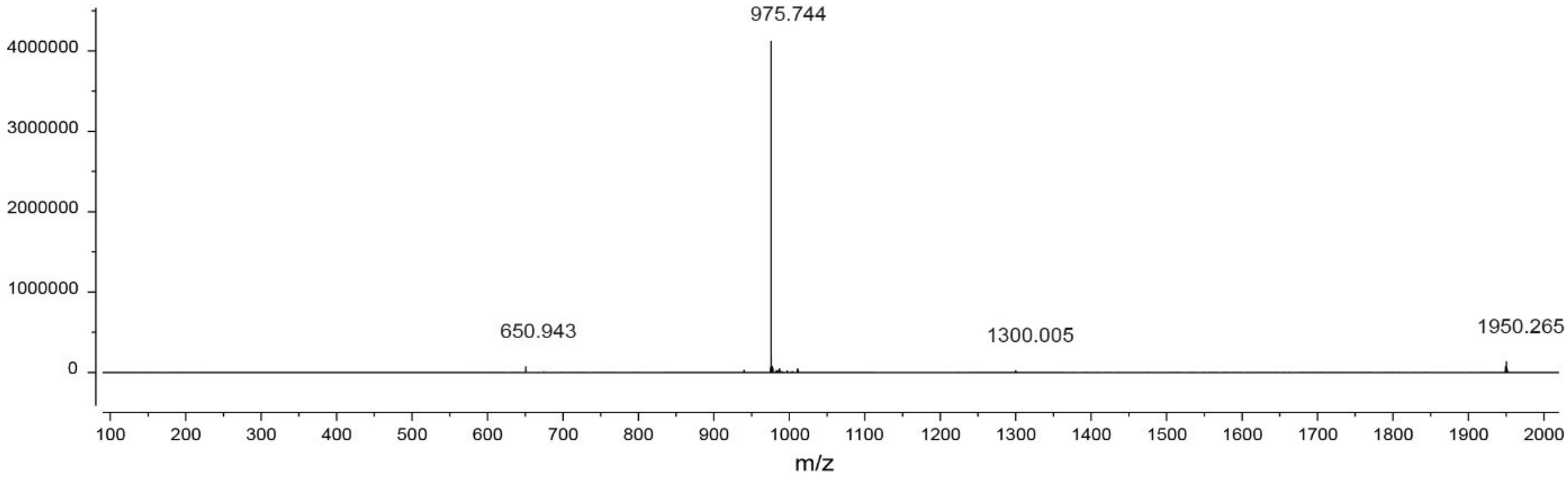

Figure S0-10. LC-MS of **$F_2$4**.

HR-MS:

Ion	*m/z* calculated	*m/z* measured	Δ [ppm]
[M+2H]$^{2+}$	975.3417	975.3380	3.79
[M+2H]$^{2+}$	975.8434	975.8378	5.74
[M+2H]$^{2+}$	976.3450	976.3360	9.22

Di-(L-Homoalanine-L-Threonine-L-Proline-L-Alanine-Amide)-conjugated paraphenolthioterephthalonitrile ($F_2$5)

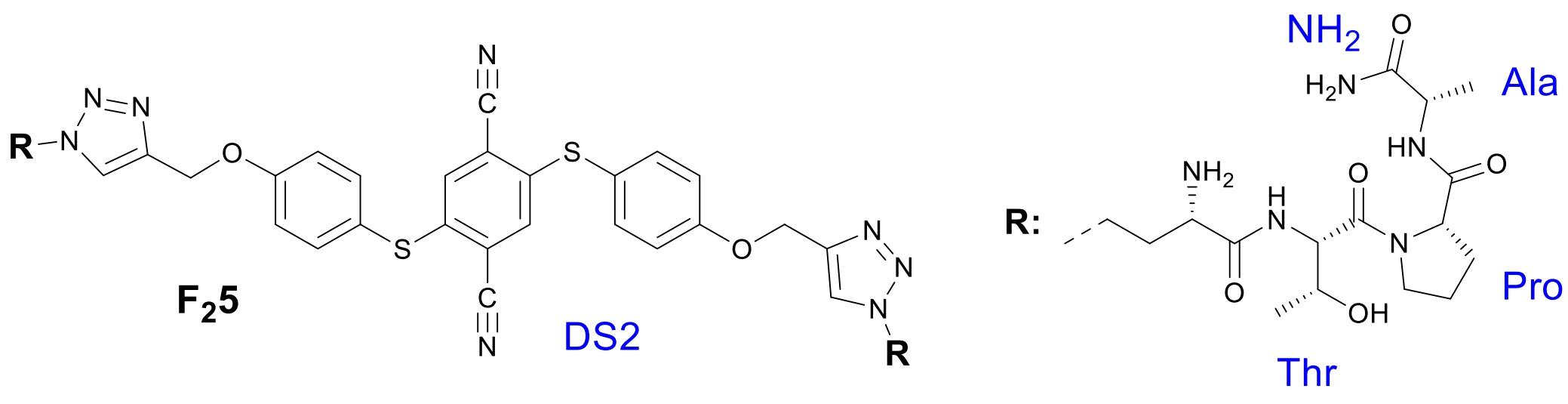

^{1}H NMR (700 MHz, 10% D_2O/ 90% H_2O): The peptidyl-prolyl and/or DS-pThr amide bonds can adopt a *trans* or *cis* conformation, separate signals of the minor conformation are marked with *. Integrals are given within the major or minor conformer, respectively. The DS2 residue comprises the fluorophore (H_{aro}), the triazole linker and the two homoalanines. A full assignment based on 2D COSY, TOCSY and NOESY spectra has not been performed.

δ [ppm] 8.75 (s, 2H), 8.29 (d, J = 6.0 Hz, 2H), 8.16 (s, 2H*), 8.14 (s, 2H), 7.51 (s, 2H), 7.46 (d, J = 8.7 Hz, 4H), 7.31 (s, 2H), 7.10 (d, J = 8.8 Hz, 4H), 7.00 (s, 4H), 5.23 (s, 2H), 4.44 (t, J = 7.7 Hz, 2H), 4.24 (qui, J = 6.9 Hz, 2H), 4.22-4.18 (m, 4H), 3.88-3.84 (m, 2H), 3.76-3.72 (m, 2H), 2.58-2.50 (m, 6H), 2.33-2.28 (m, 2H), 2.07-2.03 (m, 2H), 1.99-1.90 (m, 4H), 1.46 (d, J = 7.4 Hz, 6H*), 1.36 (d, J = 7.3 Hz, 6H), 1.30 (d, J = 6.5 Hz, 6H), 1.22 (d, J = 7.0 Hz, 6H*).

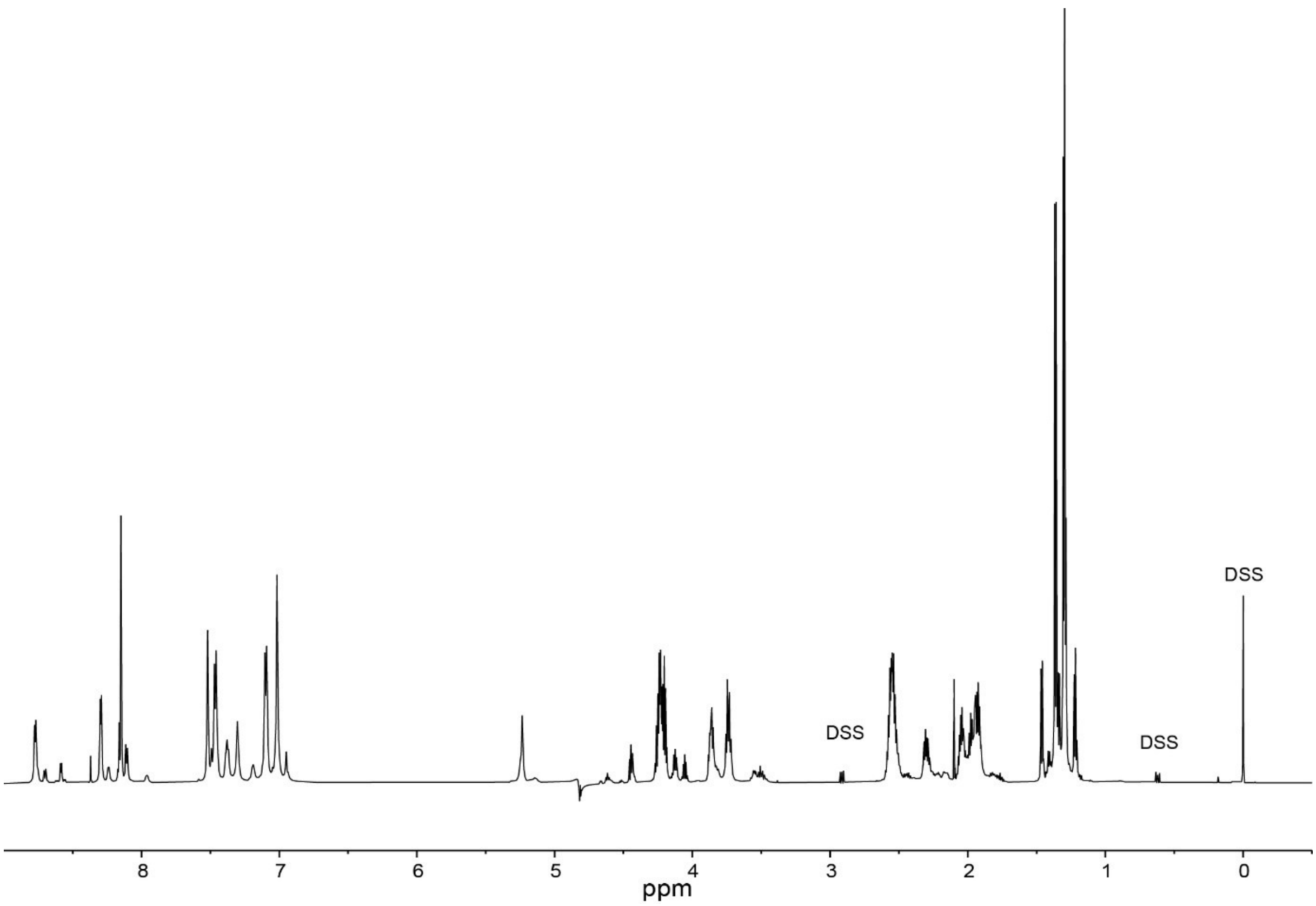

Figure S0-11. ^{1}H NMR spectrum (700 MHz, D_2O) of **$F_2$5**. DSS marks the signals of the chemical shift standard.

LC-MS (ESI): calculated for $C_{58}H_{72}N_{18}O_{12}S_2$: M = 1277.45. t_R= 5.83 min; *m/z*= 639.56 $[M + 2H]^{2+}$, 1277.24 $[M + H]^{+}$.

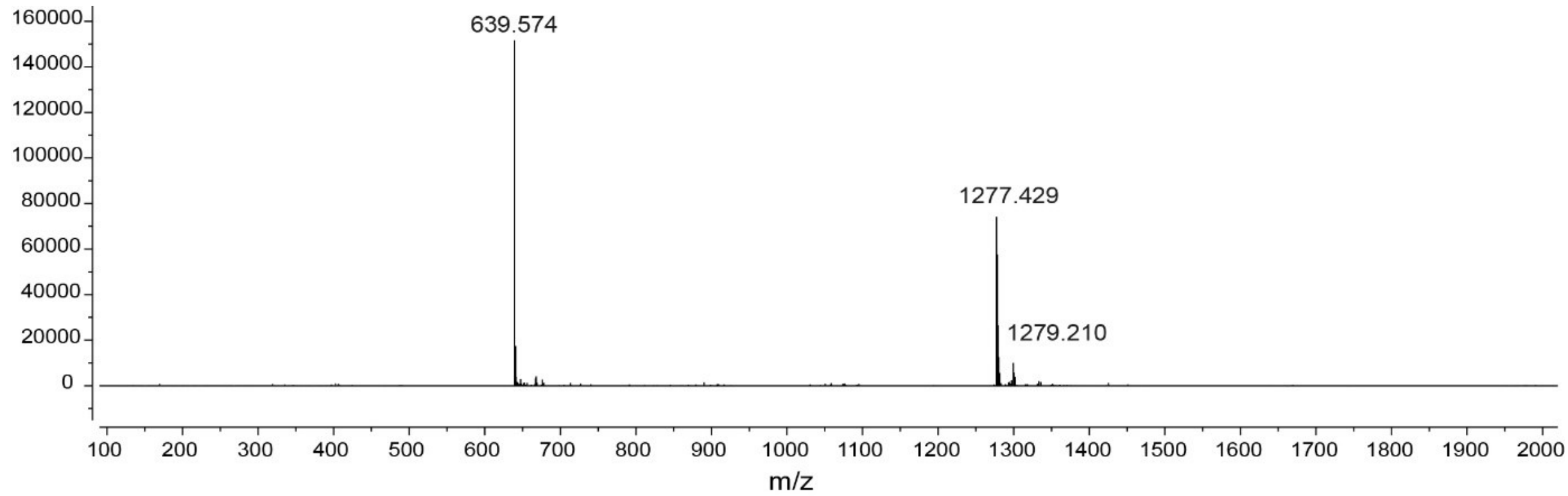

Figure S0-12. LC-MS of **F₂5**.

Di-(L-Homoalanine-L-Threonine-Glycine-L-Alanine-Amide)-conjugated paraphenolthioterephthalonitrile (F_26)

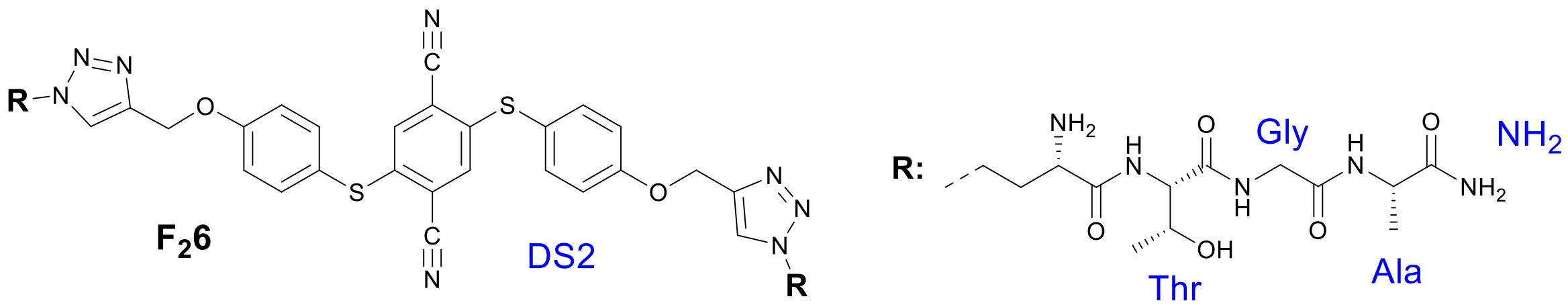

^{1}H NMR (700 MHz, 10% D_2O/ 90% H_2O): The DS2 residue comprises the fluorophore (H_{aro}), the triazole linker and the two homoalanines. A full assignment based on 2D COSY, TOCSY and NOESY spectra has not been performed.

δ [ppm] 8.74 (s, 2H), 8.41 (s, 2H), 7.89 (s, 2H), 7.75 (s, 2H), 7.14 (s, 4H), 6.89 (s, 2H), 6.73 (s, 4H), 4.30 (s, 2H), 4.15 (s, 2H), 4.09 (t, J = 5.3 Hz, 2H), 4.00 (t, J = 7.1 Hz, 4H), 3.84-3.78 (m, 6H), 2.42 (s, 4H), 1.10 (s, 6H), 1.09 (s, 6H).

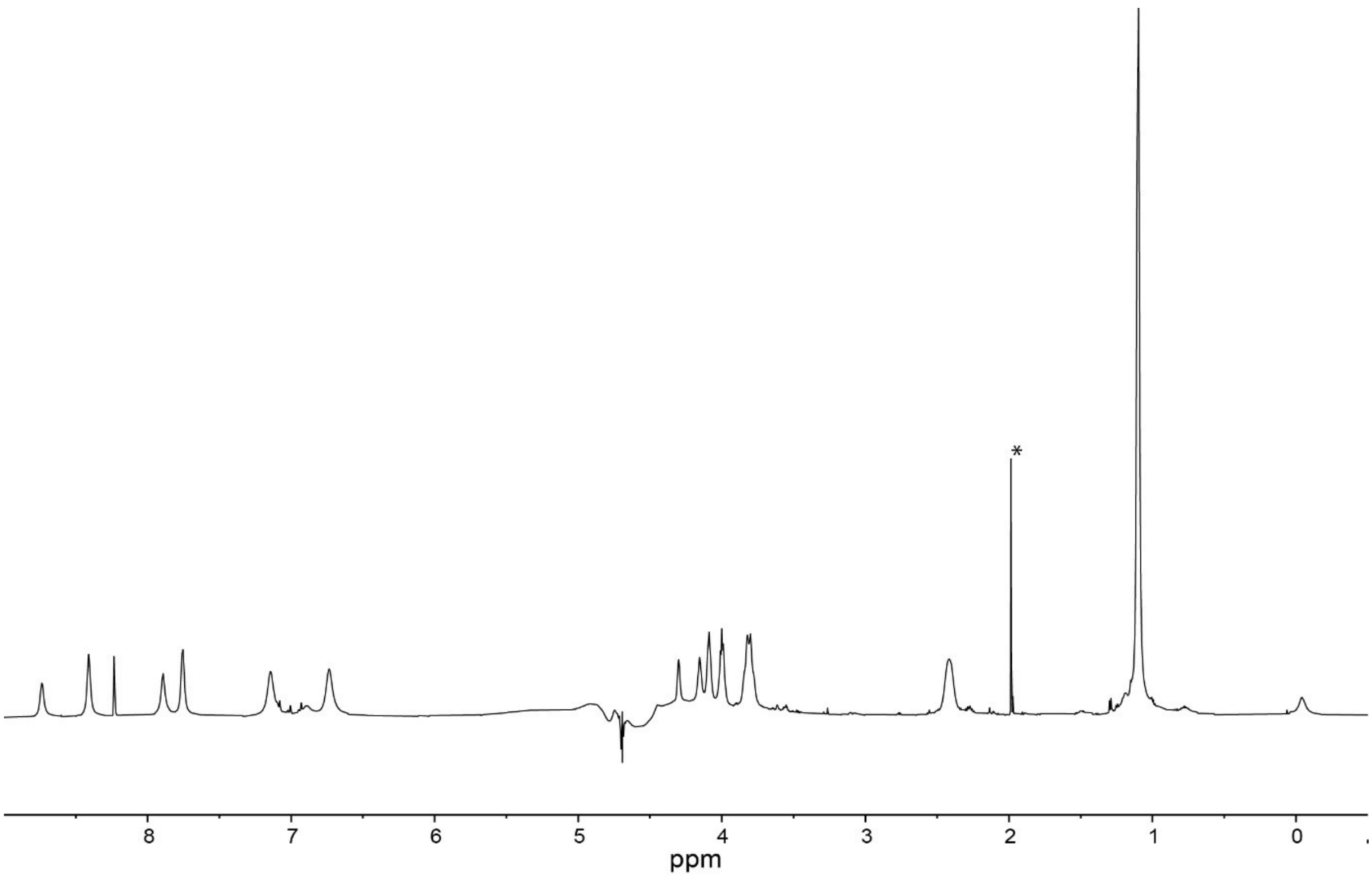

Figure S0-13. ^{1}H NMR spectrum (700 MHz, D_2O) of **F_26**. * marks an impurity.

LC-MS (ESI): calculated for $C_{52}H_{64}N_{18}O_{12}S_2$: M = 1197.32. t_R= 6.10 min; *m/z*= 599.47 $[M + 2H]^{2+}$, 1197.21 $[M + H]^{+}$,

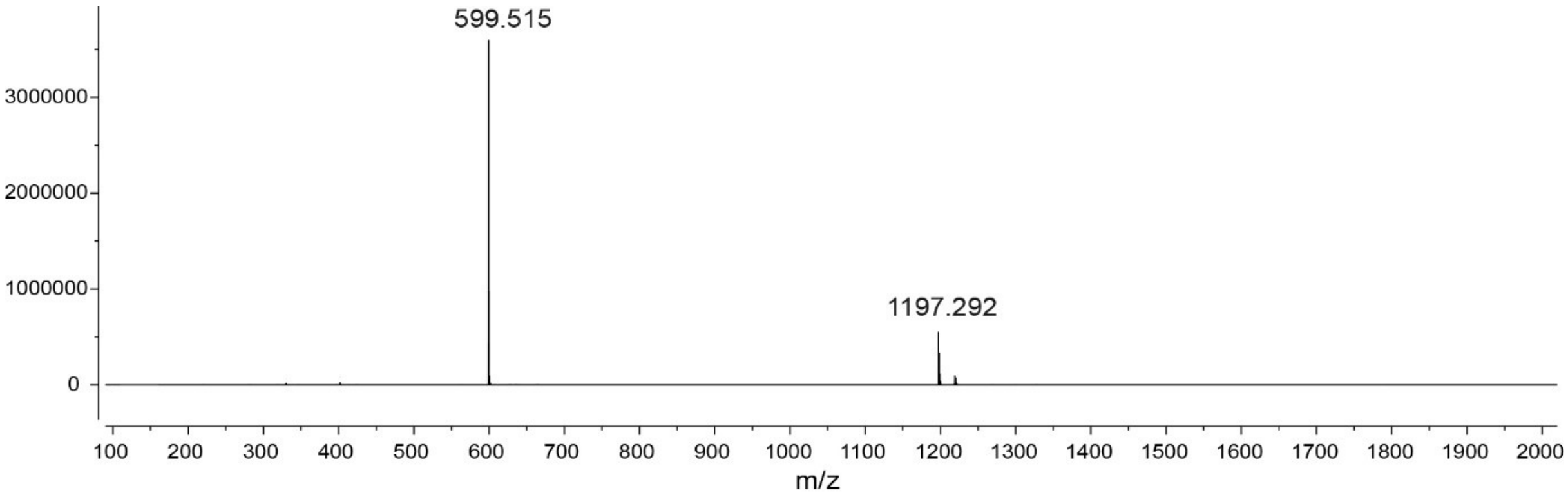

Figure S0-14. LC-MS of **$F_2$6**.

Methods

Preparative HPLC

Peptides and peptide modified fluorophores were HPLC-purified using a Prominence UFLC system (Shimadzu) with a LunaR 5 µm C18 (2) column (100 x 21.20 mm) running a linear gradient of CH_3CN *vs.* H_2O with 0.1% TFA at a flow rate of 20 mL/min. Detection was carried out at 210 nm and 254 nm. Product-containing fractions were collected manually and subsequently lyophilized.

Protein Expression and Purification.

The *h*Pin1 WW domain (residues 3-39), with an N-terminal GST tag followed by a Prescission protease site, was expressed as described[5]. Unlabelled *h*Pin1-WW for fluorescence experiments was expressed in LB media. Uniformly ^{15}N or ^{15}N and ^{13}C labelled *h*Pin1-WW for NMR experiments was expressed in M9 minimal media with $^{15}NH_4Cl$ and either ^{12}C- (for ^{15}N-only labelling) or ^{13}C-glucose (for $^{15}N,^{13}C$-labelling. The protein was purified by GSH affinity chromatography followed by cleavage of the GST tag with Prescission protease and a size exclusion chromatography. Unlabelled *h*Pin1-WW was buffer-exchanged into fluorescence buffer (20 mM HEPES, pH 7.0). ^{15}N- and $^{15}N,^{13}C$-labelled *h*Pin1-WW for NMR were buffer-exchanged into NMR buffer (50 mM KP_i pH 7.0). Protein concentrations were measured with a Nanodrop (Thermo Scientific) at 280 nm.

Mass spectrometry

LC-MS. LC-MS analysis of peptides and peptide-conjugated fluorophores was performed by a LCQ FleetTM ESI-MS with an Accela UV detector (Thermo Scientific). A reverse-phase HPLC column Eclipse XDB-C18 5 µm (Agilent) was run at a flow rate of 1 mL/min with a gradient of CH_3CN with 0.1% formic acid (FA) *vs.* H_2O with 0.1% FA.

High resolution mass spectrometry (HR-MS). For high resolution mass spectrometry an Exactive Plus Orbitrap mass spectrometer (Thermo Scientific) in combination with a TriVersa NanoMate (Advion) and a 5.5 µM ESI Chip (Fisher Scientific) was used. Measurements were performed in positive mode (resolution 140000, spray voltage 1500 V, capillary temp. 250 °C, S-lens RF level 50, AGC target 3e6). Samples were dissolved in CH_3CN/H_2O (1:1) with 0.1% FA.

UV-Vis spectroscopy

UV-Vis measurements were performed with a Varian Cary 100 Bio UV-Vis spectrometer in quartz cuvettes (Hellma) at room temperature.

Solubility test. To test the solubility of peptide-conjugated fluorophores in aqueous solution, the fluorophores (20mM) were dissolved in water and then diluted to the final concentration (20 mM down to 25 µM) in water, fluorescence buffer (20 mM HEPES pH 7.0) or PBS buffer (pH 7.4). The turbidity at 600 nm was measured at different fluorophore concentrations. A turbidity > 0 indicates solid aggregates.

Determination of extinction coefficients. To determine the extinction coefficient of soluble fluorophores, a dilution series (1 mM down to 10 µM) of fluorophore in water was prepared. The Extinction at 400 nm (E_{400}) was measured in triplicate and the extinction coefficient ε_{400} was calculated according to Lambert-Beer's law: $E_{400} = \varepsilon_{400} \cdot c \cdot d$ (with c = concentration; d = pathlength).

Table S0-3: Extinction coefficients of AIE-peptides.

AIE-peptide	$F_1$1	$F_2$1	$F_2$2	$F_2$3	$F_2$4	$F_2$5	$F_2$6
$\varepsilon_{400\,nm}$ [$M^{-1}cm^{-1}$]	3469 ± 27	3411 ± 21	3167 ± 26	3779 ± 28	3472 ± 21	3384 ± 12	3248 ± 36

Fluorescence spectroscopy

Fluorescence measurements were carried out on a JASCO FP-8300 fluorescence spectrometer at 25°C in micro quartz cuvettes (Hellma). The detector voltage was adjusted depending on the fluorophore concentration. 10 measurements were averaged for each data point.

AIE sample preparation. AIE Fluorophore stock solutions were prepared at a concentration of 1 mM in H_2O and then diluted to the final concentration in the respective buffer (see below) 24 h before each measurement.

AIE Autofluorescence. To determine the working concentration for titration experiments, the fluorophores were tested for autofluorescence in H_2O, fluorescence buffer (20 mM HEPES pH 7.0) or PBS buffer at various concentrations (10 µM to 1 mM). Ideal concentrations for fluorescence binding experiments show low autofluorescence in the absence of protein and lie in the range where autofluorescence does not increase with concentration (which is a sign for aggregation).

AIE Fluorescence Titrations. To characterize the binding of *h*Pin1-WW to the peptide-conjugated AIE fluorophores, the protein was titrated to the fluorophores and the change in fluorescence intensity was detected at λ_{ex}= 399nm, λ_{em}= 489 nm. Titrations were performed at various fluorophore concentrations in fluorescence buffer (20 mM HEPES pH 7.0).

To avoid dilution effects, the fluorophore was added to the titrant stock in the same concentration as in the cuvette. Fluorescence titrations were performed in triplicate and data points were averaged. Error bars of each data point represent the standard deviation of the mean. The change in fluorescence intensity was plotted against the protein concentration and the binding curve was fitted with equation 1, yielding the apparent dissociation constant $K_{D,app}$. The stoichiometric factor n was kept constant at the theoretical value (n = 1 for F11 and n = 2 for **F₂1** – **F₂4**). If the fit did not match the data points, the fitting was repeated with variable n, yielding n values much smaller (> ± 0.2) than the theoretical value, which indicates aggregation of the AIE-peptide. Errors for $K_{D,app}$ and n represent the standard error of the fit.

$$F=F_{max}\cdot\frac{(c_{AIE}+x/n+K_D)-\sqrt[2]{(c_{AIE}+x/n+K_D)^2-(4\cdot x/n\cdot c_{AIE})}}{2\cdot c_{AIE}} \qquad (1)$$

With x = protein concentration, F= fluorescence intensity change, F_{max} = fluorescence amplitude, K_D = dissociation constant, n = stoichiometry factor (proteins per AIE-peptide), c_{AIE} = AIE-peptide concentration (constant).

Fluorescence Anisotropy Titrations. The control titration of the **P0** peptide without AIE was performed by fluorescence anisotropy with 100 nM N-terminally 5,6-FAM labelled peptide (**FAM-P0**) in fluorescence buffer (20 mM HEPES pH 7.0). The protein (with 100 nM **FAM-P0** to avoid fluorophore dilution) was titrated to **FAM-P0** and the fluorescence anisotropy was detected at λ_{ex}= 495 nm and λ_{em}= 518 nm. Fluorescence anisotropy titrations were performed in triplicate and data points were averaged. Error bars of each data point represent the standard deviation of the mean. The binding data was fitted with equation 2 and the stoichiometric factor n was kept constant at n = 1. Errors for $K_{D,app}$ represent the standard error of the fit. Errors for $K_{D,app}$ represent the standard error of the fit.

$$r = r_0 + r_{max}\cdot\frac{(c+x/n+K_D)-\sqrt[2]{(c+x/n+K_D)^2-(4\cdot x/n\cdot c)}}{2\cdot c} \qquad (2)$$

With x = protein concentration, r= fluorescence anisotropy, r_0 = offset, r_{max} = Amplitude, K_D = dissociation constant, n = stoichiometry factor (proteins per peptide), c = **FAM-P0** concentration (constant).

Tryptophan Fluorescence Titrations. *h*Pin1-WW (1 µM) in fluorescence buffer (20 mM HEPES pH 7.0) was titrated with the control peptide **P0** (with 1 µM *h*Pin1-WW to avoid fluorophore dilution). Trp emission spectra (λ_{ex} = 295 nm, λ_{em} = 300-400 nm) were measured for each peptide concentration. The fluorescence intensity at λ_{em} = 335 nm was then plotted against the peptide concentration. Trp fluorescence titrations were performed in triplicate and data points were averaged. Error bars of each data point represent the standard deviation of the mean. The binding data was fitted with equation 3 and the stoichiometric factor n was kept constant at n = 1. Errors for $K_{D,app}$ represent the standard error of the fit. Errors for $K_{D,app}$ represent the standard error of the fit.

$$F = F_0 - F_{max} \cdot \frac{(c + x \cdot n + K_D) - \sqrt[2]{(c + x \cdot n + K_D)^2 - (4 \cdot x \cdot n \cdot c)}}{2 \cdot c} \quad (3)$$

With x = peptide concentration, F= fluorescence intensity, F_0 = offset, F_{max} = Amplitude, K_D = dissociation constant, n = stoichiometry factor (proteins per peptide), c = *h*Pin1-WW concentration (constant).

NMR spectroscopy

^{1}H, 2D homonuclear, 2D ^{1}H,^{15}N-HSQC and 3D ^{13}C-NOESY NMR experiments were recorded on a Bruker Avance NEO 700 MHz NMR spectrometer equipped with a 5 mm TCI cryoprobe with z-gradient at 298 K. ^{19}F-NMR experiments were recorded on a Bruker Avance Neo II 500MHz NMR spectrometer with a DiffBBI probe. All pulse programs were used from the Bruker pulse program library.

Spectra were processed with Topspin 3.5 and analysed in SPARKY[6] or CARA[7].Proton chemical shifts were referenced to internal 2,2-dimethyl-2-silapentane-5-sulfonic acid sodium salt (DSS). ^{13}C and ^{15}N chemical shifts were referenced indirectly to DSS using the absolute frequency ratios.[8]

Compound characterization. AIE-conjugated peptides **$F_1$1** and **$F_2$1-$F_2$6** were characterized in H_2O with 10% D_2O at a concentration of 1 mM on the 700 MHz NMR spectrometer. Full assignments of **$F_1$1** and **$F_2$1-$F_2$4** were performed using 2D-COSY, TOCSY and NOESY spectra. Complete exchange of the TFA counter ion against chloride (and thus absence of ^{19}F) was confirmed on the 500 MHz NMR spectrometer.

^{1}H,^{15}N-HSQC NMR titrations. Protein samples for NMR titrations contained ^{15}N-labelled *h*Pin1-WW (400 µM) in NMR buffer (50 mM KP_i pH 7.0) with 10% D_2O. A 2 mM stock solution of AIE-conjugated peptide or peptide without AIE fluorophore was added stepwise to the protein and a ^{1}H,^{15}N-HSQC spectrum was recorded for each titration step. The total chemical shift perturbation $\Delta\delta_{total}$ was calculated according to equation 4.[9]

$$\Delta\delta_{total} = \sqrt{(\Delta\delta_H)^2 + (0.154 \cdot \Delta\delta_N)^2} \quad (4)$$

With $\Delta\delta_{total}$ = total chemical shift perturbation (signal shift), $\Delta\delta_H$ = signal shift in ^{1}H direction, $\Delta\delta_N$ = signal shift in ^{15}N direction.

The total chemical shift perturbation $\Delta\delta_{total}$ was then plotted against the AIE-peptide or peptide concentration and the K_D values and stoichiometric factors n were fitted for residues Q33, W34 and E35 using equation 5. In contrast to equation 1, x describes the titrated AIE-peptide concentration, therefore, x needs to be multiplied with stoichiometry factor n (as opposed to divided by, which is the case for the reverse experiment when protein is titrated to the AIE-peptide)

$$\Delta\delta = \Delta\delta_{max} \cdot \frac{(c_{protein} + x \cdot n + K_D) - \sqrt[2]{(c_{protein} + x \cdot n + K_D)^2 - (4 \cdot x \cdot n \cdot c_{protein})}}{2 \cdot c_{protein}} \quad (5)$$

With x = AIE-peptide concentration, $\Delta\delta$ = chemical shift perturbation, $\Delta\delta_{max}$ = chemical shift perturbation amplitude, K_D = dissociation constant, n = stoichiometry factor (proteins per AIE-peptide), $c_{protein}$ = protein concentration (constant).

Structure Determination of AIE-Peptide $F_1$1. For the assignment and structure determination of **$F_1$1** (1 mM in H_2O/10% D_2O) 2D-homonuclear COSY, TOCSY and NOESY (mixing time 200 ms) spectra were recorded.

Structure Determination of the *h*Pin1-WW:$F_1$1 Complex. Complex samples contained a 1:1 mixture of *h*Pin1-WW and **$F_1$1** in NMR buffer (50 mM KP_i pH 7.0) in 100% D_2O. Aliphatic (C_{ali}) and aromatic (C_{aro}) ^{13}C-NOESY spectra (mixing time 100 ms) were recorded, yielding intra- and intermolecular protein NOEs. Aliphatic and aromatic filter-edited ^{13}C-NOESY spectra[10–13] (mixing time 100 ms) yielded intermolecular NOEs. For the assignment and intramolecular NOEs of the bound **$F_1$1**, double-filtered 2D-TOCSY and NOESY (mixing time 100 ms) experiments[14,15] were used.

NMR structure calculations

NMR Solution Structure Calculation of AIE-Peptide $F_1$1. Sequential backbone and side chain ^{1}H chemical shift assignments of fluorophore **$F_1$1** were obtained from 2D-COSY, TOCSY and NOESY spectra. NOESY cross-peaks for the structure calculation were picked and assigned manually and integrated in SPARKY[6]. CYANA parameter files for the non-standard residues phosphothreonine (TPOH), the amidated C-terminus (-NH_2, AMI) and the fluorophore-conjugated homoalanine (DS) were created manually (for full parameter files see section "Non-standard Residue Parameter Files (CYANA Library Files)" below). The structure of fluorophore **$F_1$1** was calculated in CYANA[16] using the NOE peak list as input restraints. For the input and structure calculation parameters see Table S1. The structure of unbound **$F_1$1** has been deposited in the Protein Data Base under the pdb ID 29KP (extended ID: pdb_000029KP), and the NMR data are available in the BMRB database under the accession number 35045.

NMR Solution Structure Calculation of *h*Pin1-WW in complex with $F_1$1. The chemical shift assignments of the *h*Pin1 WW domain (residues 3-39) were obtained from BMRB entry #1925 and were checked and adjusted using the 3D ^{13}C-NOESY spectra ($^{13}C_{ali}$-NOESY, $^{13}C_{aro}$-NOESY). Assignments for the bound AIE-peptide **$F_1$1** were performed using double-filtered 2D-COSY and NOESY spectra. Backbone dihedral angles for *h*Pin1-WW were calculated from the chemical shifts using TALOS+[17,18]. For the complex structure calculation, distance restraints were obtained from unfiltered 3D ^{13}C-NOESY spectra ($^{13}C_{ali}$-NOESY, $^{13}C_{aro}$-NOESY) yielding protein-protein and protein-ligand NOEs, filter-edited 3D $^{13}C_{ali}$- and $^{13}C_{aro}$-NOESYs yielding protein-ligand NOEs only, and a double-filtered 2D-NOESY yielding intra-ligand NOEs only. NOESY peak picking and assignment for the double-filtered 2D-NOESY was performed manually. NOESY peak picking and initial assignments for the filter-edited NOESY spectra was performed with the ATNOS/CANDID module of the UNIO 2.9.1 software[19–21] and initial NOE assignments were then checked and adjusted manually. H-bond restraints were set manually for the β-sheet region of *h*Pin1-WW. The structure calculation was performed in UNIO 2.9.1[19–21] in conjunction with CYANA 3.98[16]. Since CYANA only handles a single peptide chain, the N-terminus of the **$F_1$1** peptide arm was connected to the *h*Pin1-WW C-terminus via 10 linker units in the sequence file. For the input and complex structure calculation parameters see Table S4. The complex interface was analysed with the 'Protein interfaces, surfaces and assemblies' service PISA[22] at the European Bioinformatics Institute (https://wwwdev.ebi.ac.uk/pdbe/pisa/). The structure of *h*Pin1-WW bound to **$F_1$1** has been deposited in the Protein Data Base under the pdb ID 29KQ (extended ID: pdb_000029KQ), and the NMR data are available in the BMRB database under the accession number 35046.

Supplemental Tables

Table S1: Input for the structure calculation (constraints) and characterization of the ensembles of 30 CYANA conformers used to represent the NMR structure of the AIE-peptide fluorophore $\mathbf{F_1 1}$.

**Parameter**	**Value**
Total number of structures computed	200
Number of structures analysed	30
Number of chemical shifts	31
Constraints	
NOE upper distance limits	
Total	76
intra-residue [i = j]	30
sequential [|i - j| = 1]	23
medium range [1 ≤ |i - j| ≤ 4]	23
Residual Target function Value [Å^2]	0.81 ± 0.02
Residual constraint violations	
Distance violations	
0.1 – 0.2 Å	1
> 0.2 Å	0
Mean [Å]	0.18
Maximum [Å]	0.23
Van der Waals violations (≥ 0.2 Å)	5
Mean [Å]	0.14
Maximum [Å]	0.30
Dihedral angle violations (≥ 5 deg)	0
RMSD to mean structure [Å]	
All backbone atoms (1-5)	0.25 ± 0.09
All heavy atoms (1-5)	0.80 ± 0.12
Ramachandran plot statistics [%]	
Most favoured regions	100
Additionally allowed regions	0
Generously allowed regions	0
Disallowed regions	0

Table S2: Dissociation constants (K_D) of selected amino acids from the *h*Pin1-WW peptide binding site upon binding of AIE-peptides **$F_1$1** and **$F_2$1-$F_2$4** and control peptide **P0** determined by NMR titrations. The stoichiometry factor n (proteins per AIE-peptide; n = 1 for **$F_1$1** and **P0**; n = 2 for **$F_2$1-$F_2$4**,) was kept constant. Errors for the single residue K_Ds represent the standard error of the fit, while the error of the averaged K_D represents the standard error of the average.

AIE-peptide	Q33 K_D [µM]	W34 K_D [µM]	E35 K_D [µM]	Average K_D [µM]
**$F_1$1**	38 ± 7	23 ± 6	19 ± 5	27 ± 8
**$F_2$1**	39 ± 8	19 ± 7	24 ± 9	27 ± 8
**$F_2$2**	56 ± 3	37 ± 2	32 ± 3	42 ± 10
**$F_2$3**	32 ± 4	31 ± 6	26 ± 7	30 ± 3
**$F_2$4**	18 ± 2	22 ± 3	20 ± 3	20 ± 2
**P0**	19 ± 6	18 ± 7	18 ± 7	18.2 ± 0.2

Table S3: Apparent dissociation constants ($K_{D,app}$) and stoichiometry factors n (proteins per AIE-peptide) from fluorescence titrations of AIE-peptides **$F_1$1** and **$F_2$1-$F_2$4** with *h*Pin1-WW at varying AIE-peptide concentrations. Errors represent the standard error of the fit.

c [µM]	**$F_1$1**		**$F_2$1**		**$F_2$2**		**$F_2$3**		**$F_2$4**	
	$K_{D,app}$ [µM]	n ($n_{theor.}$ = 1)	$K_{D,app}$ [µM]	n ($n_{theor.}$ = 2)	$K_{D,app}$ [µM]	n ($n_{theor.}$ = 2)	$K_{D,app}$ [µM]	n ($n_{theor.}$ = 2)	$K_{D,app}$ [µM]	n ($n_{theor.}$ = 2)
10	n.d. [a]	n.d. [a]	4.0 ± 0.8	2 [b]	6.9 ± 0.7	2 [b]	5.8 ± 0.7	2 [b]	4.4 ± 0.5	2 [b]
25	12 ± 4	1 [b]	4.8 ± 0.9	2 [b]	8.0 ± 0.8	2 [b]	1.6 ± 0.4	2 [b]	2.7 ± 0.7	2 [b]
50	7 ± 4	1 [b]	2.7 ± 0.7	2 [b]	4.5 ± 0.4	2 [b]	5 ± 2	1.37 ± 0.09 [c]	23 ± 5	0.92 ± 0.08 [c]
100	2.3 ± 1.5	1 [b]	2.0 ± 0.6	1.37 ± 0.02 [c]	9 ± 2	1.39 ± 0.06 [c]	6 ± 4	0.79 ± 0.06 [c]	19 ± 8	1.08 ± 0.09 [c]
150	n.d. [a]	n.d. [a]	4 ± 1	1.35 ± 0.02 [c]	12. ± 3	1.22 ± 0.03 [c]	36 ± 5	0.58 ± 0.02 [c]	16 ± 4	0.75 ± 0.03 [c]
200	n.d. [a]	n.d. [a]	31 ± 8	0.89 ± 0.05 [c]	17 ± 5	1.09 ± 0.05 [c]	50 ± 20	0.37 ± 0.04 [c]	60 ± 20	0.56 ± 0.04 [c]

[a] n.d. = not determined

[b] n kept constant at the theoretical value (n=1 for **$F_1$1**, n = 2 for **$F_2$1**-**$F_2$4**) during fit

[c] n deviating more than ± 0.2 from the theoretical value indicates aggregation

Table S4: Input for the structure calculation (constraints) and characterization of the ensembles of 20 UNIO conformers used to represent the NMR structure of the *h*Pin1-WW domain bound to the AIE-peptide fluorophore $\mathbf{F_1 1}$.

Parameter	Value
Total number of structures computed	80
Number of structures analysed	20
Number of chemical shifts	407
Constraints	
NOE upper distance limits	
Total	377
intra-residue [i = j]	86
sequential [|i - j| = 1]	57
medium range [1 ≤ |i - j| ≤ 4]	33
long range (|i-j|≥5)	201
intermolecular	35
Dihedral angle restraints [a]	80
Residual Target function Value [$Å^2$]	2.70 ± 0.29
Residual constraint violations [b]	
Distance violations (≥ 0.2 Å)	5 ± 1
Maximum [Å]	0.33 ±0.06
Van der Waals violations (≥ 0.2 Å)	6 ± 1
Maximum [Å]	0.3 ± 0.1
Dihedral angle violations (≥ 5 deg)	0.5 ± 0.5
Maximum [deg]	5.0 ± 0.8
RMSD to mean structure [Å] [b]	
All backbone atoms (*h*Pin1-WW 7-36, $\mathbf{F_1 1}$ 2-4)	0.52 ± 0.10
All heavy atoms (*h*Pin1-WW 7-36, $\mathbf{F_1 1}$ 2-4)	1.01 ± 0.10
Ramachandran plot statistics [%] [b]	
Most favoured regions	78.6
Additionally allowed regions	19.2
Generously allowed regions	1.8
Disallowed regions	0.3

[a] from TALOS; [b] from UNIO

Table S5: PISA interface analysis. Accessible (ASA) and Buried (BSA) surface area of the *h*Pin1-WW:**$F_1$1** complex by residue.

**Molecule (Chain ID)**	**Residue**	**ASA [Å^2]**	**BSA [Å^2]**	**Relative BSA [%]**
*h*Pin1-WW (A)	E 12	50.4	9.5	19
	R 14	115.1	49.6	43
	M 15	124.8	42.7	34
	S 16	10.7	9.3	87
	R 17	230.7	58.6	25
	S 18	103.3	44.0	43
	Y 23	49.1	43.5	89
	F 25	50.8	49.3	97
	H 27	108.9	25.5	23
	N 30	133.5	29.8	22
	A 31	43.7	6.4	15
	S 32	75.0	34.1	45
	W 34	127.9	68.8	54
**$F_1$1** (B)	DS* 1	780.2	314.9	40
	pT 2	154.8	75.7	49
	P 3	108.3	64.0	59
	A 4	126.2	56.8	45
	NH2 5	116.9	40.7	35

* DS = AIE-conjugated homoalanine

Supplemental Figures

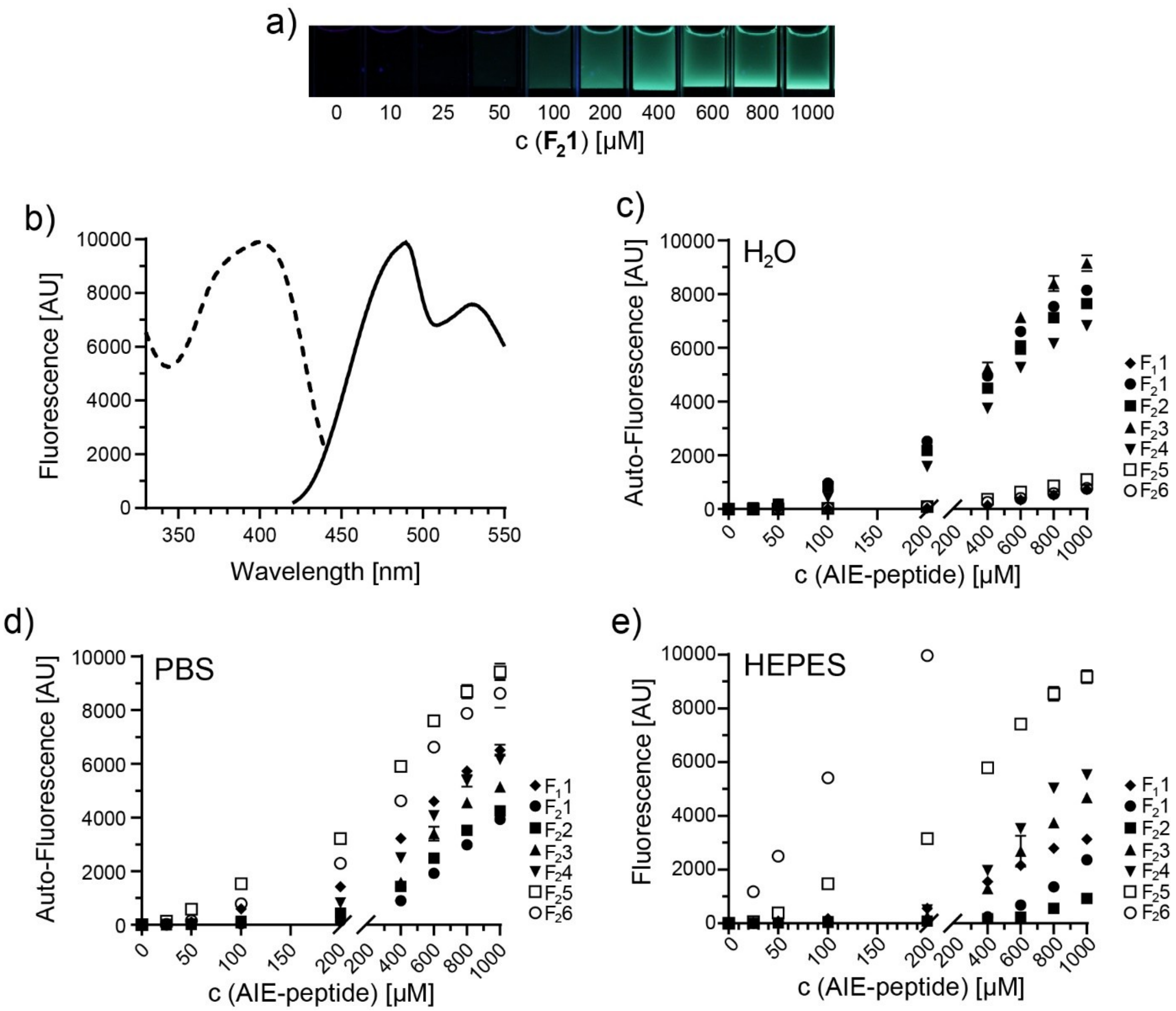

Fig. S1 Autofluorescence properties of peptide-conjugated AIE fluorophores. (a) Photograph of cuvettes with **$F_2$1** at different concentrations in H_2O under UV light. Note that at higher concentrations, autofluorescence increases and fluorescent molecules accumulate at the bottom of the cuvette, despite the solution looking clear. (b) Fluorescence excitation (dotted line) and emission (solid line) spectrum of **$F_2$1**. c) - e) Autofluorescence of **$F_1$1** and **$F_2$1-$F_2$4** plotted as function of AIE-peptide concentration in (c) H_2O, (d) PBS and (e) HEPES buffer. (c-e) 3 technical replicates, error bars indicate the standard error of the average (for several data points, the error bar is smaller than the symbol). For protein binding experiments, the autofluorescence, and thus presence of soluble aggregates, should be as low as possible.

a)

F₁1

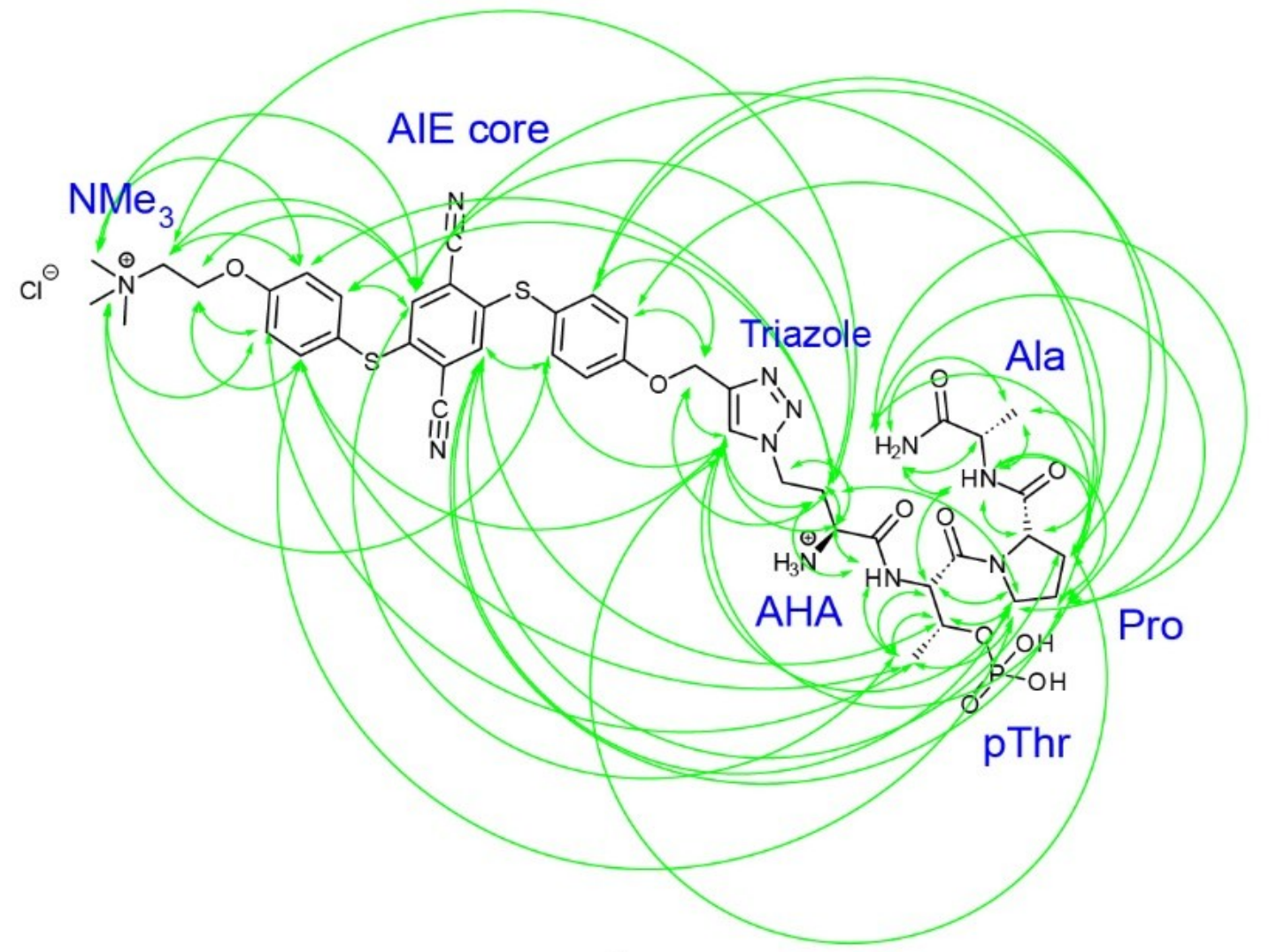

b)

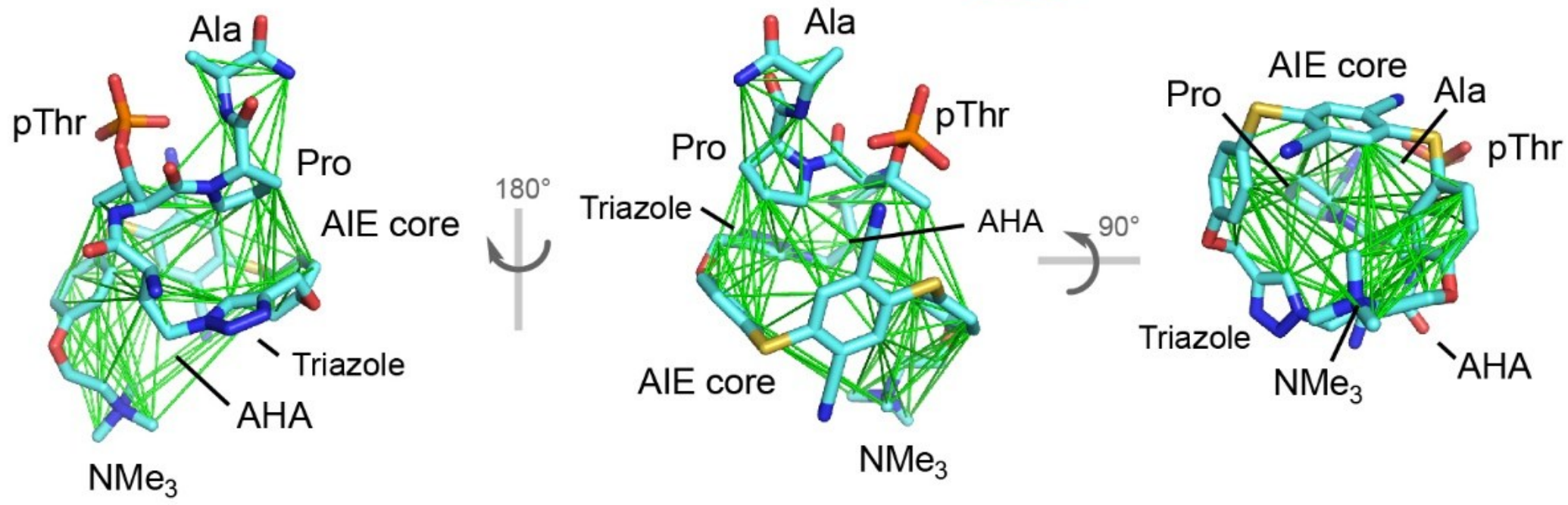

c)

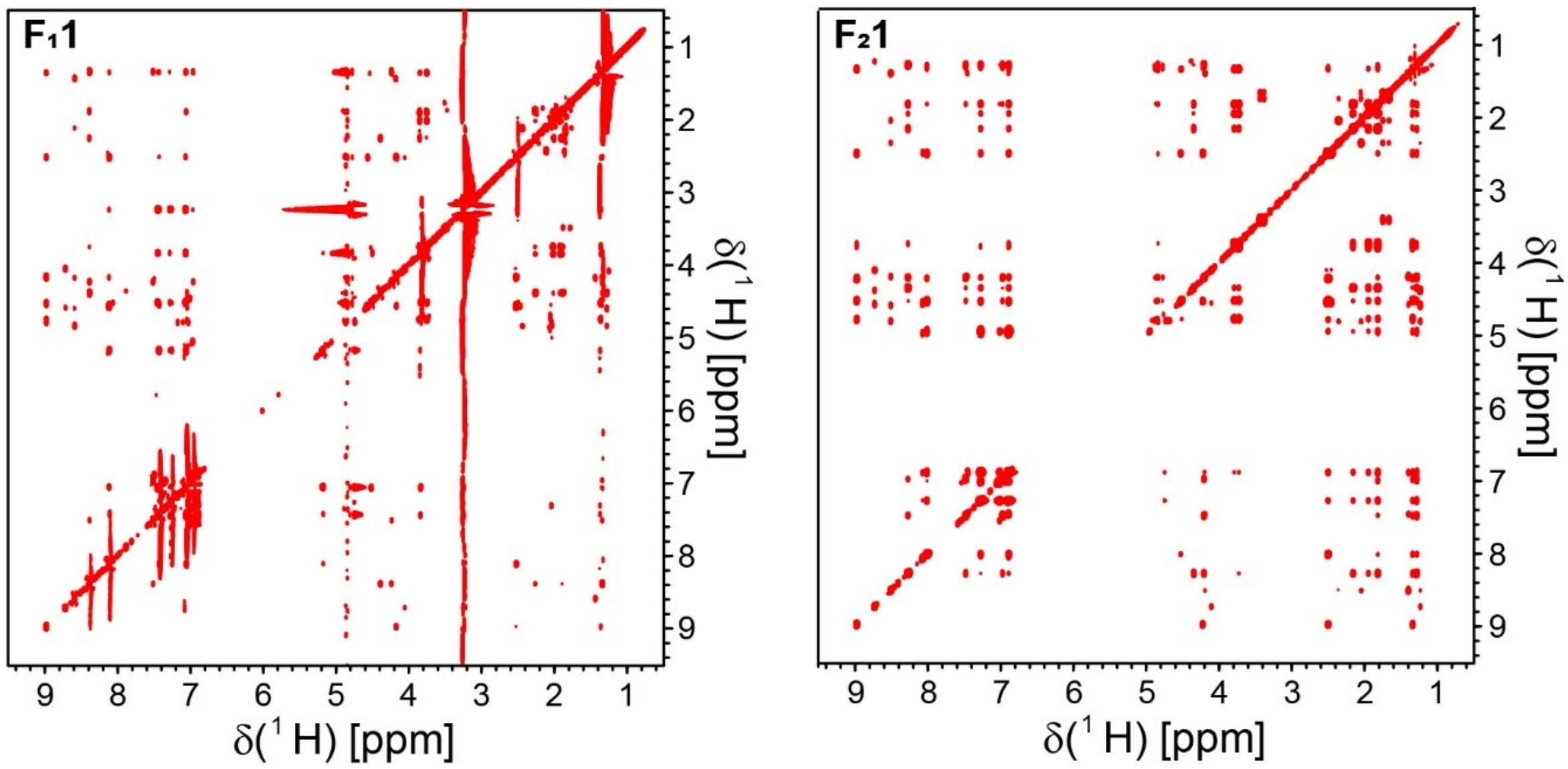

Fig. S2 Chemical structure (a) and solution NMR structure (b) of **F₁1** with NOE restraints mapped as green lines (connecting the corresponding heavy atoms; H atoms not shown for clarity). c) NOESY spectra of **F₁1** with one peptide (left) and **F₂1** with two peptide arms (right) show similar NOE patterns, suggesting that **F₂1** adopts a compact, curled-up structure as well. Due to degenerate signals for the two identical peptide arms the structure for **F₂1** cannot be calculated.

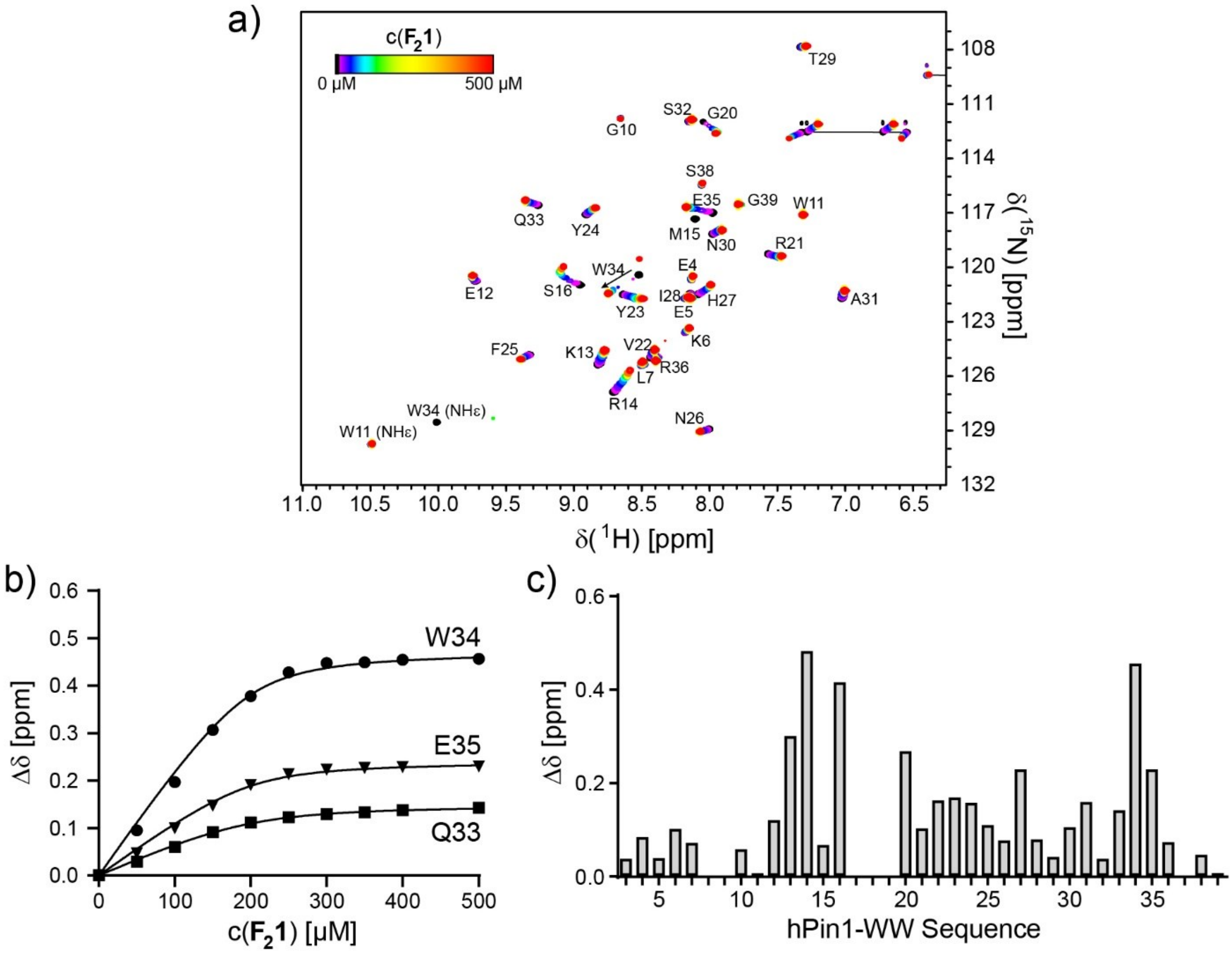

Fig. S3 Specific binding of *h*Pin1-WW to AIE-conjugated peptide $\mathbf{F_21}$. (a) NMR titration of ^{15}N-labelled *h*Pin1-WW (400 µM, 1 replicate) with unlabelled $\mathbf{F_21}$. Overlay of ^{15}N-HSQC spectra, colour-coded by $\mathbf{F_21}$ concentration. Shifting of signals indicates that the corresponding residues are involved in ligand binding. (b) Binding curves for selected residues from the NMR titration. The equivalence point lies at 200 µM because 2 proteins can bind to one two-armed $\mathbf{F_21}$ AIE-peptide. (c) Chemical shift perturbation $\Delta\delta$ by residue of *h*Pin1-WW with 1 mM $\mathbf{F_21}$ compared to *h*Pin1-WW alone.

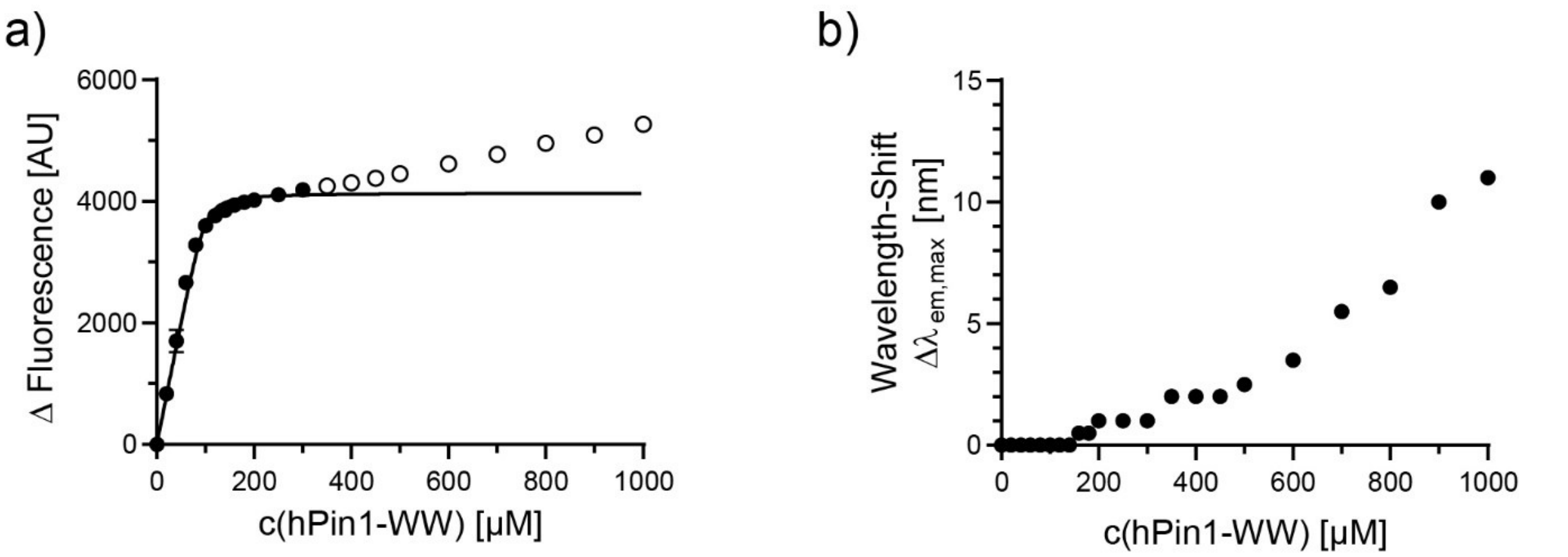

Fig. S4 Fluorescence titration of AIE-peptide $\mathbf{F_21}$ with *h*Pin1-WW (3 technical replicates, error bars indicate the standard error of the average; for most data points, the error bar is smaller than the symbol). (a) Data points up to 300 µM can be fitted (closed circles, fit shown as line). After reaching saturation (open circles), the fluorescence signal increases further and is accompanied by a hypsochromic shift of the emission maximum (b), indicating the formation of soluble aggregates.

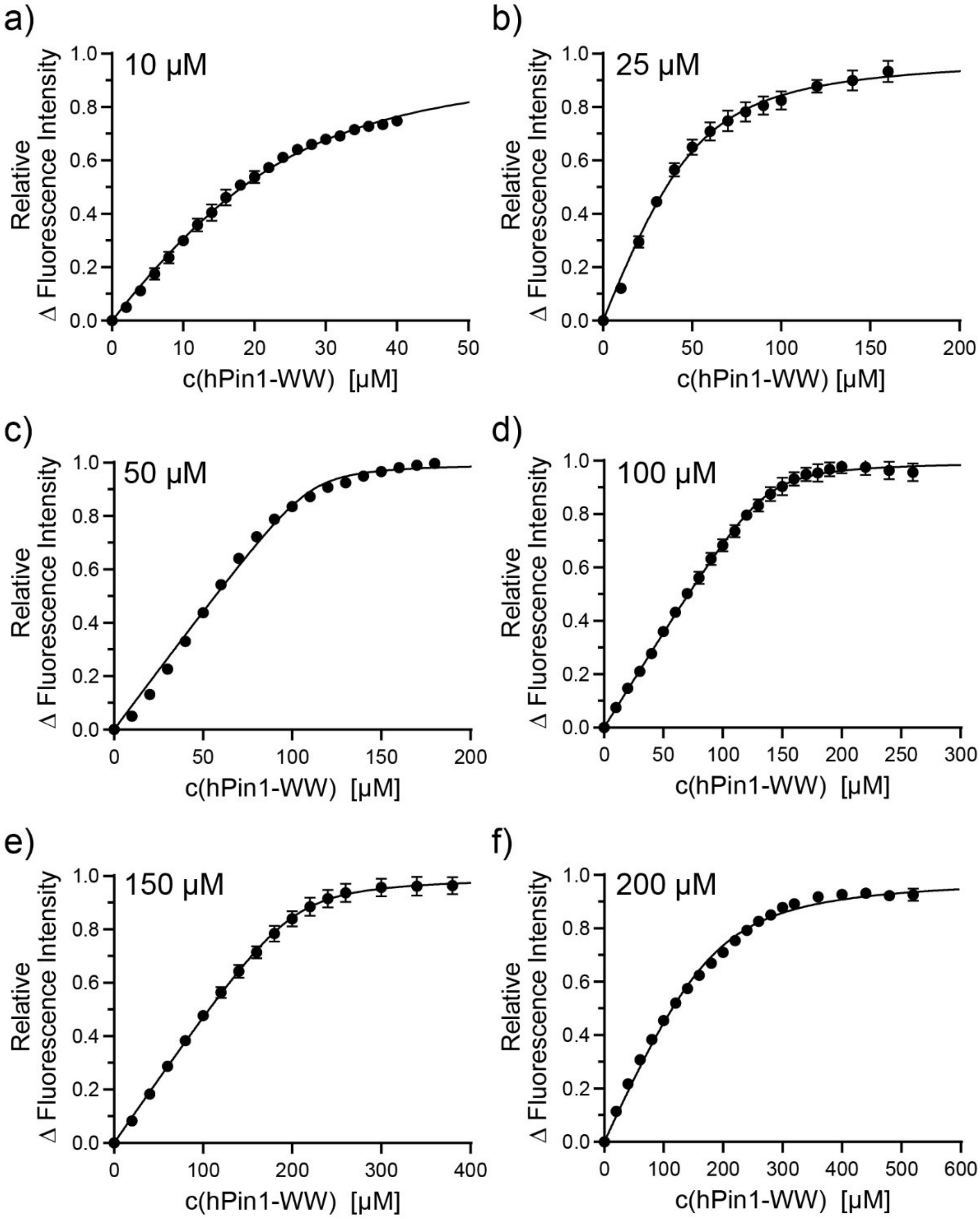

Fig. S5 Fluorescence Titration of AIE-peptide $\mathbf{F_2 1}$ with *h*Pin1-WW at different $\mathbf{F_2 1}$ concentrations. (a) 10 µM, (b) 25 µM, (c) 50 µM, (d) 100 µM, (e) 150 µM, (f) 200 µM. Apparent $K_{D,app}$ and stoichiometry factors (n) are listed in Table S3. 3 technical replicates, error bars indicate the standard error of the average; for some data points, the error bar is smaller than the symbol.

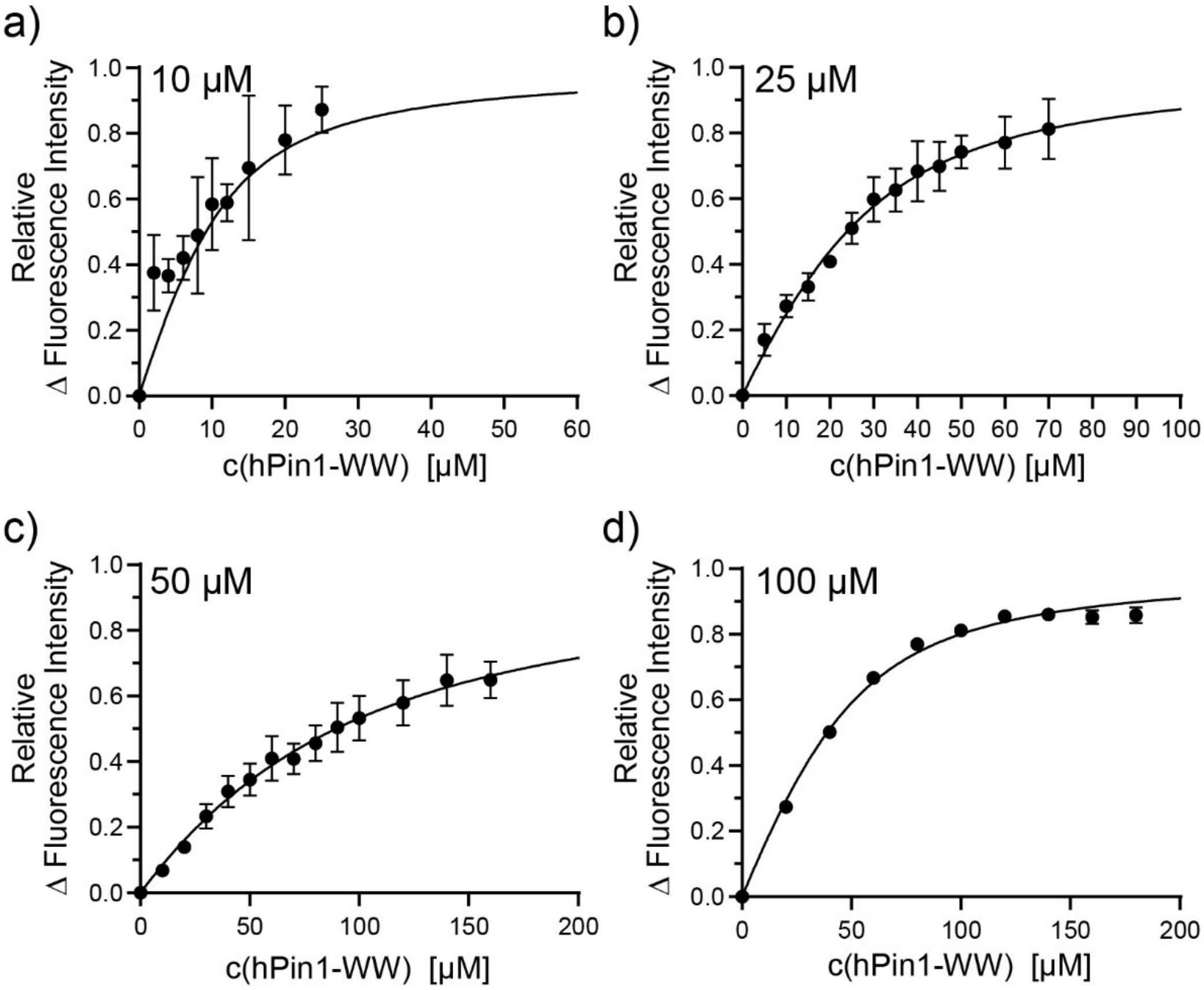

Fig. S6 Fluorescence Titration of AIE-peptide $\mathbf{F_1 1}$ with *h*Pin1-WW at different $\mathbf{F_1 1}$ concentrations. (a) 10 µM, (b) 25 µM, (c) 50 µM, (d) 100 µM. Apparent $K_{D,app}$ and stoichiometry factors (n) are listed in Table S3. 3 technical replicates, error bars indicate the standard error of the average; for most some data points, the error bar is smaller than the symbol.

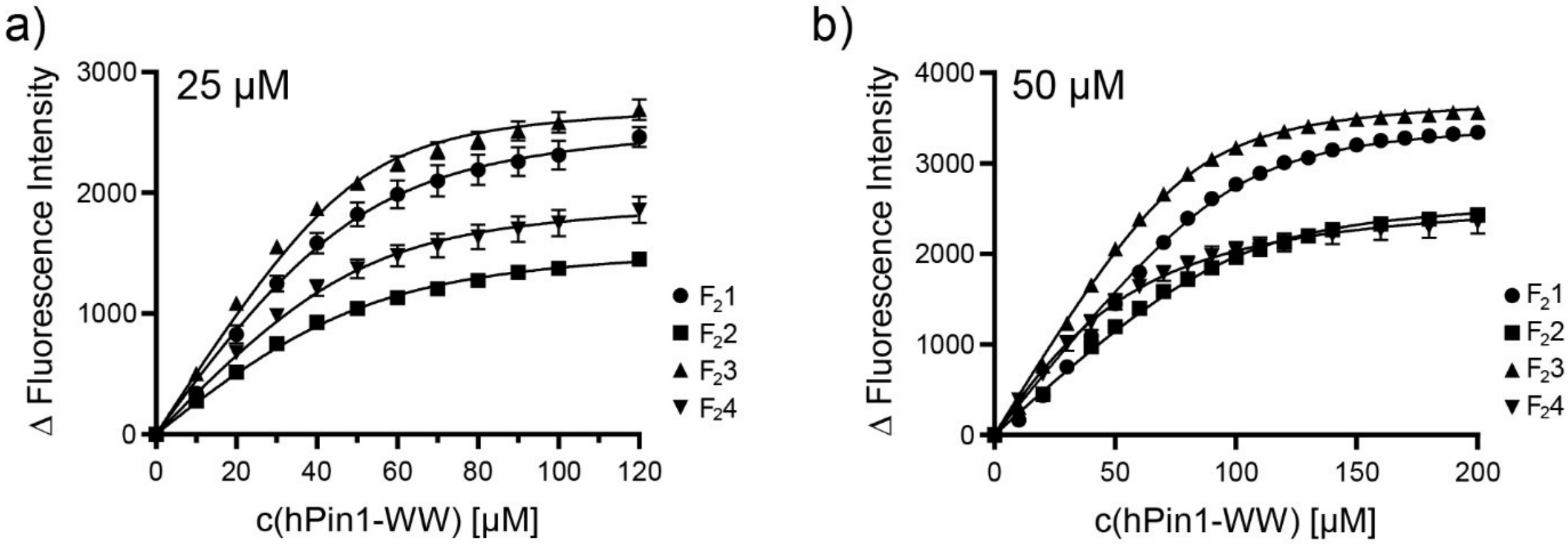

Fig. S7 Dependence of *h*Pin1-WW binding curves on the distance between the pThr-Pro binding motif for two-armed fluorophores $\mathbf{F_2 1}$-$\mathbf{F_2 4}$ at (a) 25 µM and (b) 50 µM fluorophore concentrations. Apparent $K_{D,app}$ and stoichiometry factors (n) are listed in Table S3. 3 technical replicates, error bars indicate the standard error of the average; for some data points, the error bar is smaller than the symbol.

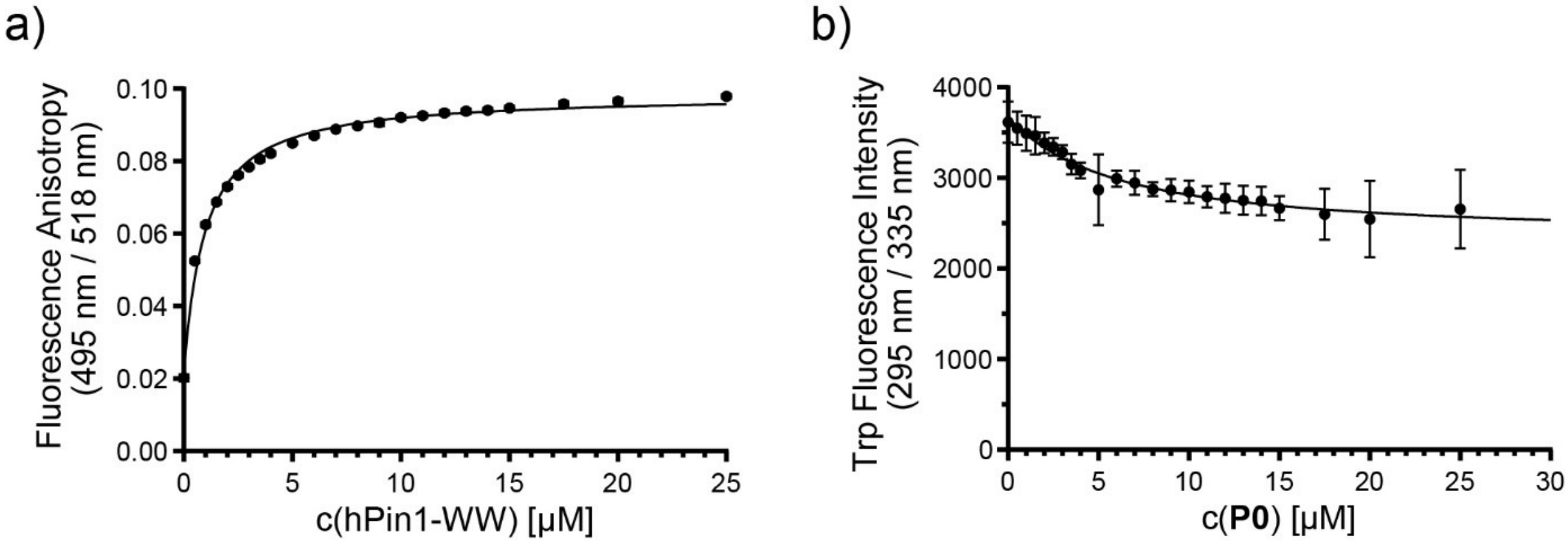

Fig. S8 Fluorescence control titrations. (a) Fluorescence anisotropy titration of control peptide **FAM-P0** (100 nM; labelled with the classical 5,6-FAM fluorophore) with hPin1-WW, fitted with a fixed stoichiometry factor n = 1. (b) Tryptophan fluorescence titration of hPin1-WW (1 µM) with control peptide **P0**, fitted with a fixed stoichiometry factor n = 1. 3 technical replicates, error bars indicate the standard error of the average; for most data points in (a), the error bar is smaller than the symbol.

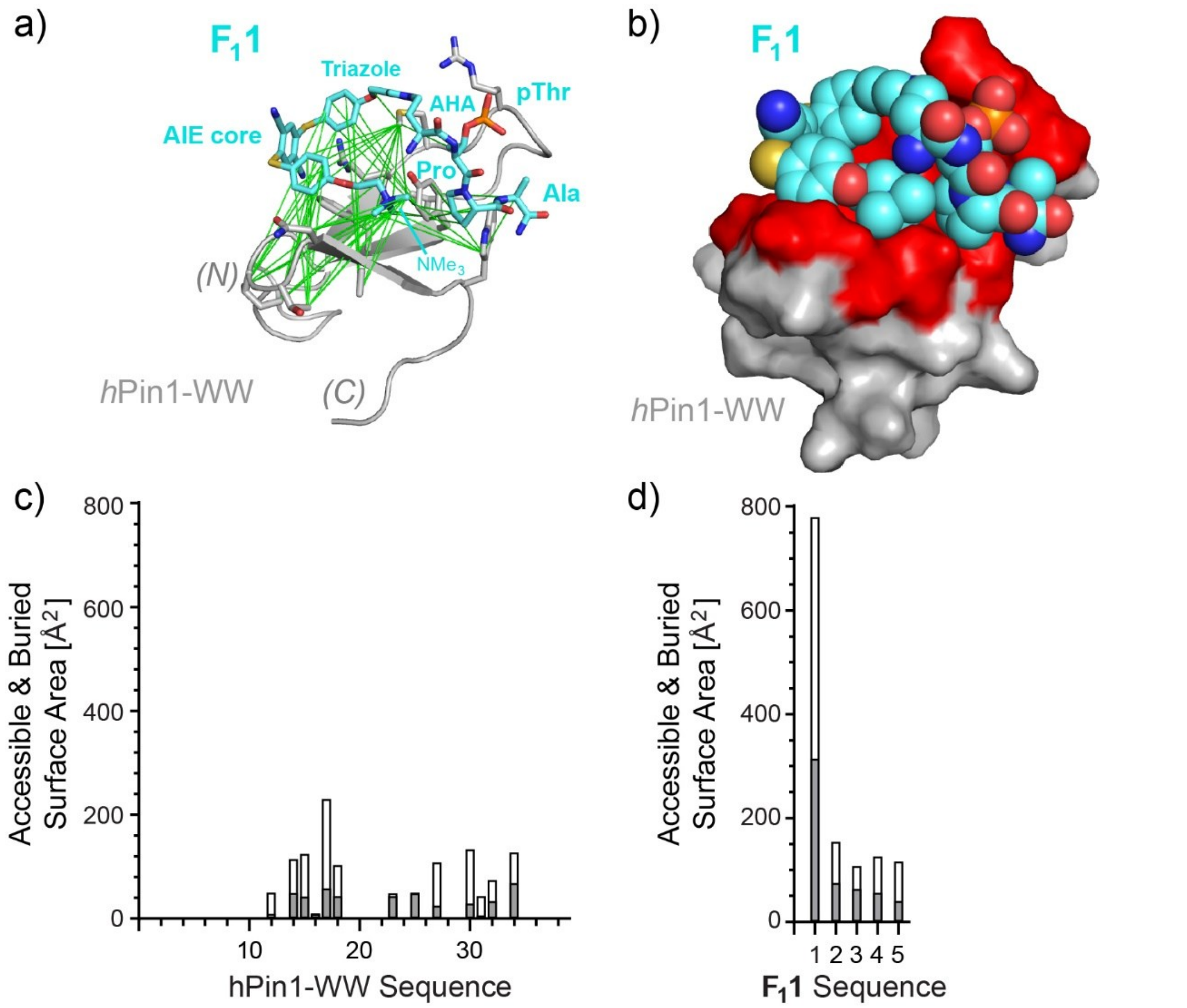

Fig. S9 *h*Pin1-WW:$\mathbf{F_1 1}$ complex interface. a) Solution NMR structure of the hPin1-WW:$\mathbf{F_1 1}$ complex with intermolecular NOE restraints mapped as green lines (connecting the corresponding heavy atoms; H atoms not shown for clarity). b) Surface representation of *h*Pin1-WW (interface residues identified by PISA highlighted in red) with sphere model of $\mathbf{F_1 1}$. c+d) Accessible (ASA, white bars) and buried (BSA, grey bars) surface area for *h*Pin1-WW (c) and $\mathbf{F_1 1}$ (d) residues.

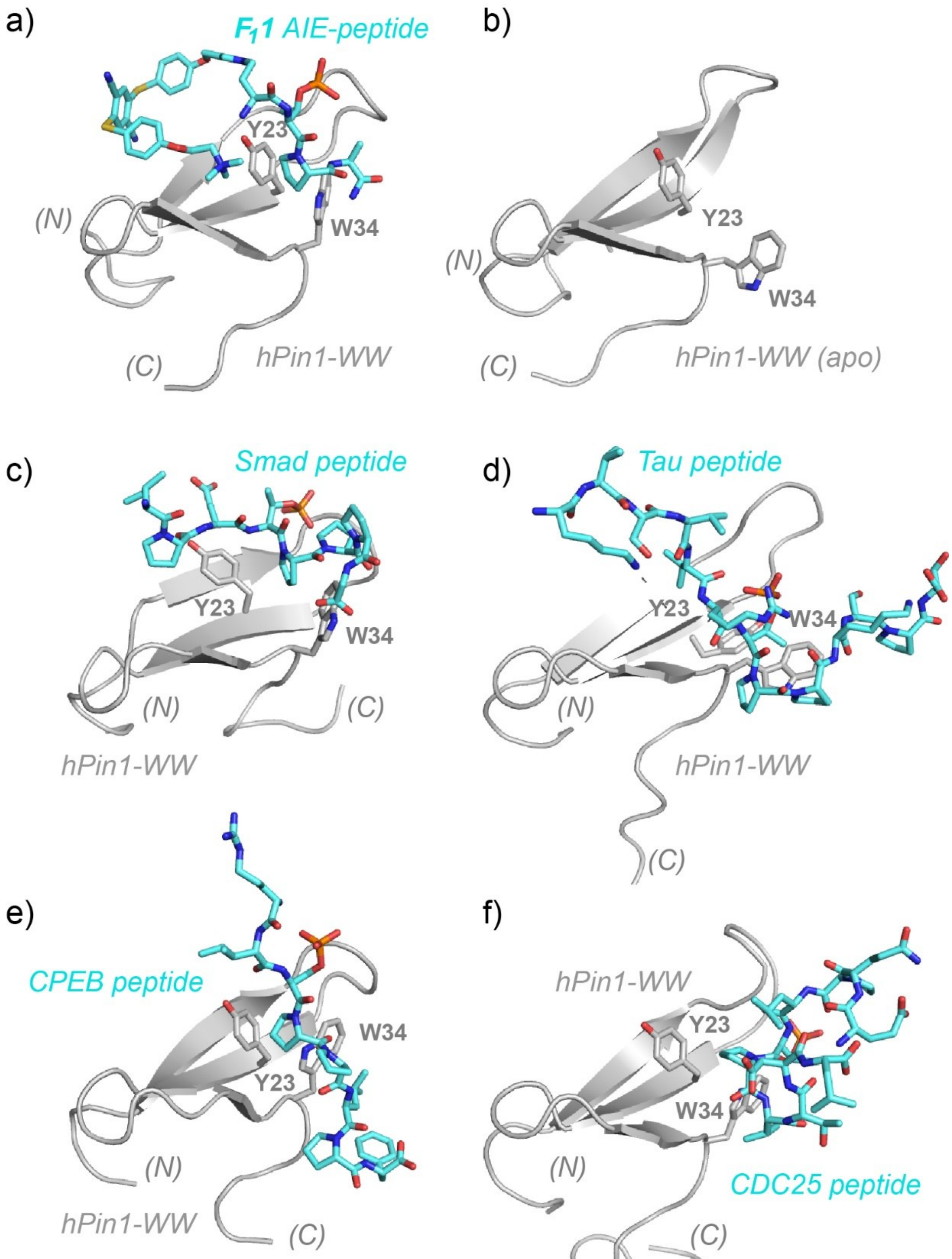

Fig. S10 Comparison of the *h*Pin1-WW: $\mathbf{F_1 1}$ complex structure (a, pdb 29KQ) to the *h*Pin1-WW apo structure[23] (b, pdb 2M8I) and in complex with the Smad peptide[24] (c, pdb 2LB3), Tau peptide[25] (d, pdb 1I8H), CPEB peptide[26] (e, pdb 2N1O) and CDC25 peptide[25] (f, pdb 1I8G). hPin1-WW shown as grey cartoon with proline binding residues Y23 and W34 shown as sticks, bound peptide or AIE peptide shown as cyan sticks.

Non-standard Residue Parameter Files (CYANA Library Files)

AIE-conjugated homoalanine (DS)

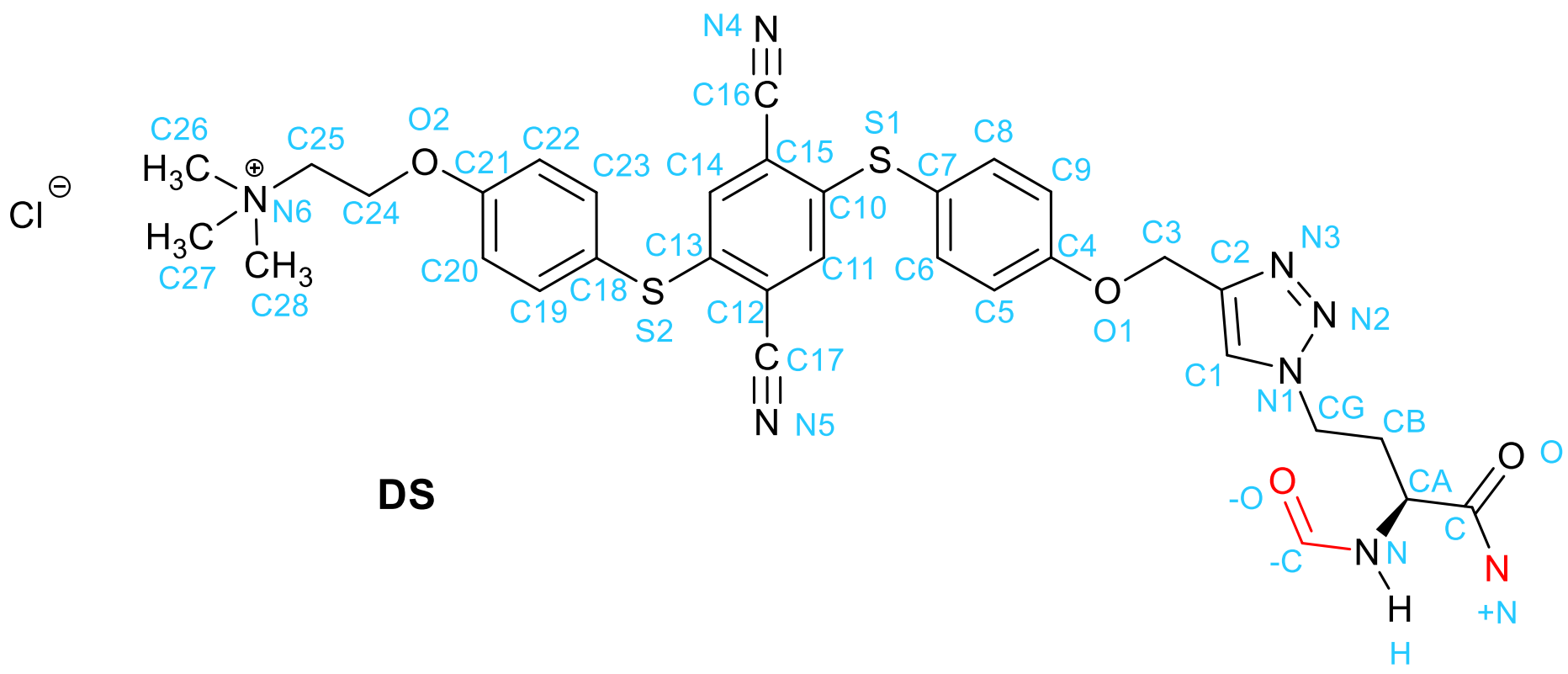

Fig. S0-15. Residue DS with heteroatom nomenclature (light blue) and overlap atoms (red). For H atom nomenclature see structure formula of AIE-peptide $\mathbf{F_11}$ in the compound section above.

```
RESIDUE      DS     20     95      3     94
    1 OMEGA     0      0     0.0000     2      1      3      4      0
    2 PHI       0      0     0.0000     1      3      5     93      0
    3 PSI       0      0     0.0000     3      5     93     95      0
    4 CHI1      0      0     0.0000     3      5      7     11     92
    5 CHI2      0      0     0.0000     5      7     11     15     92
    6 CHI3      0      0     0.0000     7     11     15     18     92
    7 CHI4      0      0     0.0000    18     20     21     25     92
    8 CHI5      0      0     0.0000    20     21     25     26     92
    9 CHI6      0      0     0.0000    21     25     26     27     92
   10 CHI7      0      0     0.0000    29     31     39     40     92
   11 CHI8      0      0     0.0000    31     39     40     41     92
   12 CHI9      0      0     0.0000    43     46     53     54     92
   13 CHI10     0      0     0.0000    46     53     54     55     92
   14 CHI11     0      0     0.0000    57     59     67     68     92
   15 CHI12     0      0     0.0000    59     67     68     72     92
   16 CHI13     0      0     0.0000    67     68     72     76     92
   17 CHI14     0      0     0.0000    68     72     76     77     92
   18 CHI15     0      0     0.0000    72     76     77     78     80
   19 CHI16     0      0     0.0000    72     76     82     83     85
   20 CHI17     0      0     0.0000    72     76     87     88     90
    1 C      C_BYL     0     0.0000     0.0000     0.0000     0.0000     2     3     0     0     0
    2 O      O_BYL     0     0.0000    -0.6068     0.0000    -1.0502     1     0     0     0     0
    3 N      N_AMO     0     0.0000     1.3478     0.0000     0.0000     1     4     5     0     0
    4 H      H_AMI     0     0.0000     1.8331     0.0002     0.8390     3     0     0     0     0
    5 CA     C_ALI     0     0.0000     2.0800     0.0008    -1.2694     3     6     7    93     0
    6 HA     H_ALI     0     0.0000     1.5316     0.5898    -2.0051     5     0     0     0     0
    7 CB     C_ALI     0     0.0000     3.4674     0.6111    -1.0577     5     8     9    11     0
    8 HB2    H_ALI     0     0.0000     4.0435     0.5309    -1.9786     7     0     0     0    10
    9 HB3    H_ALI     0     0.0000     3.9808     0.0765    -0.2595     7     0     0     0    10
   10 QB     PSEUD     0     0.0000     4.0122     0.3037    -1.1191     0     0     0     0     0
   11 CG     C_ALI     0     0.0000     3.3220     2.0844    -0.6727     7    12    13    15     0
   12 HG2    H_ALI     0     0.0000     2.7459     2.1646     0.2482    11     0     0     0    14
   13 HG3    H_ALI     0     0.0000     2.8079     2.6195    -1.4715    11     0     0     0    14
   14 QG     PSEUD     0     0.0000     2.7769     2.3921    -0.6117     0     0     0     0     0
   15 N1     N_AMO     0     0.0000     4.6509     2.6689    -0.4708    11    16    18     0     0
   16 N2     N_AMO     0     0.0000     5.5557     2.2246     0.3294    15    17     0     0     0
   17 N3     N_AMO     0     0.0000     6.6062     2.9638     0.2638    16    20     0     0     0
   18 C1     C_ARO     0     0.0000     5.1360     3.7682    -1.0920    15    19    20     0     0
   19 H1     H_ARO     0     0.0000     4.6263     4.3780    -1.8233    18     0     0     0     0
   20 C2     C_BYL     0     0.0000     6.3883     3.9510    -0.6114    17    21    18     0     0
   21 C3     C_ALI     0     0.0000     7.3489     5.0470    -0.9933    20    22    23    25     0
   22 H31    H_ALI     0     0.0000     8.2544     4.6075    -1.4115    21     0     0     0    24
   23 H32    H_ALI     0     0.0000     7.6049     5.6305    -0.1083    21     0     0     0    24
   24 Q3     PSEUD     0     0.0000     7.9296     5.1190    -0.7599     0     0     0     0     0
```

25	O1	O_EST	0	0.0000	6.7388	5.8987	-1.9652	21	26	0	0	0
26	C4	C_ARO	0	0.0000	7.6487	6.8629	-2.2693	25	27	35	0	0
27	C5	C_ARO	0	0.0000	8.8905	6.8644	-1.6506	26	28	29	0	0
28	H5	H_ARO	0	0.0000	9.1346	6.0995	-0.9285	27	0	0	0	37
29	C6	C_ARO	0	0.0000	9.8169	7.8403	-1.9610	27	30	31	0	0
30	H6	H_ARO	0	0.0000	10.7835	7.8405	-1.4794	29	0	0	0	34
31	C7	C_ARO	0	0.0000	9.5008	8.8266	-2.8870	29	39	32	0	0
32	C8	C_ARO	0	0.0000	8.2573	8.8250	-3.5055	31	33	35	0	0
33	H8	H_ARO	0	0.0000	8.0115	9.5898	-4.2276	32	0	0	0	34
34	Q5	PSEUD	0	0.0000	9.3975	8.7152	-2.8535	0	0	0	0	38
35	C9	C_ARO	0	0.0000	7.3340	7.8451	-3.1967	32	36	26	0	0
36	H9	H_ARO	0	0.0000	6.3671	7.8437	-3.6770	35	0	0	0	37
37	Q4	PSEUD	0	0.0000	7.7509	6.9716	-2.3027	0	0	0	0	38
38	QRB	PSEUD	0	0.0000	8.5742	7.8434	-2.5781	0	0	0	0	0
39	S1	S_RED	0	0.0000	10.6790	10.0770	-3.2806	31	40	0	0	0
40	C10	C_ARO	0	0.0000	10.3069	11.2550	-2.0239	39	41	50	0	0
41	C11	C_ARO	0	0.0000	9.3745	10.9527	-1.0455	40	42	43	0	0
42	H11	H_ARO	0	0.0000	8.8751	9.9946	-1.0535	41	0	0	0	49
43	C12	C_ARO	0	0.0000	9.0800	11.8850	-0.0508	41	44	46	0	0
44	C17	C_VIN	0	0.0000	8.1161	11.5731	0.9607	43	45	0	0	0
45	N5	N_AMO	0	0.0000	7.3505	11.3258	1.7631	44	0	0	0	0
46	C13	C_ARO	0	0.0000	9.7316	13.1317	-0.0403	43	53	47	0	0
47	C14	C_ARO	0	0.0000	10.6601	13.4354	-1.0218	46	48	50	0	0
48	H14	H_ARO	0	0.0000	11.1590	14.3927	-1.0139	47	0	0	0	49
49	Q8	PSEUD	0	0.0000	10.0171	12.1936	-1.0337	0	0	0	0	0
50	C15	C_ARO	0	0.0000	10.9575	12.5000	-2.0136	47	51	40	0	0
51	C16	C_VIN	0	0.0000	11.9230	12.8120	-3.0252	50	52	0	0	0
52	N4	N_AMO	0	0.0000	12.6875	13.0600	-3.8274	51	0	0	0	0
53	S2	S_RED	0	0.0000	9.3602	14.3093	1.2170	46	54	0	0	0
54	C18	C_ARO	0	0.0000	10.5645	15.5407	0.8469	53	55	63	0	0
55	C19	C_ARO	0	0.0000	11.8502	15.4330	1.3630	54	56	57	0	0
56	H19	H_ARO	0	0.0000	12.1116	14.5944	1.9907	55	0	0	0	65
57	C20	C_ARO	0	0.0000	12.7946	16.3967	1.0686	55	58	59	0	0
58	H20	H_ARO	0	0.0000	13.7937	16.3128	1.4694	57	0	0	0	62
59	C21	C_ARO	0	0.0000	12.4579	17.4773	0.2668	57	67	60	0	0
60	C22	C_ARO	0	0.0000	11.1738	17.5851	-0.2494	59	61	63	0	0
61	H22	H_ARO	0	0.0000	10.9142	18.4250	-0.8763	60	0	0	0	62
62	Q7	PSEUD	0	0.0000	12.3539	17.3689	0.2965	0	0	0	0	66
63	C23	C_ARO	0	0.0000	10.2296	16.6207	0.0392	60	64	54	0	0
64	H23	H_ARO	0	0.0000	9.2310	16.7055	-0.3615	63	0	0	0	65
65	Q6	PSEUD	0	0.0000	10.6713	15.6499	0.8146	0	0	0	0	66
66	QRC	PSEUD	0	0.0000	11.5126	16.5094	0.5556	0	0	0	0	0
67	O2	O_EST	0	0.0000	13.3867	18.4285	-0.0179	59	68	0	0	0
68	C24	C_ALI	0	0.0000	14.5909	18.0421	0.6484	67	69	70	72	0
69	H242	H_ALI	0	0.0000	14.9107	17.0662	0.2826	68	0	0	0	71
70	H243	H_ALI	0	0.0000	14.4097	17.9866	1.7219	68	0	0	0	71
71	Q24	PSEUD	0	0.0000	14.6602	17.5264	1.0023	0	0	0	0	0
72	C25	C_ALI	0	0.0000	15.6834	19.0756	0.3674	68	73	74	76	0
73	H252	H_ALI	0	0.0000	16.6340	18.7148	0.7594	72	0	0	0	75
74	H253	H_ALI	0	0.0000	15.7690	19.2301	-0.7085	72	0	0	0	75
75	Q25	PSEUD	0	0.0000	16.2015	18.9724	0.0254	0	0	0	0	0
76	N6	N_AMI	0	0.0000	15.3351	20.3452	1.0190	72	77	82	87	0
77	C26	C_ALI	0	0.0000	14.0474	20.8257	0.4984	76	78	79	80	0
78	H261	H_ALI	0	0.0000	13.2466	20.1808	0.8585	77	0	0	0	81
79	H262	H_ALI	0	0.0000	13.8750	21.8457	0.8419	77	0	0	0	81
80	H263	H_ALI	0	0.0000	14.0667	20.8071	-0.5916	77	0	0	0	81
81	Q26	PSEUD	0	0.0000	13.7295	20.9445	0.3696	0	0	0	0	92
82	C27	C_ALI	0	0.0000	16.3780	21.3406	0.7394	76	83	84	85	0
83	H271	H_ALI	0	0.0000	16.4560	21.4939	-0.3375	82	0	0	0	86
84	H272	H_ALI	0	0.0000	16.1203	22.2829	1.2224	82	0	0	0	86
85	H273	H_ALI	0	0.0000	17.3329	20.9848	1.1252	82	0	0	0	86
86	Q27	PSEUD	0	0.0000	16.6364	21.5872	0.6700	0	0	0	0	92
87	C28	C_ALI	0	0.0000	15.2307	20.1395	2.4687	76	88	89	90	0
88	H281	H_ALI	0	0.0000	16.1858	19.7823	2.8550	87	0	0	0	91
89	H282	H_ALI	0	0.0000	14.9720	21.0812	2.9524	87	0	0	0	91
90	H283	H_ALI	0	0.0000	14.4564	19.4003	2.6761	87	0	0	0	91
91	Q28	PSEUD	0	0.0000	15.2047	20.0879	2.8278	0	0	0	0	92
92	QQN	PSEUD	0	0.0000	15.1902	20.8732	1.2891	0	0	0	0	0
93	C	C_BYL	0	0.0000	2.2238	-1.4139	-1.7664	5	94	95	0	0
94	O	O_BYL	0	0.0000	1.7613	-2.3332	-1.1240	93	0	0	0	0
95	N	N_AMO	0	0.0000	2.8674	-1.6597	-2.9256	93	0	0	0	0

Phosphothreonine (TPO)

TPO

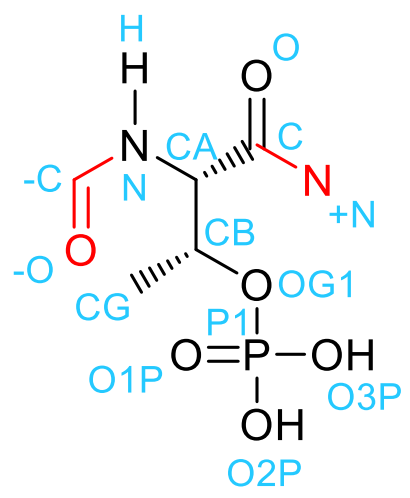

Fig. S0-16. Residue TPO with heteroatom nomenclature (light blue) and overlap atoms (red). For H atom nomenclature see structure formula of AIE-peptide **$F_1$1** in the compound section above.

RESIDUE	TPO	9	23	3	22							
1	OMEGA	0	0	0.0000	2	1	3	5	0			
2	PHI	0	0	0.0000	1	3	5	21	0			
3	CHI1	0	0	0.0000	3	5	7	9	20			
4	CHI2	0	0	0.0000	5	7	9	10	13			
5	CHI3	0	0	0.0000	5	7	14	15	20			
6	CHI4	0	0	0.0000	7	14	15	16	20			
7	CHI5	0	0	0.0000	14	15	17	18	18			
8	CHI6	0	0	0.0000	14	15	19	20	20			
9	PSI	0	0	0.0000	3	5	21	23	0			
1	C	C_BYL	0	0.0000	0.0000	0.0000	0.0000	2	3	0	0	0
2	O	O_BYL	0	0.0000	-0.6064	0.0000	-1.0502	1	0	0	0	0
3	N	N_AMI	0	0.0000	1.3478	0.0000	0.0000	1	4	5	0	0
4	H	H_AMI	0	0.0000	1.8328	0.0001	0.8401	3	0	0	0	0
5	CA	C_ALI	0	0.0000	2.0803	0.0000	-1.2687	3	6	7	21	0
6	HA	H_ALI	0	0.0000	1.5328	0.5894	-2.0043	5	0	0	0	0
7	CB	C_ALI	0	0.0000	3.4674	0.6097	-1.0568	5	8	9	14	0
8	HB	H_ALI	0	0.0000	3.9788	0.6914	-2.0160	7	0	0	0	0
9	CG	C_ALI	0	0.0000	3.3238	2.0004	-0.4353	7	10	11	12	0
10	HG21	H_ALI	0	0.0000	2.8124	1.9187	0.5238	9	0	0	0	13
11	HG22	H_ALI	0	0.0000	4.3120	2.4348	-0.2844	9	0	0	0	13
12	HG23	H_ALI	0	0.0000	2.7446	2.6386	-1.1028	9	0	0	0	13
13	QG	PSEUD	0	0.0000	3.2897	2.3306	-0.2878	0	0	0	0	0
14	OG1	O_EST	0	0.0000	4.2268	-0.2269	-0.1819	7	15	0	0	0
15	P1	P_ALI	0	0.0000	5.5306	-0.6958	-1.0019	14	16	17	19	0
16	O1P	O_BYL	0	0.0000	6.2504	0.4947	-1.5068	15	0	0	0	0
17	O2P	O_HYD	0	0.0000	6.5007	-1.5379	-0.0314	15	18	0	0	0
18	H2P	H_OXY	0	0.0000	7.2670	-1.7989	-0.5603	17	0	0	0	0
19	O3P	O_HYD	0	0.0000	5.0812	-1.6176	-2.2429	15	20	0	0	0
20	H3P	H_OXY	0	0.0000	4.6170	-2.3801	-1.8711	19	0	0	0	0
21	C	C_BYL	0	0.0000	2.2242	-1.4155	-1.7656	5	22	23	0	0
22	O	O_BYL	0	0.0000	1.7606	-2.3338	-1.1232	21	0	0	0	0
23	N	N_AMI	0	0.0000	2.8681	-1.6608	-2.9240	21	0	0	0	0

Amidated C-terminus (NH2)

NH2

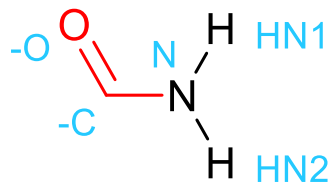

Fig. S0-17. Residue NH2 with atom nomenclature (light blue) and overlap atoms (red).

```
RESIDUE    NH2       1     5     3     5
   1 OMEGA     0     0     0.0000     2     1     3     5     0
   1 C     C_BYL     0     0.0000     0.0000     0.0000     0.0000     2     3     0     0     0
   2 O     O_BYL     0     0.0000    -0.6064     0.0000    -1.0504     1     0     0     0     0
   3 N     N_AMI     0     0.0000     1.3478     0.0000     0.0000     1     4     5     0     0
   4 HN1   H_AMI     0     0.0000     1.8327     0.0000     0.8402     3     0     0     0     0
   5 HN2   H_AMI     0     0.0000     1.8328    -0.0040    -0.8399     3     0     0     0     0
```

References

1 K. Hommel, A.-M. A. Kauth, A. Kirupakaran, S. Theisen, M. Hayduk, F. C. Niemeyer, C. Beuck, R. Zadmard, P. Bayer, B. Jan Ravoo, J. Voskuhl, T. Schrader and S. K. Knauer, Functional Linkers Support Targeting of Multivalent Tweezers to Taspase1, *Chemistry (Weinheim an der Bergstrasse, Germany)*, 2024, **30**, e202401542.

2 K. Klein, M. Hayduk, S. Kollenda, M. Schmiedtchen, J. Voskuhl and M. Epple, Covalent Attachment of Aggregation-Induced Emission Molecules to the Surface of Ultrasmall Gold Nanoparticles to Enhance Cell Penetration, *Molecules (Basel, Switzerland)*, 2022, **27**. DOI: 10.3390/molecules27061788.

3 M. Meldal and C. W. Tornøe, Cu-catalyzed azide-alkyne cycloaddition, *Chemical reviews*, 2008, **108**, 2952–3015.

4 B. T. Worrell, J. A. Malik and V. V. Fokin, Direct evidence of a dinuclear copper intermediate in Cu(I)-catalyzed azide-alkyne cycloadditions, *Science (New York, N.Y.)*, 2013, **340**, 457–460.

5 A. Hogeweg, A. Sowislok, T. Schrader and C. Beuck, An NMR Method To Pinpoint Supramolecular Ligand Binding to Basic Residues on Proteins, *Angewandte Chemie (International ed. in English)*, 2017, **56**, 14758–14762.

6 T. D. Goddart and D. G. Kneller, University of California, San Francisco, CA, 2008.

7 R. Keller, *The Computer Aided Resonance Assignment Tutorial*, Cantina Verlag, Goldau, Switzerland, 1st edn., 2004.

8 D. S. Wishart, C. G. Bigam, J. Yao, F. Abildgaard, H. J. Dyson, E. Oldfield, J. L. Markley and B. D. Sykes, 1H, 13C and 15N chemical shift referencing in biomolecular NMR, *Journal of biomolecular NMR*, 1995, **6**, 135–140.

9 A. Ayed, F. A. Mulder, G. S. Yi, Y. Lu, L. E. Kay and C. H. Arrowsmith, Latent and active p53 are identical in conformation, *Nature structural biology*, 2001, **8**, 756–760.

10 C. Zwahlen, P. Legault, S. J. F. Vincent, J. Greenblatt, R. Konrat and L. E. Kay, Methods for Measurement of Intermolecular NOEs by Multinuclear NMR Spectroscopy: Application to a Bacteriophage λ N-Peptide/ boxB RNA Complex, *J. Am. Chem. Soc.*, 1997, **119**, 6711–6721.

11 K. Ogura, H. Terasawa and F. Inagaki, An improved double-tuned and isotope-filtered pulse scheme based on a pulsed field gradient and a wide-band inversion shaped pulse, *Journal of biomolecular NMR*, 1996, **8**, 492–498.

12 J. Iwahara, J. M. Wojciak and R. T. Clubb, Improved NMR spectra of a protein-DNA complex through rational mutagenesis and the application of a sensitivity optimized isotope-filtered NOESY experiment, *Journal of biomolecular NMR*, 2001, **19**, 231–241.

13 A. L. Breeze, Isotope-filtered NMR methods for the study of biomolecular structure and interactions, *Progress in Nuclear Magnetic Resonance Spectroscopy*, 2000, **36**, 323–372.
14 G. Otting and K. Wüthrich, Heteronuclear filters in two-dimensional 1H,1H-NMR spectroscopy: combined use with isotope labelling for studies of macromolecular conformation and intermolecular interactions, *Quarterly reviews of biophysics*, 1990, **23**, 39–96.
15 G. Otting and K. Wüthrich, Extended heteronuclear editing of 2D 1H NMR spectra of isotope-labeled proteins, using the X(ω1, ω2) double half filter, *Journal of Magnetic Resonance (1969)*, 1989, **85**, 586–594.
16 P. Güntert, Automated NMR structure calculation with CYANA, *Methods in molecular biology (Clifton, N.J.)*, 2004, **278**, 353–378.
17 G. Cornilescu, F. Delaglio and A. Bax, Protein backbone angle restraints from searching a database for chemical shift and sequence homology, *Journal of biomolecular NMR*, 1999, **13**, 289–302.
18 Y. Shen, F. Delaglio, G. Cornilescu and A. Bax, TALOS+: a hybrid method for predicting protein backbone torsion angles from NMR chemical shifts, *Journal of biomolecular NMR*, 2009, **44**, 213–223.
19 T. Herrmann, P. Güntert and K. Wüthrich, Protein NMR structure determination with automated NOE assignment using the new software CANDID and the torsion angle dynamics algorithm DYANA, *Journal of molecular biology*, 2002, **319**, 209–227.
20 T. Herrmann, P. Güntert and K. Wüthrich, Protein NMR structure determination with automated NOE-identification in the NOESY spectra using the new software ATNOS, *Journal of biomolecular NMR*, 2002, **24**, 171–189.
21 P. Serrano, B. Pedrini, B. Mohanty, M. Geralt, T. Herrmann and K. Wüthrich, The J-UNIO protocol for automated protein structure determination by NMR in solution, *Journal of biomolecular NMR*, 2012, **53**, 341–354.
22 E. Krissinel and K. Henrick, Inference of macromolecular assemblies from crystalline state, *Journal of molecular biology*, 2007, **372**, 774–797.
23 L. M. Luh, R. Hänsel, F. Löhr, D. K. Kirchner, K. Krauskopf, S. Pitzius, B. Schäfer, P. Tufar, I. Corbeski, P. Güntert and V. Dötsch, Molecular crowding drives active Pin1 into nonspecific complexes with endogenous proteins prior to substrate recognition, *Journal of the American Chemical Society*, 2013, **135**, 13796–13803.
24 E. Aragón, N. Goerner, A.-I. Zaromytidou, Q. Xi, A. Escobedo, J. Massagué and M. J. Macias, A Smad action turnover switch operated by WW domain readers of a phosphoserine code, *Genes & development*, 2011, **25**, 1275–1288.
25 R. Wintjens, J. M. Wieruszeski, H. Drobecq, P. Rousselot-Pailley, L. Buée, G. Lippens and I. Landrieu, 1H NMR study on the binding of Pin1 Trp-Trp domain with phosphothreonine peptides, *The Journal of biological chemistry*, 2001, **276**, 25150–25156.
26 C. Schelhorn, P. Martín-Malpartida, D. Suñol and M. J. Macias, Structural Analysis of the Pin1-CPEB1 interaction and its potential role in CPEB1 degradation, *Scientific reports*, 2015, **5**, 14990.
